# Characterization, comparative, and functional analysis of arylacetamide deacetylase from Gnathostomata organisms

**DOI:** 10.1186/s43141-022-00443-z

**Published:** 2022-12-21

**Authors:** Tania Diaz-Vidal, Christian Berenice Romero-Olivas, Raúl Balam Martínez-Pérez

**Affiliations:** 1 Present Address: Department of Chemical Engineering, University of Guadalajara, 44430 Guadalajara, Mexico; 2 Present Address: Department of Biotechnology and Food Sciences, Instituto Tecnológico de Sonora, Ciudad Obregón, Mexico 85137; 3 Industrial Biotechnology, Centro de Investigación y Asistencia en Tecnología y Diseño del Estado de Jalisco, 45019 Zapopan, Mexico

**Keywords:** Arylacetamide deacetylase, AADAC, Orthologues, Interactome, Computational analysis

## Abstract

**Background:**

Arylacetamide deacetylase (AADAC) is a lipolytic enzyme involved in xenobiotic metabolism. The characterization in terms of activity and substrate preference has been limited to a few mammalian species. The potential role and catalytic activities of AADAC from other organisms are still poorly understood. Therefore, in this work, the physicochemical properties, proteomic analysis, and protein-protein interactions from Gnathostomata organisms were investigated.

**Results:**

The analysis were performed with 142 orthologue sequences with ~ 48–100% identity with human AADAC. The catalytic motif HGG[A/G] tetrapeptide block was conserved through all AADAC orthologues. Four variations were found in the consensus pentapeptide GXSXG sequence (GDSAG, GESAG, GDSSG, and GSSSG), and a novel motif YXLXP was found. The prediction of *N*-glycosylation sites projected 4, 1, 6, and 4 different patterns for amphibians, birds, mammals, and reptiles, respectively. The transmembrane regions of AADAC orthologues were not conserved among groups, and variations in the number and orientation of the active site and *C*-terminal carboxyl were observed among the sequences studied. The protein-protein interaction of AADAC orthologues were related to cancer, lipid, and xenobiotic metabolism genes.

**Conclusion:**

The findings from this computational analysis offer new insight into one of the main enzymes involved in xenobiotic metabolism from mammals, reptiles, amphibians, and birds and its potential use in medical and veterinarian biotechnological approaches.

**Graphical Abstract:**

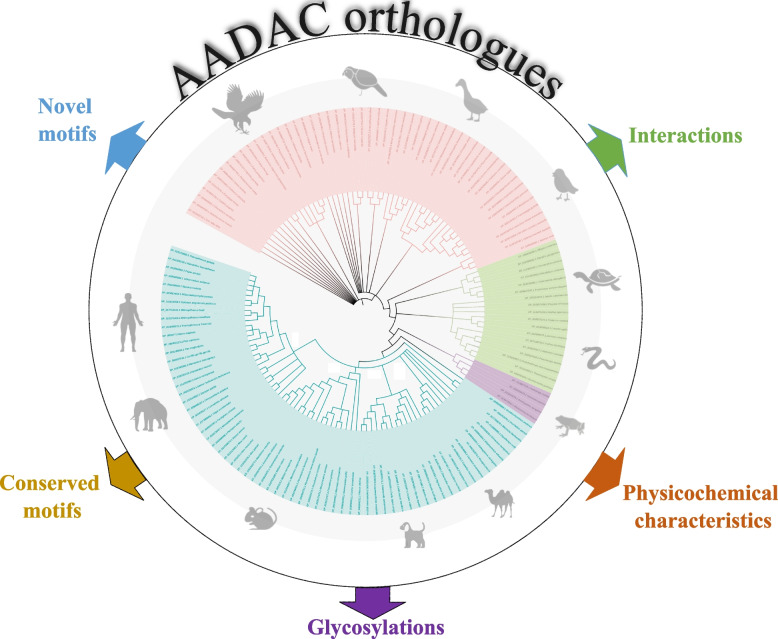

**Supplementary Information:**

The online version contains supplementary material available at 10.1186/s43141-022-00443-z.

## Background

Arylacetamide deacetylase (AADAC) is a lipolytic enzyme involved mainly in the hydrolysis of esters and amides [1–3]. Its active site domain is a classic lipase/esterase GXSXG motif (catalytic triad Ser189, His373, and Asp343), sharing high sequence homology with the active site of hormone-sensitive lipases (HSL). Although AADAC was initially considered as an esterase, current findings suggest that AADAC could be classified as a lipase, due to the strong homology shared with HSL, the acceptance of water-insoluble substrates, and the observed inhibition by classic lipase inhibitors, such as E600 (diethyl-*p*-nitrophenyl phosphate) [4, 5].

AADAC strongly contributes to drug hydrolysis, resulting in xenobiotic detoxification or prodrug activation [1]. The catalytic activity of AADAC has been tested on the hydrolysis and deacetylation on a wide variety of prodrugs, such as flutamide, phenacetin, indiplon, ketoconazole, rifabutin, rifampicin, and rifapentine [2, 6–9]. In contrast to other transmembrane enzymes, such as carboxylesterase 1 (CES1), carboxylesterase 2 (CES2), and paraoxonase 1 (PON1), AADAC selectively prefers bulky and small acyl moieties, such as fluorescein diacetate, *N*-monoacetyldapsone, and propanil [1]. Similarly, AADAC has been linked to the hydrolysis and activation of abiraterone acetate, a prodrug for metastatic castration-resistant prostate cancer [10].

Although in humans, AADAC is primarily expressed in the lumen side of the endoplasmic reticulum (ER) of the liver and gastrointestinal tract (jejunum, small intestine, and colon) [11, 12], its expression has also been found in the brain, particularly in the Purkinje cell layer of the human cerebellum, hippocampus, corpus callosum, and caudate nucleus [13]; adrenal cortex/medulla, and pancreas [14]. Due to its vast location, the physiological effects linked to an upper or lower expression or inhibition of AADAC could be beneficial, e.g., the upregulation of AADAC expression contributed to a better prognosis for several types of cancer [15–19]. On the other hand, variants or deletions of the AADAC gene have been linked to increased susceptibility to suffer Tourette syndrome [13].

In drug development, the extrapolation of data from orthologues is used to estimate the pharmacological effects and toxicity of drug candidates [20]. Orthologues are genes that evolved from a common ancestor by speciation; therefore, orthologue proteins are likely to have similar biological roles [21]. This similarity allows the identification of enzymes from organisms other than model organisms, such as birds, reptiles, or amphibians. Some AADAC orthologues, such as mouse, dog, rat, and cynomolgus macaque AADAC have been studied for their substrate recognition and specificity [11, 12, 22–24]. For example, human AADAC can hydrolyze flutamide, phenacetin, indiplon, and rifampicin, whereas rat and mouse AADAC only hydrolyzes flutamide and phenacetin, and not rifampicin [11]. In contrast, dog AADAC only hydrolyzes phenacetin and not indiplon [12]. Differences in enzyme location are also observed among orthologue AADAC. For instance, in human and cynomolgus macaques, AADAC mRNA is found in the liver and small intestine, whereas in rats and mice, mRNA AADAC is expressed in the kidney [22].

Mice and rats are the preferred organisms for toxicological studies, and differences in substrate preference by human, rat, and mouse liver microsomes expressing AADAC have also been observed [2]. Human and rat liver microsomes could hydrolyze indiplon, but mouse liver microsomes could not, whereas human and mouse liver microsomes hydrolyzed phenacetin and ketoconazole. Interestingly, neither mouse nor rat liver microsomes had any activity on rifamycins, whereas human liver microsomes showed activity values from 20 to 60 pmol/min/mg.

Given the importance of human AADAC in lipid, endobiotic, and xenobiotic metabolism, the present work focused on the functional analysis of AADAC orthologues, human AADAC-protein interactions, and physicochemical properties of a series of AADAC orthologue sequences from mammals, reptiles, amphibians, and birds.

## Methods

### Collection of AADAC protein sequences

AADAC amino acid sequences were retrieved from GenBank and Ensembl databases through the Basic Local Alignment Search Tool (BLAST). Searches were performed using the annotated Human Arylacetamide Deacetylase amino acid sequence (GenBank accession no. NP_001077.2, and Ensembl accession no. ENSG00000114771), and InterProScan was used to confirm the protein domains [25]. The orthologue sequences were selected according to the following parameters: (i) sequences with lengths between 393 and 415 amino acids; (ii) sequences without gaps, unspecific amino acids, or truncated regions, (iii) sequences containing the α/β hydrolase-3 domain, and (iv) sequences belonging to mammals, amphibians, birds, and reptiles.

### Phylogenetic analysis

The phylogenetic tree was constructed using an amino acid alignment of human AADAC orthologues with MUSCLE in Mega X Software (RRID:SCR_000667) [26] with the following settings: (i) hydrophobicity multiplier (1.20), (ii) max iterations (16), and (iii) cluster method (UPGMA), also, the distances were calculated using the Jones-Taylor-Thornton matrix (JTT), and the tree was created with the maximum likelihood method with 1000 bootstrap replicates. Finally, the Interactive Tree of Life (iTOL v6; https://itol.embl.de/) was employed to display the tree [27]. The Fitch (or similarity) matrix of the 142 orthologues was developed in RStudio software (v2022.07.2) using the R package SeqinR 1.0-2: Biological Sequences Retrieval and Analysis [28]. A protein alignment was used to calculate the pairwise distance matrix of aligned sequences using the function dist.alignment (x, matrix = similarity). The Numerical Taxonomy Multivariate Analysis System (NTSYSpc v2.02) software calculated the correlation matrix of amino acid sequences coding for the AADAC gene using the Dis/similarity module for qualitative data. Principal component analysis (PCA) was performed by plotting the 2-dimensional (2D) Dis/similarity matrix in NTSYSpc.

### AADAC-protein interaction prediction

Potential protein-protein association networks for human AADAC were predicted with the STRING online database (RRID:SCR_005223) [29], whereas the protein-protein association for AADAC orthologues was predicted with the BioGRID database (https://thebiogrid.org/) (RRID:SCR_007393) [30]. For the STRING database (https://string-db.org/), the sequences of AADAC were selected according to the above criteria. For the interaction analysis in BioGRID, the target-interacting protein data from human AADAC, paraoxonase 1 (PON1), carboxylesterase 1 (CES1), carboxylesterase 2 (CES2), carboxylesterase 3 (CES3) were used.

### Motif search

The lipase/esterase consensus motifs GXSXG and HGGG were inferred by sequence alignment with human AADAC and hormone-sensitive lipase (HSL, GenBank accession no. AAA69810.1). The logo plot representations were generated using Logomaker [31], and WebLogo [32]. The code that creates the Logomaker plots is available on GitHub at: https://github.com/jbkinney/logomaker/tree/master/logomaker.

To analyze the conserved motifs in AADAC orthologue proteins, 10 putative motifs between 6 and 50 amino acids were predicted with the MEME Suite 5.4.1 [33].

### Modeling of three-dimensional AADAC structures and predicted physicochemical properties and transmembrane regions

Protein modeling of AADAC was performed with AlphaFold via the Google Colab platform [34], and visualized with UCSF Chimera software [35]. The physicochemical parameters of human AADAC orthologues were predicted with ProtParam using the EXPASY server (RRID:SCR_018087) [36]. The sequences in FASTA were used to analyze the molecular weight (MW), theoretical *pI*, the total number of negatively and positively charged residues, amino acid composition, extinction coefficients, instability index, aliphatic index, and grand average of hydropathicity (GRAVY). In addition, the transmembrane regions were inferred with TMHMM 2.0 (RRID:SCR_014935) [37], and glycosylations were predicted with NetOGlyc 4.0 (RRID:SCR_009026) [38]. One-way non-parametric ANOVA was used to evaluate the significative differences between orthologues characteristics using GraphPad Prism version 8.0.2 (RRID:SCR_002798).

## Results

A total of 142 gene sequences from mammals (67), amphibians (4), birds (53), and reptiles (18) were collected from GenBank and confirmed with Ensembl database (Table 1) as orthologues. AADAC protein sequences showed ~ 48–100% identity across orthologues when human AADAC (NP_001077.2) was used as reference. In all cases, the α/β hydrolase domain was located between residues 107-376. Similarly, the amino acid residues of the catalytic triad of lipases/esterases (Ser, Asp/Glu, and His) were conserved among all the orthologues studied (Supplementary Figure S1).

**Table 1:** Structural characteristics from AADAC orthologues

Organism name	Taxonomy	Prediction method	Identity (%)	Motif			Active site (amino acid no.)			Carboxylesterase family	Transmembrane regions
				HGGG	GXSXG	Superfamily	S	D	H		
*Amblyraja radiata*	*Amphibian*	Genome	49.3	HGGG	GDSAG	Alpha/beta hydrolase 3	189	344	374	IV	1
*Geotrypetes seraphini*	*Amphibian*	Genome	55.7	HGGG	GDSAG	Alpha/beta hydrolase 3	189	344	373	IV	1
*Nanorana parkeri*	*Amphibian*	Genome	57.3	HGGG	GDSAG	Alpha/beta hydrolase 3	189	343	373	IV	0
*Rhinatrema bivittatum*	*Amphibian*	Genome	57.8	HGGG	GDSAG	Alpha/beta hydrolase 3	189	343	372	IV	0
*Anas platyrhynchos*	*Birds*	Genome	58.8	HGGG	GDSAG	Alpha/beta hydrolase 3	189	343	373	IV	0
*Anser cygnoides domesticus*	*Bird*	Genome	59.3	HGGG	GDSAG	Alpha/beta hydrolase 3	189	343	373	IV	0
*Antrostomus carolinensis*	*Bird*	Genome	58.3	HGGG	GDSAG	Alpha/beta hydrolase 3	189	343	373	IV	0
*Aptenodytes forsteri*	*Bird*	Genome	59.3	HGGG	GDSAG	Alpha/beta hydrolase 3	189	343	373	IV	1
*Apteryx australis mantelli*	*Bird*	Genome	64.5	HGGG	GDSAG	Alpha/beta hydrolase 3	189	343	373	IV	1
*Apteryx rowi*	*Bird*	Genome	64.5	HGGG	GDSAG	Alpha/beta hydrolase 3	189	343	373	IV	1
*Aquila chrysaetos chrysaetos*	*Bird*	Genome	59.8	HGGG	GDSAG	Alpha/beta hydrolase 3	189	343	373	IV	0
*Athene cunicularia*	*Bird*	Genome	59.8	HGGG	GDSAG	Alpha/beta hydrolase 3	189	342	373	IV	1
*Aythya fuligula*	*Bird*	Genome	59.3	HGGG	GDSAG	Alpha/beta hydrolase 3	189	343	373	IV	0
*Balearica regulorum gibbericeps*	*Bird*	Genome	59.3	HGGG	GDSAG	Alpha/beta hydrolase 3	189	343	373	IV	0
*Calidris pugnax*	*Bird*	Genome	62.5	HGGG	GDSAG	Alpha/beta hydrolase 3	189	343	373	IV	1
*Calypte anna*	*Bird*	Genome	58.3	HGGG	GDSAG	Alpha/beta hydrolase 3	189	343	373	IV	0
*Camarhynchus parvulus*	*Bird*	Genome	48	HGGG	GDSAG	Alpha/beta hydrolase 3	189	343	374	IV	0
*Catharus ustulatus*	*Bird*	Genome	53.3	HGGG	GDSAG	Alpha/beta hydrolase 3	189	343	373	IV	1
*Charadrius vociferus*	*Bird*	Genome	59.6	HGGG	GDSAG	Alpha/beta hydrolase 3	189	343	373	IV	0
*Chiroxiphia lanceolata*	*Bird*	Genome	57.6	HGGG	GDSAG	Alpha/beta hydrolase 3	189	343	373	IV	1
*Corapipo altera*	*Bird*	Genome	57.1	HGGG	GDSAG	Alpha/beta hydrolase 3	189	343	373	IV	1
*Corvus moneduloides*	*Bird*	Genome	54.6	HGGG	GDSAG	Alpha/beta hydrolase 3	189	343	373	IV	0
*Coturnix japonica*	*Bird*	Genome	57.3	HGGG	GDSAG	Alpha/beta hydrolase 3	189	343	373	IV	1
*Cuculus canorus*	*Bird*	Genome	61	HGGG	GDSAG	Alpha/beta hydrolase 3	189	343	373	IV	1
*Cyanistes caeruleus*	*Bird*	Genome	55.3	HGGG	GDSAG	Alpha/beta hydrolase 3	189	343	373	IV	0
*Cygnus atratus*	*Bird*	Genome	58.6	HGGG	GDSAG	Alpha/beta hydrolase 3	189	343	373	IV	0
*Egretta garzetta*	*Bird*	Genome	60.6	HGGG	GDSAG	Alpha/beta hydrolase 3	190	344	374	IV	2
*Empidonax traillii*	*Bird*	Genome	56.1	HGGG	GDSAG	Alpha/beta hydrolase 3	189	343	373	IV	0
*Eurypyga helias*	*Bird*	Genome	59.6	HGGG	GDSAG	Alpha/beta hydrolase 3	189	343	373	IV	0
*Falco cherrug*	*Bird*	Genome	60	HGGG	GDSAG	Alpha/beta hydrolase 3	189	343	373	IV	1
*Falco peregrinus*	*Bird*	Genome	60	HGGG	GDSAG	Alpha/beta hydrolase 3	189	343	373	IV	1
*Fulmarus glacialis*	*Bird*	Genome	58.6	HGGG	GDSAG	Alpha/beta hydrolase 3	189	343	373	IV	0
*Gallus gallus*	*Bird*	Genome	58.8	HGGG	GDSAG	Alpha/beta hydrolase 3	189	343	373	IV	1
*Gavia stellata*	*Bird*	Genome	60	HGGG	GDSAG	Alpha/beta hydrolase 3	189	343	373	IV	0
*Haliaeetus albicilla*	*Bird*	Genome	59.3	HGGG	GDSAG	Alpha/beta hydrolase 3	189	343	373	IV	0
*Haliaeetus leucocephalus*	*Bird*	Genome	59.3	HGGG	GDSAG	Alpha/beta hydrolase 3	189	343	373	IV	0
*Lepidothrix coronata*	*Bird*	Genome	57.3	HGGG	GDSAG	Alpha/beta hydrolase 3	189	343	373	IV	0
*Lonchura striata domestica*	*Bird*	Genome	52.4	HGGG	GDSAG	Alpha/beta hydrolase 3	189	343	373	IV	0
*Manacus vitellinus*	*Bird*	Genome	54.8	HGGG	GDSAG	Alpha/beta hydrolase 3	189	344	375	IV	0
*Meleagris gallopavo*	*Bird*	Genome	56.8	HGGG	GDSAG	Alpha/beta hydrolase 3	189	343	373	IV	1
*Melopsittacus undulatus*	*Bird*	Genome	60.8	HGGG	GDSAG	Alpha/beta hydrolase 3	189	343	373	IV	0
*Mesitornis unicolor*	*Bird*	Genome	58.6	HGGG	GDSAG	Alpha/beta hydrolase 3	189	343	373	IV	1
*Molothrus ater*	*Bird*	Genome	51.4	HGGG	GDSAG	Alpha/beta hydrolase 3	189	343	373	IV	0
*Neopelma chrysocephalum*	*Bird*	Genome	55.6	HGGG	GDSAG	Alpha/beta hydrolase 3	189	343	373	IV	0
*Nestor notabilis*	*Bird*	Genome	60.8	HGGG	GDSAG	Alpha/beta hydrolase 3	189	343	373	IV	1
*Numida meleagris*	*Bird*	Genome	58.8	HGGG	GDSAG	Alpha/beta hydrolase 3	189	343	373	IV	1
*Opisthocomus hoazin*	*Bird*	Genome	59.6	HGGG	GDSAG	Alpha/beta hydrolase 3	189	343	373	IV	1
*Parus major*	*Bird*	Genome	54.3	HGGG	GDSAG	Alpha/beta hydrolase 3	189	343	373	IV	0
*Phalacrocorax carbo*	*Bird*	Genome	60.8	HGGG	GDSAG	Alpha/beta hydrolase 3	189	343	373	IV	1
*Phasianus colchicus*	*Bird*	Genome	56.8	HGGG	GDSAG	Alpha/beta hydrolase 3	189	343	373	IV	1
*Pipra filicauda*	*Bird*	Genome	56.3	HGGG	GDSAG	Alpha/beta hydrolase 3	189	343	373	IV	1
*Pseudopodoces humilis*	*Bird*	Genome	55.3	HGGG	GDSAG	Alpha/beta hydrolase 3	189	343	373	IV	0
*Pygoscelis adeliae*	*Bird*	Genome	59.3	HGGG	GDSAG	Alpha/beta hydrolase 3	189	343	373	IV	1
*Strigops habroptila*	*Bird*	Genome	60	HGGG	GDSAG	Alpha/beta hydrolase 3	189	343	373	IV	0
*Struthio camelus australis*	*Bird*	Genome	63.5	HGGG	GDSAG	Alpha/beta hydrolase 3	189	343	373	IV	1
*Taeniopygia guttata*	*Bird*	Genome	52.9	HGGG	GDSAG	Alpha/beta hydrolase 3	189	343	373	IV	0
*Tyto alba alba*	*Bird*	Genome	59.6	HGGG	GDSAG	Alpha/beta hydrolase 3	189	343	373	IV	1
*Ailuropoda melanoleuca*	*Mammal*	Genome	74.7	HGGG	GDSAG	Alpha/beta hydrolase 3	190	344	374	IV	3
*Aotus nancymaae*	*Mammal*	Genome	88.5	HGGG	GDSAG	Alpha/beta hydrolase 3	189	343	373	IV	1
*Arvicanthis niloticus*	*Mammal*	Genome	68.9	HGGG	GDSAG	Alpha/beta hydrolase 3	188	342	372	IV	0
*Camelus bactrianus*	*Mammal*	Genome	74.4	HGGG	GDSAG	Alpha/beta hydrolase 3	189	343	373	IV	0
*Camelus dromedarius*	*Mammal*	Genome	74.4	HGGG	GDSAG	Alpha/beta hydrolase 3	189	343	373	IV	0
*Camelus ferus*	*Mammal*	Genome	74.4	HGGG	GDSAG	Alpha/beta hydrolase 3	189	343	373	IV	0
*Canis lupus dingo*	*Mammal*	Genome	75.4	HGGG	GDSAG	Alpha/beta hydrolase 3	189	343	373	IV	3
*Canis lupus familiaris*	*Mammal*	Genome	75.4	HGGG	GDSAG	Alpha/beta hydrolase 3	189	343	373	IV	3
*Castor canadensis*	*Mammal*	Genome	74.4	HGGG	GDSAG	Alpha/beta hydrolase 3	189	343	373	IV	3
*Chlorocebus sabaeus*	*Mammal*	Genome	93	HGGG	GDSAG	Alpha/beta hydrolase 3	189	343	373	IV	1
*Chrysochloris asiatica*	*Mammal*	Genome	83.2	HGGG	GGSAG	Alpha/beta hydrolase 3	189	343	373	IV	1
*Colobus angolensis palliatus - revisar*	*Mammal*	Genome	94	HGGG	GDSAG	Alpha/beta hydrolase 3	189	346	376	IV	1
*Condylura cristata*	*Mammal*	Genome	79.8	HGGG	GDSAG	Alpha/beta hydrolase 3	189	344	374	IV	1
*Cricetulus griseus*	*Mammal*	Genome	67.7	HGGG	GDSAG	Alpha/beta hydrolase 3	188	342	372	IV	1
*Desmodus rotundus*	*Mammal*	Genome	75.9	HGGG	GDSAG	Alpha/beta hydrolase 3	189	343	373	IV	0
*Dipodomys ordii*	*Mammal*	Genome	66.4	HGGG	GDSAG	Alpha/beta hydrolase 3	189	343	373	IV	3
*Elephantulus edwardii*	*Mammal*	Genome	74.2	HGGG	GDSAG	Alpha/beta hydrolase 3	189	343	373	IV	1
*Equus asinus*	*Mammal*	Genome	83.2	HGGG	GDSAG	Alpha/beta hydrolase 3	189	343	373	IV	1
*Erinaceus europaeus*	*Mammal*	Genome	75.2	HGGG	GDSAG	Alpha/beta hydrolase 3	189	343	373	IV	0
*Galeopterus variegatus*	*Mammal*	Genome	77.9	HGGG	GDSAG	Alpha/beta hydrolase 3	189	343	373	IV	2
*Gorilla gorilla gorilla*	*Mammal*	Genome	99	HGGG	GDSAG	Alpha/beta hydrolase 3	189	343	373	IV	1
*Grammomys surdaster*	*Mammal*	Genome	68.4	HGGG	GDSAG	Alpha/beta hydrolase 3	188	342	372	IV	1
*Halichoerus grypus*	*Mammal*	Genome	76.4	HGGG	GESAG	Alpha/beta hydrolase 3	189	343	373	VII	3
*Homo sapiens*	*Mammal*	Validated	100	HGGG	GDSAG	Alpha/beta hydrolase 3	189	343	373	IV	1
*Hylobates moloch*	*Mammal*	Genome	96.7	HGGG	GDSAG	Alpha/beta hydrolase 3	189	343	373	IV	1
*Leptonychotes weddellii*	*Mammal*	Genome	74.2	HGGG	GESAG	Alpha/beta hydrolase 3	189	343	373	VII	3
*Loxodonta africana*	*Mammal*	Genome	82.2	HGGG	GDSAG	Alpha/beta hydrolase 3	189	343	373	IV	1
*Macaca mulatta*	*Mammal*	Genome	92.7	HGGG	GDSAG	Alpha/beta hydrolase 3	189	343	373	IV	1
*Mandrillus leucophaeus*	*Mammal*	Genome	93.5	HGGG	GDSAG	Alpha/beta hydrolase 3	189	343	373	IV	1
*Marmota flaviventris*	*Mammal*	Genome	68.2	HGGG	GDSAG	Alpha/beta hydrolase 3	183	337	366	IV	1
*Mastomys coucha*	*Mammal*	Genome	67.4	HGGG	GDSAG	Alpha/beta hydrolase 3	187	342	372	IV	3
*Meriones unguiculatus*	*Mammal*	Genome	70.2	HGGG	GDSAG	Alpha/beta hydrolase 3	187	342	372	IV	0
*Mesocricetus auratus*	*Mammal*	Genome	66.9	HGGG	GDSAG	Alpha/beta hydrolase 3	188	342	372	IV	3
*Microcebus murinus*	*Mammal*	Genome	77.2	HGGG	GDSAG	Alpha/beta hydrolase 3	189	343	373	IV	1
*Microtus ochrogaster*	*Mammal*	Genome	67.8	HGGG	GDSAG	Alpha/beta hydrolase 3	190	344	374	IV	0
*Miniopterus natalensis*	*Mammal*	Genome	77.4	HGGG	GSSSG	Alpha/beta hydrolase 3	189	343	373	–	1
*Mus caroli*	*Mammal*	Genome	68.7	HGGG	GDSAG	Alpha/beta hydrolase 3	188	342	372	IV	1
*Mus musculus*	*Mammal*	Validated	69.9	HGGG	GDSAG	Alpha/beta hydrolase 3	188	342	372	IV	0
*Mus pahari*	*Mammal*	Genome	69.2	HGGG	GDSSG	Alpha/beta hydrolase 3	188	342	372	–	2
*Myotis davidii*	*Mammal*	Genome	74.7	HGGG	GDSAG	Alpha/beta hydrolase 3	189	343	373	IV	3
*Myotis lucifugus*	*Mammal*	Genome	75.4	HGGG	GDSAG	Alpha/beta hydrolase 3	189	343	373	IV	2
*Myotis myotis*	*Mammal*	Genome	74.9	HGGG	GDSAG	Alpha/beta hydrolase 3	189	343	373	IV	3
*Nannospalax galili*	*Mammal*	Genome	72.4	HGGG	GDSAG	Alpha/beta hydrolase 3	189	343	373	IV	0
*Nomascus leucogenys*	*Mammal*	Genome	96.7	HGGG	GDSAG	Alpha/beta hydrolase 3	189	343	373	IV	1
*Onychomys torridus*	*Mammal*	Genome	68.2	HGGG	GDSAG	Alpha/beta hydrolase 3	189	343	373	IV	1
*Ornithorhynchus anatinus*	*Mammal*	Genome	67.2	HGGG	GDSAG	Alpha/beta hydrolase 3	189	343	373	IV	0
*Pan paniscus*	*Mammal*	Genome	99.5	HGGG	GDSAG	Alpha/beta hydrolase 3	189	343	373	IV	1
*Pan troglodytes*	*Mammal*	Genome	99.2	HGGG	GDSAG	Alpha/beta hydrolase 3	189	343	373	IV	1
*Papio anubis*	*Mammal*	Genome	93.7	HGGG	GDSAG	Alpha/beta hydrolase 3	189	343	373	IV	1
*Peromyscus leucopus*	*Mammal*	Genome	68.4	HGGG	GDSAG	Alpha/beta hydrolase 3	188	342	372	IV	1
*Peromyscus maniculatus bairdii*	*Mammal*	Genome	68.2	HGGG	GDSAG	Alpha/beta hydrolase 3	188	342	372	IV	1
*Phoca vitulina*	*Mammal*	Genome	76.2	HGGG	GESAG	Alpha/beta hydrolase 3	189	343	373	VII	3
*Piliocolobus tephrosceles*	*Mammal*	Genome	93.7	HGGG	GDSAG	Alpha/beta hydrolase 3	189	343	373	IV	1
*Pipistrellus kuhlii*	*Mammal*	Genome	74.4	HGGG	GDSAG	Alpha/beta hydrolase 3	189	343	373	IV	2
*Pteropus alecto*	*Mammal*	Genome	69.9	HGGG	GSSSG	Alpha/beta hydrolase 3	189	342	372	–	1
*Rattus norvegicus*	*Mammal*	Transcriptome	67.7	HGGG	GDSAG	Alpha/beta hydrolase 3	188	342	372	IV	3
*Rattus rattus*	*Mammal*	Genome	67.7	HGGG	GDSAG	Alpha/beta hydrolase 3	188	342	372	IV	3
*Rhinopithecus bieti*	*Mammal*	Genome	92.2	HGGG	GDSAG	Alpha/beta hydrolase 3	189	343	372	IV	1
*Rhinopithecus roxellana*	*Mammal*	Genome	92.2	HGGG	GDSAG	Alpha/beta hydrolase 3	189	343	373	IV	1
*Saimiri boliviensis boliviensis*	*Mammal*	Genome	89.5	HGGG	GDSAG	Alpha/beta hydrolase 3	189	343	373	IV	1
*Sapajus apella*	*Mammal*	Genome	89.2	HGGG	GDSAG	Alpha/beta hydrolase 3	189	343	373	IV	1
*Talpa occidentalis*	*Mammal*	Genome	82	HGGG	GDSAG	Alpha/beta hydrolase 3	189	343	373	IV	1
*Theropithecus gelada*	*Mammal*	Genome	93.5	HGGG	GDSAG	Alpha/beta hydrolase 3	189	343	373	IV	1
*Trachypithecus francoisi*	*Mammal*	Genome	92.2	HGGG	GDSAG	Alpha/beta hydrolase 3	189	343	373	IV	1
*Urocitellus parryii*	*Mammal*	Genome	69.4	HGGG	GDSAG	Alpha/beta hydrolase 3	183	367	337	IV	3
*Vicugna pacos*	*Mammal*	Genome	75.2	HGGG	GDSAG	Alpha/beta hydrolase 3	189	343	373	IV	0
*Vulpes vulpes*	*Mammal*	Genome	76.7	HGGG	GDSAG	Alpha/beta hydrolase 3	189	343	373	IV	3
*Alligator sinensis*	*Reptiles*	Genome	62.0	HGGG	GDSSG	Alpha/beta hydrolase 3	189	343	373	–	1
*Anolis carolinensis*	*Reptiles*	Genome	61.3	HGGG	GDSAG	Alpha/beta hydrolase 3	189	343	373	IV	0
*Chelonoidis abingdonii*	*Reptiles*	Genome	63.3	HGGG	GDSAG	Alpha/beta hydrolase 3	189	343	373	IV	0
*Crocodylus porosus*	*Reptiles*	Genome	63	HGGG	GDSSG	Alpha/beta hydrolase 3	189	343	373	IV	0
*Gavialis gangeticus*	*Reptiles*	Genome	63	HGGG	GDSSG	Alpha/beta hydrolase 3	189	343	373	–	1
*Gekko japonicus*	*Reptiles*	Genome	60.1	HGGG	GDSAG	Alpha/beta hydrolase 3	190	344	374	IV	0
*Lacerta agilis*	*Reptiles*	Genome	60.5	HGGG	GDSAG	Alpha/beta hydrolase 3	189	343	372	IV	0
*Notechis scutatus*	*Reptiles*	Genome	58.1	HGGG	GDSAG	Alpha/beta hydrolase 3	189	343	373	IV	1
*Pantherophis guttatus*	*Reptiles*	Genome	58.6	HGGG	GDSAG	Alpha/beta hydrolase 3	189	343	373	IV	0
*Pelodiscus sinensis*	*Reptiles*	Genome	60.8	HGGG	GDSAG	Alpha/beta hydrolase 3	199	353	383	IV	0
*Podarcis muralis*	*Reptiles*	Genome	62.3	HGGG	GDSAG	Alpha/beta hydrolase 3	189	343	372	IV	0
*Pogona vitticeps*	*Reptiles*	Genome	58.8	HGGG	GDSAG	Alpha/beta hydrolase 3	189	343	373	IV	0
*Protobothrops mucrosquamatus*	*Reptiles*	Genome	58.1	HGGG	GDSAG	Alpha/beta hydrolase 3	189	343	373	IV	0
*Pseudonaja textilis*	*Reptiles*	Genome	58.8	HGGG	GDSAG	Alpha/beta hydrolase 3	189	343	373	IV	0
*Python bivittatus*	*Reptiles*	Genome	59.1	HGGG	GDSAG	Alpha/beta hydrolase 3	189	343	373	IV	1
*Thamnophis sirtalis*	*Reptiles*	Genome	58.3	HGGG	GDSAG	Alpha/beta hydrolase 3	189	343	373	IV	0
*Trachemys scripta elegans*	*Reptiles*	Genome	63.3	HGGG	GDSAG	Alpha/beta hydrolase 3	189	343	373	IV	0
*Zootoca vivipara*	*Reptiles*	Genome	61.3	HGGG	GDSAG	Alpha/beta hydrolase 3	189	343	372	IV	0

**Fig. 1:**
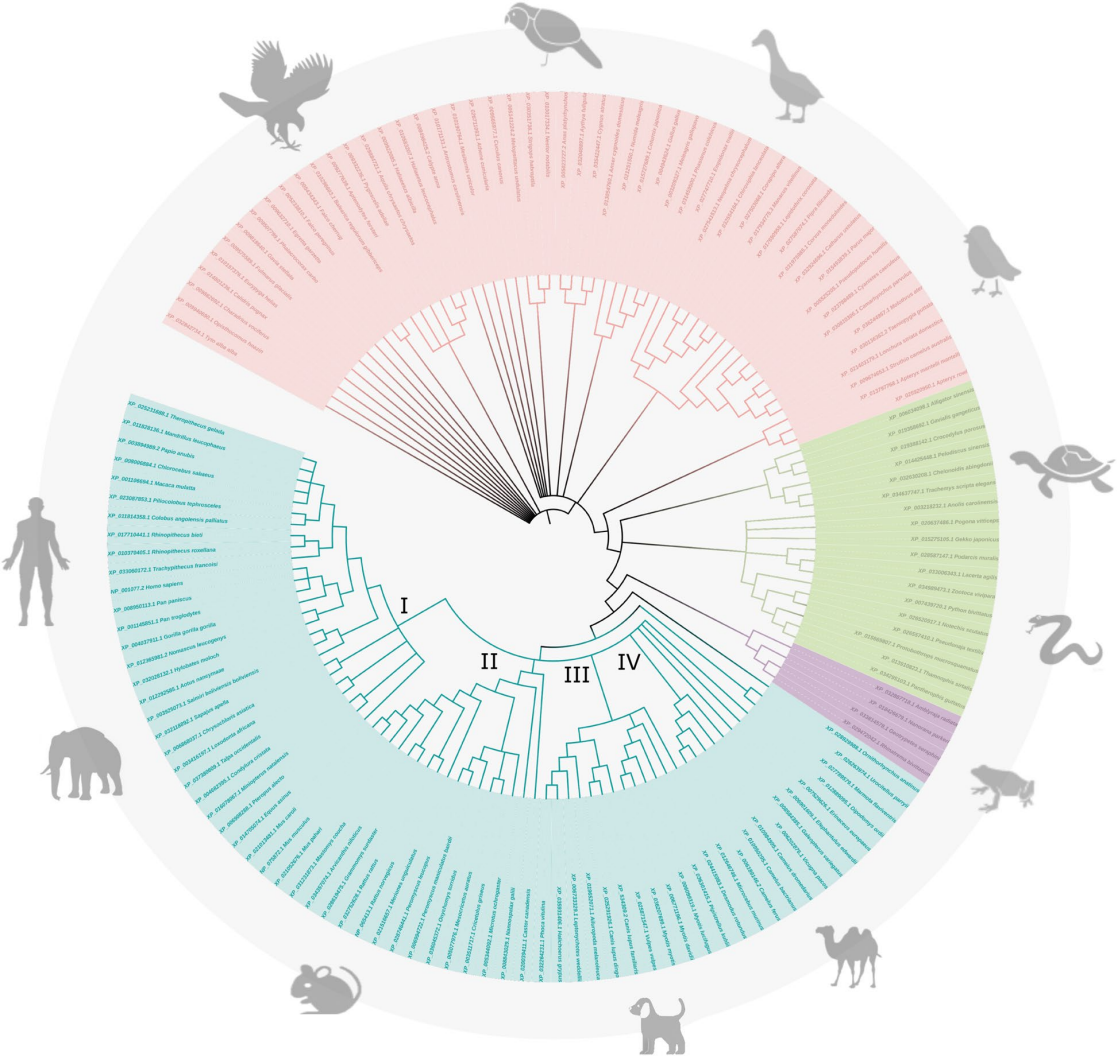
Maximum Likelihood tree showing the phylogenetic relationships of AADAC orthologues. Mammal AADAC sequences are distributed in clades. Sequences from mammals are highlighted in blue, amphibians in lilac, reptiles in green, and birds in pink. Distances were calculated using the Jones-Taylor-Thornton matrix (JTT), and the tree was created with the Maximum Likelihood method with 1,000 bootstrap replicates

**Fig. 2:**
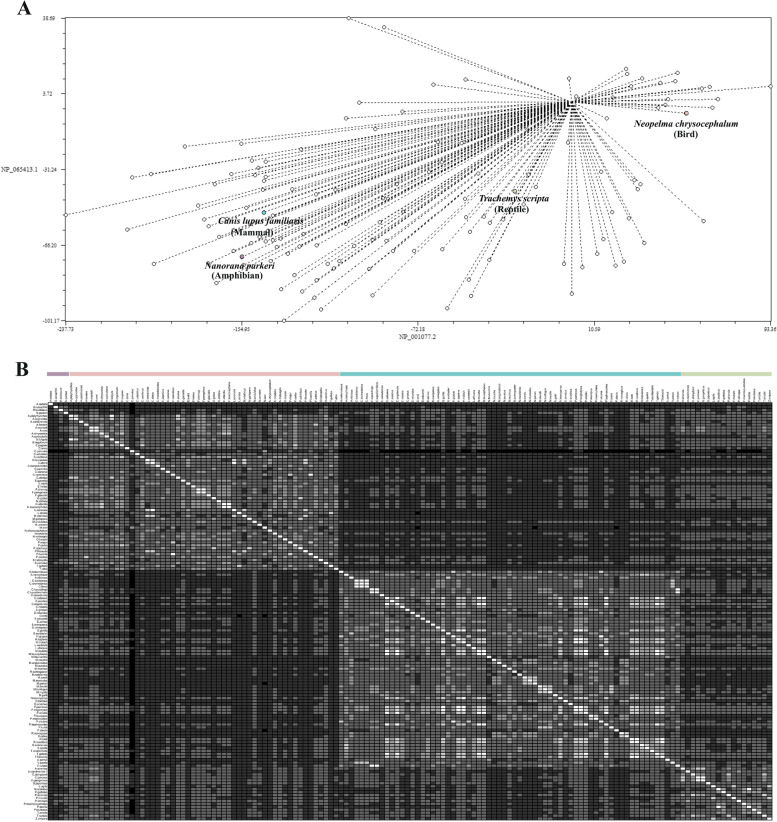
Principal component analysis. **A** The Fitch matrix of the 142 orthologues. **B** Correlation matrix of the amino acid sequences coding for the AADAC genes, the Dis/similarity matrix was created in NTSYSpc; amphibian organisms are represented in purple, avian organisms are represented in pink, mammalian organisms are represented in blue, and reptiles in green

The phylogenetic relationships of AADAC orthologues are shown in Fig. 1. The avian taxon had a common ancestor from which descended two distinctive subgroups: (i) clades XII and XIII, and (ii) clades VIII to XI. Under these terms, it is inferred that birds have the lowest percentage of identity with human AADAC. The analysis shows a marked difference between AADAC from the selected organisms. In mammals, four distinctive clades were observed: in clade I; human AADAC shared a common ancestor with AADAC from *Pan paniscus* and *Pan troglodytes*; in clade II, mouse and rat AADAC were grouped; clade III belonged to dog AADAC, whereas camels and hedgehogs were grouped in clade IV. Amphibians, represented by clade V, and clade IV from mammals evolved from a common ancestor. Reptiles, divided into clades VI and VII, share a common ancestor. PCA shows the similarity between orthologs of different taxa (Fig. 2A, B). It can be observed that organisms of the same taxon share more significant similarities (Fig. 2B), comparable to that observed in the phylogenetic tree (Fig. 1).

**Fig. 3:**
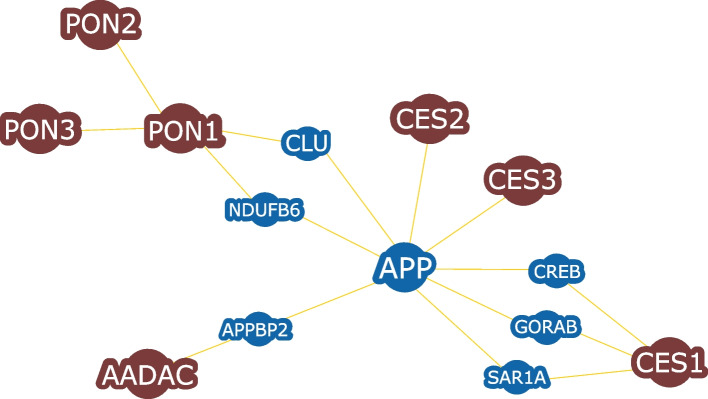
BioGRID-base human AADAC-protein interaction network

Human AADAC is closely related to three hydrolytic enzymes belonging to the carboxylesterase family: CES1, CES2, and CES3, which are ER-linked enzymes that contain the α/β hydrolase domain and are primarily known to be involved in phase I of drug or xenobiotic metabolism [39] . A single interactome to relate the genetic and physical interactions of CES1, CES2, PON1, and human AADAC proteins/genes was constructed (Fig. 3). The amyloid-beta precursor protein (APP) shows a chemical-protein interaction with CES2, AADAC, CES1, and PON1 (Fig. 3).

In humans, APP is a cell surface protein with signal transduction properties that control the viability, proliferation, migration, and aggressiveness of various cancer cells, such as breast cancer, ovarian cell adenocarcinoma, medulloblastoma, and neuroblastoma [32]. Additionally, APP interacted with Amyloid-Beta Precursor Protein Binding Protein 2 (APPBP2), which is involved in non-small cell lung cancer (NSCLC), a type of cancer accountable for ~ 80% of all lung carcinoma patients [33]. On the other hand, the interactions of 70 AADAC orthologues were analyzed using the STRING database (Supplementary Table S2). From the sequences studied, 45 AADAC orthologues were related to dihydrodiol dehydrogenases (DHDH), 57 sequences showed an interaction with the succinate receptor 1 (SUCNR1), and 44 interacted with ATP8B3. DHDH is involved in the detoxication of trans-dihydrodiol and carcinogenic metabolites of polycyclic aromatic hydrocarbons [40]; SUCNR1 is related to conditions such as hypertension, diabetes, and obesity [41], whereas ATP8B3 is involved in aminophospholipid transport in the liver [42].

**Fig. 4:**
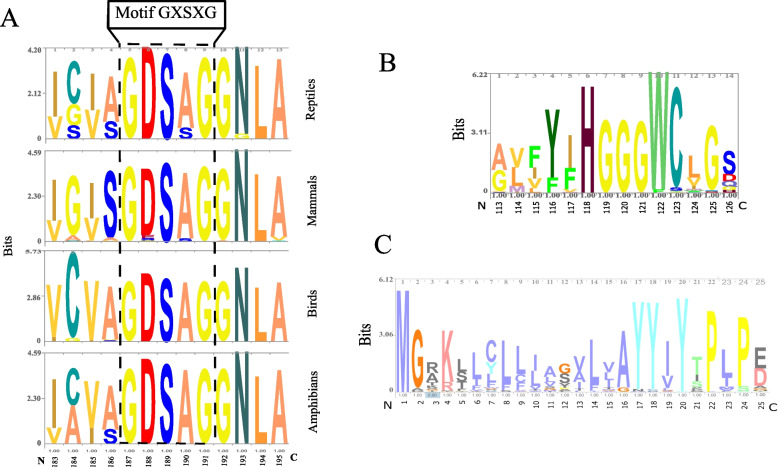
Conserved AADAC motifs using LogoMaker (A) and WebLogo (B and C). In all cases, the amino acid number indicated in the image is relative to the human AADAC sequence. **A** Logo showing the conserved GXSXG motif, which depicts the catalytic Ser, located in residues 113 to 126. **B** Logo showing the conserved HGGG motif, located in residues 113 to 126. **C** Logo showing the conserved transmembrane region of AADAC orthologues, located in residues 1 to 25

**Fig. 5:**
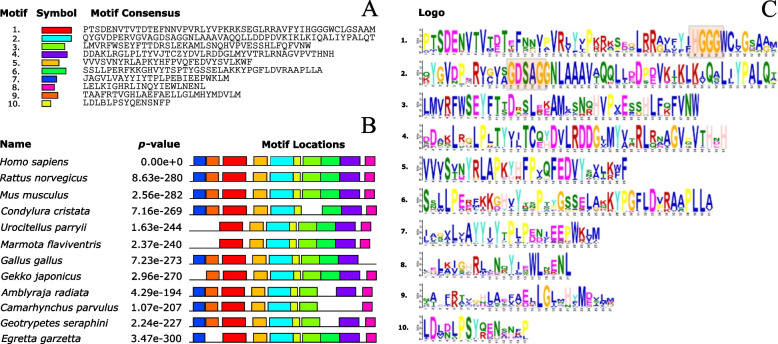
Conserved motifs in AADAC orthologues using MEME-suite. **A** Ten representative conserved motifs. Motif 1 (red), motif 2 (cyan), motif 3 (green), motif 4 (violet), motif 5 (orange), motif 6 (lime), motif 7 (blue), motif 8 (fuchsia), motif 9 (dark orange), and motif 10 (yellow). **B** The locations along the sequence of the 10 representative motifs depicted in **A**. The complete list is illustrated in Supplementary Figure S2. **C** Representation of all motifs of AADACs orthologues. The HGGG and GXSXG motifs are highlighted in gray. N and C, at the bottom of the images represent the amino terminal and carboxyl terminal

**Fig. 6:**
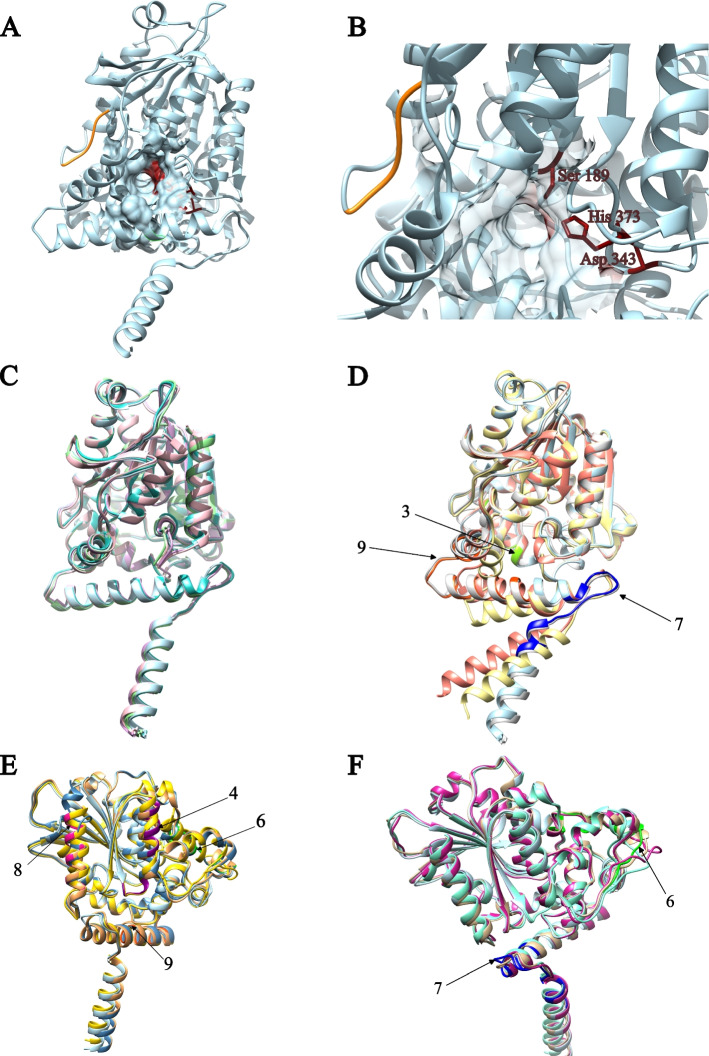
Tridimensional structural comparison of AADAC from orthologues. **A** ADDAC from *Homo sapiens*, the active site is shown in red, and the YXLXP motif in orange. **B** Active site of AADAC from *Homo sapiens*. The catalytic amino acids are represented by sticks. **C** Structural alignment of representative organisms from mammals (*Macaca mulata* in aqua), amphibian (*Nanorana parkeri* in pink), birds (*Anas platyrhynchos* in purple), reptiles (*Trachemys scripta elegans* in light green). **D** Structural alignment of mammalian AADAC showcasing the motif differences (*Condylura cristata* in grey color*, Urocitellus parryii* in salmon color and *Marmota flaviventris* in yellow color). **E** Structural alignment of birds (*Camarhynchus parvulus* in gold color, *Gallus gallus* in steel blue and Egretta garzetta in brown). **F** Structural alignment of amphibian and reptile organisms with motif differences (*Amblyraja radiata* in pink, *Geotrypetes seraphini* in aquamarine and *Gekko japonicus* in beige). The numbers indicate the structural motif related to MEME suite analysis (Fig. 3)

The structural motif of the consensus pentapeptide GXSXG (Table 1 and Fig. 4A) was illustrated using consensus sequence logos grouped by amphibians, birds, mammals, and reptiles. Four different residues were found occupying the X position of the GXSXG sequence: GDSAG (92%), GESAG (2.1%), GDSSG (2.8%), and GSSSG (1.4%), while the HGG[G/A] tetrapeptide block was conserved through all AADAC orthologues, being the G/Gly residue following the HGG motif (HGGG) extensively conserved (99.2%) (Fig. 4B and Fig. 5C/motif 1). The search for structural motifs using the MEME-suite (motif-based sequence analysis tools) displayed 10 essential motifs for all AADAC sequences studied. Motifs 1, 3, 6, 8, and 9 were absent in star-nosed mole (*Condylura cristata*), arctic ground squirrel (*Urocitellus parryii*), yellow-bellied marmot (*Marmota falviventris*), chicken (*Gallus gallus*), gecko (*Gekko japonicus*), maiden ray (*Amblyraja radiata*), small tree finch (*Camrahynchus parvulus*), gaboon caecilian (*Geotrypetes seraphini*), and little egret (*Egretta garzetta*) (Fig. 5 and Supplementary Figure S2). Motif 7 (Fig. 5) corresponds to the transmembrane region, while motifs 1, 3, had glycosylation sites such as NVTV, NWSS, and NWSN. A possibly important region identified in motif 5 is the YXLXP sequence, where X represents the amino acids R/Arg or A/Ala present in all orthologues (Supplementary Figure S1, Fig. 5/motif 5), and forms a β-α loop in all orthologues sequences studied (Fig. 5A, B). The AADAC folding among organisms with 10 motifs was similar to human (Fig. 5C), and sequences with any missing motif showed structural differences, mainly in the formation of alpha helices and beta strands (Fig. 6D, F).

**Fig. 7:**
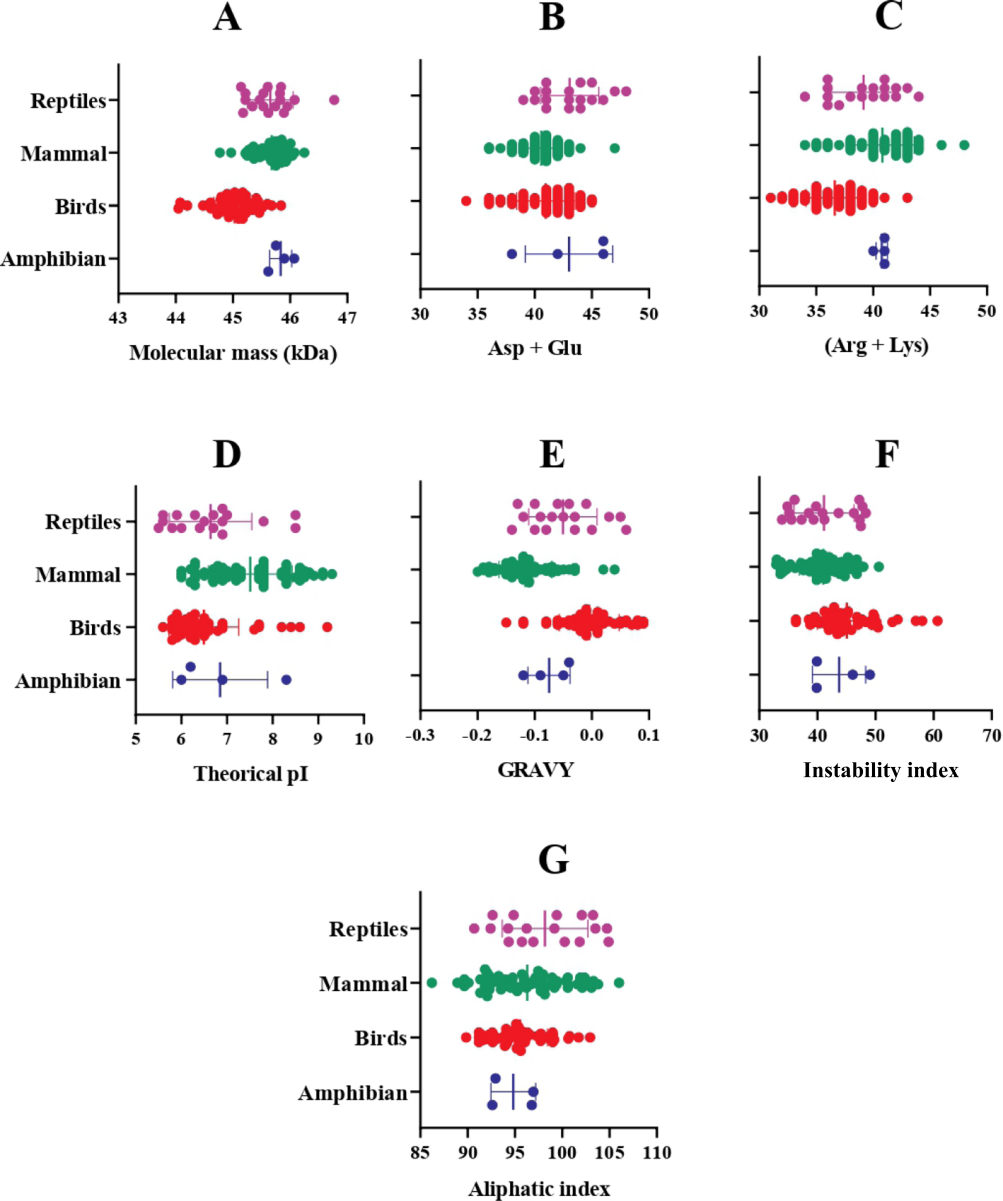
Distribution plot depicting seven major predicted physicochemical properties of AADAC orthologues using the ProtParam Tool. **A** Molecular mass (kDa), **B** Percentage of negatively charged amino acids (Asp + Glu), **C** Percentage of positively charged amino acids (Arg + Lys), **D** Theoretical isoelectric point (*pI*), **E** Grand average of hydropathicity index (GRAVY), **F** Instability index, and **G** Aliphatic index. The complete computed physicochemical properties for each AADAC ortholog are depicted in Supplementary Table S1. One-way non-parametric ANOVA was used to evaluate significant differences between orthologues characteristics using GraphPad Prism version 8.0.2

The physicochemical characterization shows that the molecular mass among orthologs remains between 44 and 46 kDa; however, for mammals, the mean is 45.8 kDa, while for birds and amphibians, it is 45.0 and 45.8 kDa, respectively. The number of negatively charged residues (Asp + Glu) was greater than the number of positively charged residues (Arg + Lys). The sum of hydropathy values of all amino acids divided by protein length showed similar tendencies between orthologues. The instability index is between 31 and 62 h, and some birds orthologues showed the most stable AADAC (Fig. 7). The prediction of *N*-glycosylation sites with NetNGlyc-4.0 server projected 4, 1, 6, and 4 different patterns for amphibians, bird, mammals, and reptiles, respectively.

The transmembrane regions of AADAC orthologues varied between 0 and 3. From 142 orthologue sequences, 48 organisms did not show transmembrane regions (33.8%), 73 organisms showed a single transmembrane region with the C-terminal oriented towards the lumen (51.4%), 5 organisms had 2 transmembrane regions with the active site towards the cytosol (3.5%), and 16 organisms showed 3 transmembrane regions with the C-terminal carboxyl oriented towards the ER lumen (11.2%) (Table 1).

## Discussion

The phylogenetic history of human AADAC orthologues infers those common ancestral sequences evolved in the avian taxon, with speciation events occurring in a descending fashion in reptile, amphibian, and mammalian taxa, respectively.

Only nine AADAC orthologues showed interactions with carboxyl esterases (CES), belonging to red fox (*Vulpes vulpes*), Bolivian squirrel monkey (*Samiri boliviensis*), brown rat (*Rattus norvegicus*), house mouse (*Mus musculus*), *Mus pahari*, shrew-like mouse (*Mus coroli*), Mongolian gerbil (*Meriones unguiculatus*), one-humped camel (*Camelus dromedarius*), and human (*Homo sapiens*). It can be inferred that these nine AADAC may also be able to hydrolyze human AADAC substrates, such as xenobiotics.

The consensus sequence GDSAG found in mammals, birds, reptiles, and amphibians belongs to family IV of α/β hydrolases, also known as HSL family. The GESAG motif, found only in three orthologues, gray seal (*Halichoerus grypus*), common seal (*Phoca vitulina*), and weddell seal (*Leptonychotes weddellii*)), belonged to family VII of α/β hydrolases [43]. Three AADAC orthologues belonging to the order of Crocodilia (*Alligator sinensis*, *Crocodylus porosus*, and *Gavialis gangeticus*) and shrewmouse (*Mus pahari*) showed the GDSSG consensus sequence. The natal long-fingered bat (*Miniopterus natalensis*) and the black flying fox (*Pteropus alecto*) were the only AADAC orthologues showing the consensus pentapeptide GSSSG (Table 1 and Supplementary Figure S1).

The HGGG motif forms a loop close to the active site and directly participates in the catalytic hydrolysis by stabilizing the oxyanion intermediate of the reaction. The presence of multiple G/Gly residues confers greater flexibility to this loop. In contrast, some studies point that a change in the last G/Gly residue for an A/Ala decreases the enzyme activity up to 40% [44]. *Pteropus alecto* AADAC was the only orthologue to show an A/Ala residue placed at the last position (HGGA). The YXLXP motif has been previously reported in the telomeric protein SNM1B/Apollo (a member of the Metallo-β-lactamase/β-CASP family), which is involved in binding to TRF2 (telomeric repeat-binding factor 2) [45]; Similarly, SLX4 protein involved in DNA repair, contains a H/YXLXP motif and binds human TRF2. A similar site in the TRFH domain of TRF1 (around residue F142), FXLXP motif, serves as an anchor to TIN2 protein (TRF1-interacting protein 2) [46, 47].

Another essential structural region is the transmembrane region (Fig. 3C). The amino acids belonging to this region are responsible for anchoring the protein to the lipid bilayer. The most conserved amino acids among the studied orthologues were Y/Tyr, P/Pro, G/Gly, and K/Lys. In contrast, amino acids at positions 3, 5, 11, 12, 15, 21, and 23 showed increased variability among AADAC orthologues.

The predicted physicochemical characteristics of AADAC orthologues are resumed in the Supplementary Table S1. The calculated Molecular weight (MW) of AADAC orthologues is shown for amphibians, birds, mammals, and reptiles, and the minimum and maximum values are highlighted using a box and whisker plot (Fig. 7A). Human AADAC has been previously purified and characterized from liver tissue. Its MW was deduced at 45.671 kDa by gel electrophoresis [4], which is consistent with the MW value obtained by ProtParam for human AADAC (45.73 kDa), and similar to the average MW value calculated for mammals (45.68 kDa). The highest MW value belonged to AADAC from reptiles (46.77 kDa), while AADAC from birds had an average of 45.1 kDa, being the taxa with the lowest MW values.

The amount of Asp + Glu and Arg + Lys is related to the isoelectric point of the protein (Fig. 5B, C). Human AADAC orthologues showed isoelectric point (*pI*) values between 5.5 and 9.3 (Fig. 5D and Supplementary Table S1). The theoretical *pI* of human AADAC was 8.75, which differed from the *pI* calculated by in vivo experiments (9.36) [4]. Mammals showed *pI* values ranging from 6.0 to 9.3, followed by birds (5.6–9.2), reptiles (5.5–8.5), and amphibians (6.0–8.3).

The GRAVY value for a protein or a peptide is the sum of hydropathy values of all amino acids divided by the protein length [48]. Positive GRAVY values indicate hydrophobicity, whereas negative values mean hydrophilicity. The GRAVY value for human AADAC was found to be negative, implying a hydrophilic character, and similar GRAVY results were observed for mammals, amphibians, and reptiles AADAC (Fig. 7E). In contrast, birds were the only taxa showing positive GRAVY values, thus indicating hydrophobicity.

The instability index of a protein is calculated based on the presence of certain dipeptides. If the instability index value of a given protein is less than 40, it is considered stable under atmospheric conditions, and have a longer in vivo life span (~ 16 h), whereas proteins with an instability index > 40 show an in vivo half-life of less than 5 h [49]. The instability index value of human AADAC was 33.96 (Fig. 7E), which is considered a stable protein for in vivo experiments. In fact, human AADAC has already been expressed in sf9, DH10Bac, and *E. coli* cells, further purified and characterized for drug hydrolysis assays [6, 50, 51]. The average instability index values of AADAC orthologues ranged from 40 to 45 (Fig. 7F). Different model organisms for toxicological studies found in the mammalian taxon, such as mice, rats, and dogs, showed a protein instability index > 40, thus classifying them as in vivo unstable with a life span of fewer than five hours. Despite these values, mouse, rat, and dog AADAC microsomes have been successfully used in vitro to hydrolyze various prodrugs, confirming their role in the metabolism of the already known substrates of human AADAC [11, 12, 52].

For human AADAC, the high aliphatic index value was 99.15, which is considered thermostable. In general, all the AADAC orthologues studied showed aliphatic index values above 86.22, indicating a reliable degree of thermostability (Fig. 7G).

The potential *N*-glycosylation sites of amphibian, bird, mammal, and reptile AADAC were predicted with the NetNGlyc-4.0 server, and the results are detailed in Table 1. For *N*-glycosylation, an *N*-acetylglucosamine residue is linked via an amide bond to an asparagine (N/Asn) from the consensus sequence NX(S/T), where X can be any amino acid except proline (P/Pro). The presence of the consensus sequence is required for *N*-linked glycosylation; however, the potential sites might not be glycosylated [53]. For amphibians, the neuronal prediction gave two main glycosylation sites. All four amphibian sequences showed glycosylation sites at 78(NVTV/I). For bird AADAC, 39 sequences had one glycosylation site (78(NVTV), 73.6% of sequences), 12 sequences had two glycosylation sites (78(NVTV)-282(NWS[Q/R/V/L/N]) or 78(NVTV)-88(N[V/T/I][S/T]V), 22.6% of sequences), whereas the sequence of the bird *Neopelma chrysocephalum* showed no glycosylation site.

Mammalian AADAC showed six different glycosylation patterns. The most common pattern was 78(NV/ITV)-282(NWSS/A) (36 sequences in total, 53.7%), including AADAC sequences of *Homo sapiens*, *Pan paniscus*, *Pan troglodytes*, and *Canis lupus familiaris*. This is in agreement with the results reported in the literature [52], where their findings proved that *N*-glycosylation at the N282 residue of human AADAC was crucial for enzymatic activity by proper folding. The following observed patterns were 77(NVTV) (7 sequences, 10.4%, including *Rattus rattus* and *Rattus norvegicus*), and 282(NWSS) (5 sequences, 7.5%), and 77(NVTV)-281(NWSS) (4 sequences, 6%). Three glycosylation sites were found for four mammal sequences (78(NITV)-281(NWSS)-298(NRTY), 78(NVTV)-282(NWSS)-302(NGTS), 56(NHSM)-78(NVTV)-282(NWSS), and 55(NHSM)-77(NVTV)-281(NWSS), whereas 11 mammal sequences showed other glycosylation patterns. The AADAC sequence of *Mus musculus* had no *N*-glycosylation sites.

The prediction of *N*-glycosylation sites for reptile AADAC projected four different patterns. Six reptile sequences (33.3%) had the 282(NWS/N/R/H/K/E) site, 4 sequences (22.2%) had the 78(NVTV)-282(NWSN) site, 5 sequences (27.8%) showed different *N*-glycosylation patterns, and 3 sequences (16.7%) had no *N*-glycosylations.

In humans, AADAC is a type II transmembrane protein comprising a short, positively charged *N*-terminal region oriented towards the cytoplasm, a single transmembrane region, and a *C*-terminal tail oriented towards the lumen of the ER, containing the catalytic triad Ser189, Asp343, and His373 [5, 52, 54]. The lumen location of the catalytic triad was confirmed by the hydrolysis of D-13223, a substrate that is not observed in human liver cytosol [55].

The transmembrane regions of human AADAC and its orthologues were predicted with TMHMM (Table 1). TMHMM has a reliability of soluble and transmembrane proteins of 70–80% of assertiveness [37]. The server predicted a transmembrane helix located at residues 5–24 (Table 1), and an outer sequence of amino acid 25-399, which is consistent with the literature [54]. The active site of human AADAC faces the lumen site of the endoplasmic reticulum (RE), which implies different catalytic capacities compared to other carboxylesterases [5]. For example, substrates for cytosolic lipases, such as glycerolphospholipids or triglycerides stored in cytoplasmic lipid droplets, cannot cross the ER membrane and therefore are not hydrolyzed by AADAC [5].

All studied orthologues shared the same amino-terminal location at the cytosol, whereas the *C*-terminal was oriented towards the lumen of the RE.

## Conclusions

Although AADAC is a ubiquitous protein spread among organisms from different taxa, drug hydrolysis assays and toxicological studies are limited to a few species. The predicted physicochemical characteristics of the studied 142 orthologues show similarity among the groups; however, the composition of amino acids as Arg + Lys and Asp + Glu ratios differed among the studied organisms, which generates differences between the isoelectric point. The orthologues were related to *DHDH*, *ATP8B3*, and *P2RY1* genes, expressed in the lipid membrane, and directly related to skin and lung cancer genes (APP and APPBP2), suggesting that they may share mechanisms among these diseases. In addition, the comparative analysis of AADAC orthologues revealed that the sequence YXLXP, which has been involved as DNA repair mechanism, was shared by all the organisms studied. Other structural motifs that are fingerprints of lipolytic enzymes (GXSXG and HGGG) were found in all orthologs; however, according to the predicted 3D structures, missing motifs can generate conformational changes that can affect enzyme-substrate interaction.

The number of transmembrane regions varied among organisms and did not follow a pattern among mammals, amphibians, birds, or reptiles. Some substrate selectivities among the studied orthologues could be inferred by the phylogenetic tree constructed; however, further biochemical studies are mandatory to corroborate the results herein presented. This information, together with the analysis of the interactome, offers an overview of how these AADAC from the studied organisms could interact with various drugs tested mainly in primates or rodents. The collected data shown in this work broadens the knowledge of AADAC orthologues and could help to better select new model organisms as future candidates for detoxification, xenobiotic processing, and drug disposition studies.

## Supplementary Information

**Additional file 1: Figure S1.** Amino acid sequence alignments for AADAC orthologues. The name of the orthologues is shown as an accession number on the left side. "⁎" shows identical residues for AADAC orthologues; ": "2 alternate residues. On grey color, principal identical residues referent to HGGG box, Consensus sequence (GXSXG), and YXLXP motif. Loops above the amino acid sequence indicate alpha-helix structures, and arrows indicate beta-sheet structures. **Figure S2.** The complete motifs analysis, using MEME-suite of AADAC orthologues. **Table S1.** Summary of the physicochemical characterization, transmembrane regions, and N-glycosylation sites predicted for AADAC orthologue. **Table S2.** The complete protein-protein interaction network analysis of AADAC orthologues using the STRING tool.

## Data Availability

The manuscript has data included as electronic supplementary material.

## Abbreviations

AADAC: Arylacetamide deacetylase
HSL: Hormone-sensitive lipases
E600: Diethyl-p-nitrophenyl phosphate
CES1: Carboxylesterase 1
CES2: Carboxylesterase 2
PON1: Paraoxonase 1
BLAST: Basic Local Alignment Search Tool
MW: Molecular weight
