# Supplementary material for: Characterization, comparative, and functional analysis of arylacetamide deacetylase from Gnathostomata organisms
Source: J Genet Eng Biotechnol. 2022 Dec 21;20:169. doi: 10.1186/s43141-022-00443-z (PMC9772364; doi:10.1186/s43141-022-00443-z)
Supplement: Supplementary file 1 — Additional file 1: Figure S1. Amino acid sequence alignments for AADAC orthologues. The name of the orthologues is shown as an accession number on the left side. "⁎" shows identical residues for AADAC orthologues; ": "2 alternate residues. On grey color, principal identical residues referent to HGGG box, Consensus sequence (GXSXG), and YXLXP motif. Loops above the amino acid sequence indicate alpha-helix structures, and arrows indicate beta-sheet structures. Figure S2. The complete motifs analysis, using MEME-suite of AADAC orthologues. Table S1. Summary of the physicochemical characterization, transmembrane regions, and N-glycosylation sites predicted for AADAC orthologue. Table S2. The complete protein-protein interaction network analysis of AADAC orthologues using the STRING tool. [file 43141_2022_443_MOESM1_ESM.zip › Supplementary data JGEB-D-22-00196R1.docx]

Supplementary data

**Characterization, comparative and functional analysis of arylacetamide deacetylase from Gnathostomata organisms**

**Content:**

- **Supplementary Fig. 1**; 2-20 pp.
- **Supplementary Fig. 2**; 21 pp.
- **Supplementary Table 1**; 22-30 pp.

**Supplementary Fig. S1**. Amino acid sequence alignments for AADAC orthologues. “⁎” shows identical residues for AADAC orthologues; “: “ two alternate residues. On grey color, principal identical residues referent to HGGG box, Consensus sequence (GXSXG), and YXLXP motif. Loops above the amino acid sequence indicate alpha-helix structures, and arrows indicate beta-sheet structure. The name of orthologues is shown as an accession number on the left side.


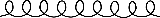

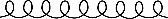

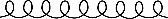

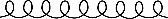

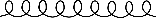


NP_001077.2 ---MGRKSLYLLIVGILIAYYIYTPLPDNV-EEPWRMMWINAH--LKTIQNLATFV-ELL 53

NP_065413.1 ----MGRTIFLLISVVLVAYYIYIPLPDDI-EEPWKIILGNTL--LKLGGDLASFG-ELL 52

NP_075872.1 ----MGKTISLLISVVLVAYYLYIPLPDAI-EEPWKVVWETAF--VKIGTDLASFG-ELL 52

XP_001145851.1 ---MGRKSLYLLIVGILIAYYIYTPLPDNV-EEPWRMMWINAH--LKTIQNLATFV-ELL 53

XP_534309.2 ---MGRKTILLLIMGVFWAYYIYIPLPDNI-EESWKLLCMTTY--WKILTDLSLFA-ELL 53

XP_001106694.1 ---MGRKSLYLLIGGILIAYYIYTPLSDNI-EEPWRLMWINAQ--QKTIENLATFV-ELL 53

XP_028929988.1 ---MGRSTLCFLISFALLAYYLYTPLPDNT-EETWKLIVIHNG--FKTMQYLATLT-EIL 53

XP_019652071.1 ---MGRKTLLLMIMGVFVAYYIYIPLPENI-EEPWKLLCMTTC--MKTVIDVILFA-ELL 53

XP_003416197.1 ---MGRKSLYLLIVGVLVAYYVYTPLPDNF-EEPWKVMWLDTQ--IRTAAHLATFL-ELL 53

XP_003511717.1 ----MGKTILLLVAVVLVAYYVYVPLPGDI-EEPWKLMLQTTR--LKVMMDLVSFA-ELL 52

XP_003925073.1 ---MGRKSLYLLIVGIFIAYYIYIPLPDNT-EEPWRMMWLTTQ--LNTAEKLAAFV-ELL 53

XP_004037911.1 ---MGRKSLYLLIVGILIAYYIYTPLPDNV-EEPWRIMWINAH--LKTIQNLATFV-ELL 53

XP_004682395.1 ---MGRKSLYLLMLGIFVAYYIYIPLPDNF-EEPWRMMFTNAY--LNTIKYSVYFV-ELL 53

XP_005077976.1 ----MGRTFFLLVAAVLVAYYVYVPLPGDI-EEPWKLMLETTR--LKVMMGLISFA-EFL 52

XP_005344092.1 ----MGRTILLLMTVVLVAYYVYTPLPGDI-EEPWKLILEITTGNLELSYQEASLA-ELL 54

XP_006099319.1 ---MGRKTILLLLVGIFTAYYGYTPLPDNI-EEPWKLIGMTFI--SSTISNVALVG-EVL 53

XP_006202876.1 ---MRKKFFGSLILGLLIANYIYEPLPDNV-EEPWKIMLLNAF--LKTTSHLTLFA-EIL 53

XP_006733329.1 ---MGRKTLLLLIMGVFMAYYIYIPLPDNI-EEPWKLLCLSVC--LKTVTDLIMFA-ELL 53

XP_006771196.1 ---MGRKTILLLLVGIFTAYYGYTPLPDNI-EEPWKLIGLTFI--LNTISNVALVG-EVL 53

XP_006901609.1 ---MGRKTGVLLIVGVFLAYYIYTPLPDNI-EEPWRLMFFNTL--LKSMTHLATFA-EVL 53

XP_006908288.1 ---MGRKSLYLLILGVLVAYYVYIPPPIDF-EDPWKLIWLNAK--MKTIIHLANFI-ELL 53

XP_006994722.1 ----MARTILLLVAVVLVAYYIYVPLPGDI-EEPWKLLLETTT--LKIIMDLFSFT-ELL 52

XP_007529626.1 ---MGRGTVLLLIVGAIVAYYVYTPLPDDV-EEPWKVLWIYTH--MKTLKDLTLFA-EFL 53

XP_008006884.1 ---MGRKSLYLLIGGILIAYYIYTPLSDNI-EEPWRLMWINAH--LKTIENVATFV-ELL 53

XP_008584385.1 ---MGKIYFYFLILGVLIAYYIYTPLPDNI-EEPWKIMLFNAL--MKTSSYLALFA-EIL 53

XP_008843029.1 ---MGRKTILFLFAAVLMAYNAYTPVPDGI-EEPWRLVWEMTQ--MKMMTDLVWMA-ELL 53

XP_010370405.1 ---MGRKSLYLLIGGILIAYYIYTPLSDNI-EEPWRLMWINTH--LKTIENLVTFV-ELL 53

XP_010950295.1 ---MGKKFFHSLILGLLIANYIYEPLPDNV-EEPWKIMLLNAI--FKTTSYLSLFA-EIL 53

XP_010984995.1 ---MGKKFFDSLILGLLIANYIYEPLPDNV-EEPWKIMLLNAI--FKTTSYLSLFA-EIL 53

XP_011814358.1 MYTMGRKSLYLLIGGILIAYYIYTPLSDNI-EEPWRLMWINAH--LKTIENLATFV-ELL 56

XP_011828136.1 ---MGRKSLYLLIGGILIAYYIYTPLSDNI-EEPWRLMWINAH--LKTIENLATFV-ELL 53

XP_012292585.1 ---MGRKSLYLLTVGIFVAYYIYTPLPDNT-EEPWRIMWLNTQ--LKTAEKIAVFV-ELL 53

XP_012646746.1 ---MGKKMILFLIVGVLGAYYIYTPFPDNI-EKPWKLVWTTTN--LKMVTDVAWFA-ELL 53

XP_012889095.1 ---MKKKATFLLIAGVLGAWFIYIPLPASV-EESWKLTWIMVP--IRATTGLALFA-DYL 53

XP_014705074.1 ---MGRKSLYLLSVGILLAYYVYTPLPENF-EEPWRMMLFNTY--LKSAVHLATFL-EML 53

XP_016078067.1 ---MGRKPLYFLIVGVFLAYYVYLPLPEDF-EEPWKIIQLNAY--IKTIIHLAEFV-ELL 53

XP_017710441.1 ---MGRKSLYLLIGGILIAYYIYTPLSDNI-EEPWRLMWINTH--LKTIENLVTFV-ELL 53

XP_020039411.1 ---MGRKTTLFLIAGVLGAYSVYTPLPDNI-EEPWKLTWSTTQ--MKMTMNMALFA-ELL 53

XP_021052676.1 ----MGRTIFLLISVVLVAYYVYIPLPDAI-EEPWTIGRKAAI--VKIGTDLASFG-ELL 52

XP_021516657.1 ----MGRTILLLISIVLAAYYVHVPLPDNI-EEPWRVVWEVTQ--VKIATDLASFA-ELL 52

XP_023087853.1 ---MGRKSLYLLIGGILIAYYIYTPLSDNI-EEPWRLMWINAN--LKTIENLATFV-ELL 53

XP_024415983.1 ---MGRKTLLLLMLGVLGAYYVYTPLPDNV-EEPWKLIWMNTL--LKTTQNLALFA-ELL 53

XP_025231688.1 ---MGRKSLYLLIGGILIAYYIYTPLSDNI-EEPWRLMWINAH--LKTIENLATFV-ELL 53

XP_025291926.1 ---MGRKTILLLIMGVFWAYYIYIPLPDNI-EESWKLLCMTTY--WKILTDLSLFA-ELL 53

XP_025871547.1 ---MGRKTILLLIMGVFWAYYIYIPLPDNI-EESWKLLCMTTY--LKILTDLSLFA-ELL 53

XP_026263974.1 ---MGRRSWCLLLLGVLVAYCIYTPLPENI-EEPMP---INTP--LKALAHLATFLVEFL 51

XP_027789579.1 ---MGRMSWFLLLLGVLAAYCIYTPLPENI-EEPML---TNTP--LKALAHLATFLGLDR 51

XP_028619475.1 ----MGRTIFLLISVVLVAYYVYIPLPDAI-EEPWKLVWQNTL--PKIATDLASFV-ELL 52

XP_028746441.1 ----MARTILLLVAVVLVAYYIYVALPGDI-EEPWKLILETTP--LKIIMDLFSFA-ELL 52

XP_006034098.1 ---MGTKSLFLLTAAVLVAYYVYTPLPENI-EERWKLMLISAG--FRSVGHMAG-LAERL 53

XP_019358692.1 ---MGTKSLFLLTAVVLVAYYVYTPLPENI-EERWKLMLISAG--FRSVGHMAG-LAEQL 53

XP_019388142.1 ---MGTKSLFLLTAAVLVAYYAYTPLPENI-EERWKLMLISAG--FRSVGHMAG-LAERL 53

XP_018426679.1 ---MASKSVFFVLVTVLLAYYIYKPLPDNV-EEKFKVMLLDAT--FRTLGHAGY-LAELL 53

XP_004943624.1 ---MGAKLLCLCIAFTLVAYYIYSPIPENV-EQPWKLMLVSTV--FRTLGHVAE-AAERL 53

XP_003209327.1 ---MGAKLFCLCIAFTLVAYYIYSPIPENI-EQPWKLMLTSAV--LRTLGHMGE-AAEWL 53

XP_005023727.2 ---MGAKLLCFCLASALVSYYIYTPIPENI-EEGWKVMLVTSL--FRTVGHVAE-VADRL 53

XP_005233810.1 ---MGAKLLCLFLASVLLAYYVYTPIPEDF-EEPWKLMLITAS--FRAIGHLAE-VADRL 53

XP_005434343.1 ---MGAKLLCLFLASVLLAYYVYTPIPEDF-EEPWKLMLITAS--FRAIGHLAE-VADRL 53

XP_003218232.1 ---MGKKSLCLLIASVLIAYYVYNPLPENN-EEGWKLMLIDAG--FRTLGHMAG-IAEKL 53

XP_007439720.1 ---MGGKSFCLVVALALIAYYIYSPLPENV-DEPWKLMLLNAA--LRIVEHGGM-IVEKL 53

XP_013910823.1 ---MGGKFFYLLVASTLIAYYIYQPLPENV-EEPWKVMLLDAS--FRSLIHVAT-IVEKL 53


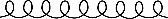

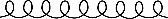

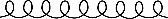

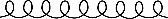

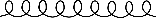


XP_015275105.1 ---MGIKFRALLLASVLVAYYIYTPIPENVKEDRWKLMFLSAL--FRSVGHLAT-VAEKL 54

XP_015669807.1 ---MGGKFFFFLVASALLAYYIYRPVPENL-EEPWKLMLLDAS--FRSLMHVAT-IVEKL 53

XP_020637486.1 ---MGKKSLCLLIASLLIAYYLYSPIPENI-EDPWTLMFLSAV--FRTVGHVAD-IAEKL 53

XP_026520917.1 ---MRGKFFYLLVASTLIAYYIYTPLPENV-EEPWKVLLLDAA--IRSLRHVAT-IVEKL 53

XP_026557410.1 ---MGGKFFYLLVASTLIAYYIYTPLPENV-EEPWKVLLLDAA--IRSLRHVAT-IVEKL 53

XP_028587147.1 ---MGIKLLCLLIATVLTAYYIYSPLPENV-EDRWKLMLIGAV--FRTLDHVGS-VANKL 53

XP_014425448.1 ---MGTKTLCLLLASLLVAYYVYTPVPENI-EERWKLMLINAG--FRTIEHVAE-LTEWL 53

XP_021013481.1 ----MGRTIFLLISVVLVAYYVYIPLPDAI-EEPWNVVWKTAF--VKIGTDLASFG-EFL 52

XP_032752628.1 ----MGRTIFLLISVVLVAYYIYIPLPDDI-EEPWKIILGNTL--LKLGGDLASFG-ELL 52

XP_032887718.1 ---MGIKLVSFAVVSVLLGYYVYLPLPEAL-DERWKLMMVDAC--FRSLEHLAE-LTEFI 53

XP_029884721.1 ---MGAKLLCLFLASALLAYYIYTPIPEDL-EEPWKVMFITAT--FRAMGHLAE-VADQL 53

XP_034357074.1 ----MGRTVFLLISVVLVAYYVYKPLPDAI-EEPWKLVWQTTL--PKIATDLASFV-ELL 52

XP_032048897.1 ---MGAKLLCFCLASALVAYYIYTPIPENI-EEGWKVMLITSL--FRTVGHVAE-VADRL 53

XP_008498425.2 ---MGAKLLGFCLVSALLAYYLYTPIPEDI-EEPWKAMLVATS--FRVMGQLAE-VADKL 53

XP_030810306.1 ---MGARLLCLCLLTALLGYYIYSPLPPEL-AQPGTVMLASAA--LRALGHLSE-VAELL 53

XP_006189146.2 ---MGKKFFHSLILGLLIANYIYEPLPDNV-EEPWKIMLLNAI--FKTTSYLSLFA-EIL 53

XP_032924696.1 ---MGSKLLCLCLLAALLGYYIYIPLPEGM-TEPWPVMLISAV--LRTLAHLSE-AADML 53

XP_032630208.1 ---MGTKSLCLLLASVLVAYYVYTPIPENI-EERWKLMLINAG--FRTVSHVAE-FAEWL 53

XP_032554164.1 ---MGAKLLCLLLLPALLAYYIYTPLPAEL-AQPWRVMVLCTP--FRAVGHLSA-VLDLL 53

XP_006868037.1 ---MQRKSLYLLIVGVLIAYYVYIPLPDNF-EEPWRIMWLNTH--VKTLLNLATFV-ELL 53

XP_031975985.1 ---MGARLLCLCLLTALLGYYIYTPLPEDL-AEPWKVMLVSAA--FRAVGHLSE-AADLL 53

XP_035422447.1 ---MGAKLLCFCLASALVAYYIYTPIPENI-EEGWKVMLVTTM--FRTVRHAAE-VADRL 53

XP_033814578.1 ---MAPKLFCFLIVSVLVAYYVYWPLPENV-EEGWKVMIADSI--LRSLGHVGG-LSELL 53

XP_035931406.1 ---MGRKTLLLLIMGVFMAYYIYIPLPDNI-EEPWKLLCLSVC--LKTVTDLIMFA-ELL 53

XP_032028132.1 ---MGRKSLYLLTLGILIAYYIYTPLPDNV-EEPWRIMWINAH--LKTMQNLATFV-ELL 53

XP_033006343.1 ---MGIKSLCLLLATVLTAYYIYSPLPENV-EDRWKLMLIGAV--FRTLDHVAT-VANKL 53

XP_017934775.3 ---MGAKLLCLCLLPALLAYYIYTPVPAEL-AQPWRVMVLCAP--FRAVGHLSA-VLDLL 53

XP_031231873.1 ----MGRSIFLLISVVLVAYYVYIPLPDAI-EKPWWVFREATL--IKIGTYLASFG-ELL 52

XP_005141224.2 ---MGSKLLCLFLASVILAYYIYSPMPEDF-EEPWKVMFISAT--LRTLKHLAE-VTEYL 53

XP_036244957.1 ---MGARLLCLCLLTALLGYYIYSPLPQEL-AQPGTVMLASAA--LRALGHLSE-AAELL 53

XP_036207699.1 ---MGRKTILLLLVGIFTAYYGYTPLPDNI-EEPWKLIGWTFI--FNTITNVALVG-EVL 53

XP_012365981.2 ---MGRKSLYLLIVGILIAYYIYTPLPDNV-EEPWRIMWINAH--LKTMQNLATFV-ELL 53

XP_036045372.1 ---MGRRTILLLAAMVLVAYYVYIPLPDDI-EEPWNLILEMTK--LKIVVDLVSFA-EFL 53

XP_008950113.1 ---MGRKSLYLLIVGILIAYYIYTPLPDNV-EEPWRMMWINAH--LKTIQNLATFV-ELL 53

XP_034295103.1 ---MGGKFFYLLVASTLIAYYIYRPLPENV-EEPWKLMLLDAG--FRSLIHAAT-IVEKL 53

XP_003894989.2 ---MGRKSLYLLIGGILIAYYIYTPLSDNI-EEPWRLMWINAH--LKTIENLATFV-ELL 53

XP_031459500.1 ---MGAKLLCFCVAFTLVAYYIYSPIPENI-EQPWKLMLMSAV--FRTLGHVAE-AAERL 53

XP_032264231.1 ---MGRKTLLLLIMGVFMAYYIYIPLPDNI-EEPWKLLCLSVC--LKTVTDLIMFA-ELL 53

XP_036301415.1 ---MGRKIILLLLVGIFTAYYVYTPLPDNI-EEPWKIIGFTLI--FKTLTNVALVG-ELL 53

XP_029472042.1 ---MATKLLCFLLASVLIAYYVYRPLPENI-EEGWKVMILDTV--LRSVGHMAG-VAELL 53

XP_032118892.1 ---MGRKSLYLLIVGIFVAYYIYMPLPDNI-EEPWRMMWLNTQ--LKTAEKLAAFV-ELL 53

XP_030351736.1 ---MGSKLLCLVLASIVLAYYVYSPIPEDS-EEHWKVMLITAT--FKTFRNLAG-VAEYL 53

XP_030136362.2 ---MGARLLCLGLLAALLGYYIYSPLPEDV-AQPWTVMLTSAA--LRALAHLSE-AAELL 53

XP_037380609.1 ---MGRKSLYLLILGVLVAYYVYIPLPDNF-EEPWRMMLTNAH--LNTIGHLANFV-QLL 53

XP_034637747.1 ---MGTKSLCLLLASVLVAYYVYTPIPENI-EERWKLMLINAG--FRTVAHVAE-FAEWL 53

XP_033060172.1 ---MGRKSLYLLIGGILIAYYIYTPLSDNI-EEPWRLMWINTY--LKTIENLATFV-ELL 53

XP_032842734.1 ---MGARLLCLFLASALVAYYIYTPIPEDM-GEPWKVMLISAS--FRAIGHLAG-AAEQL 53

XP_034989473.1 ---MGIKSLCLLIATVLTAYYIYSPFPENV-EDRWKLMFIGAV--FRTLDHVAA-VANKL 53

XP_005525205.1 ---MGTKLLCLCLLTALLGYYIYSPLPQDL-AEPWTVMLVSAA--FRAVGHLSE-AAELL 53

XP_009277639.1 ---MGAKLLCLFLASALVAYYVYIPIPEDF-EEPWKVMLIAAA--FRAATHLAE-VADQL 53

XP_009332236.1 ---MGAKLLCLFLASALVAYYVYIPIPEDF-EEPWKVMLIAAA--FRAATNLAE-VADQL 53

XP_009507799.1 ---MGAKLLCLFLISVLVAYYVYTPIPEDF-EEPWKVMLITAG--FRAVGHLSE-VADQL 53

XP_009565977.1 ---MGSKLLCVFLASVLLAYYIYIPLPEDF-SEPWKVMLISAT--LKTFGHLAG-AAEQL 53

XP_009575589.1 ---MGAKLLGLCLASALLAYYIYSPMPEDF-EEPWKVMLITAA--FRAVRHVSE-VADQL 53

XP_009632710.1 ---MGVKLLCLFLASALVAYYIYTPIPEDV-EEPWKVMLITAG--FRAVGHLVSLVLSLL 54

XP_009674653.1 ---MGTKSLCLFIASALIAYYIYAPIPENL-EDRWKLMLMSSV--YKTVGHLAE-FAEWL 53

XP_009818640.1 ---MGAKLLCLCLTSALVAYYIYTPVHEDF-EEPWKVMLISAG--FRAVGHLSE-VAAQL 53

XP_009882692.1 ---MGAKLLCLFLASALVAYYIYTPMPEDL-EEPWKLMLITAS--FRAVGHMAE-VADRL 53

XP_009922085.1 ---MGAKLLCLFLASALLAYYIYTPIPEDL-EEPWKVMFITAA--FRAMGHLAE-VADRL 53

XP_009940690.1 ---MGAKLLWLFLASALVAYYIYSPMPEDF-DEPWKVMLISAS--FRTTGHLAE-VADRL 53

XP_010017334.1 ---MGSKLLCLFLASVILAYYIYSPIPEDF-DEPWKVMLIAAS--FRTIEHLAE-VAEYL 53

XP_010157376.1 ---MGAKLLCLLLTSVLLAYYLYTPIPEDF-EEPWKVMLITAS--FRAVGHLAE-VADRL 53


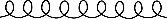

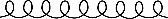

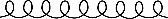

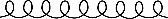

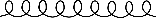


XP_010171133.1 ---MGTKLLCFVLASALLAYYLYTPVPEDI-DEPWKVMLLSTS--FRVMGHLAE-VTDWL 53

XP_010190764.1 ---MGAKLLWLCLASALAAYYIYSPIPAGL-GQPWKVMLLDTG--FRAMGHLAE-VAEQL 53

XP_010296603.1 ---MGAKLLCLFLASALTAYYIYTPLPEDI-EEPWKVMLVTAG--FRAVGHLAE-VADQL 53

XP_010583207.1 ---MGAKLLCLFLASALLAYYIYTPIPEDL-EEPWKVMFITAA--FRAMGHLAE-VADRL 53

XP_013054760.1 ---MGAKLLCFCLASALVAYYIYTPIPENI-EEGWKVMLVTTM--FRTVGHVAE-VADRL 53

XP_013797768.1 ---MGTKSLCLFIASVLIAYYIYTPMPENF-EDRWKLMLITAV--FRTVEHLAG-FAERL 53

XP_014801236.1 ---MGAKLLCLFLVSALIAYYIYTPIPEDI-EEPWKVMLITAS--FRAMGHLAE-VADQL 53

XP_015493839.1 ---MGTKLLCLCLLTALLGYYIYSPLPQDL-AEPWTVMLLSAA--FRAVGHLSE-AAELL 53

XP_015727689.1 ---MGAKLLCLCIAFTLVAYYIYTPIPENI-EQPWKLMLISAV--FRTLGNVAE-VAEQL 53

XP_017690958.1 ---MGAKLLCLCLLPALLAYYIYTPMPAEL-AQPWKVMVLCAP--FRAVGHLSA-VLDLL 53

XP_021251550.1 ---MGAKLLCLFIASALVAYYIYSPIPENI-EEPWKLMLTSAA--FRTLGHMAE-AAERL 53

XP_021403179.1 ---MGARLLCLCLLTALLGYYIYSPLPEDL-AQPWTVMLTSAS--LRALGHLAE-AAELL 53

XP_023788489.1 ---MGTKLLCLCLLAALLGYYIYSPLPQDL-AEPWTVMLVSAA--FRAVGHLSE-AAELL 53

XP_025920950.1 ---MGTKSLCLFIASVLIAYYIYTPMPENF-EDRWKLMLITAV--FRTVEHLAG-FAERL 53

XP_026711093.1 ---MGAKLLWLFLTSALLAYYIYTPIPEDI-GEPWKVMLLSAG--FRTMGHLAE-VMDQL 53

XP_027503068.1 ---MGAKLLCLFLLPALLAYYIYTPLPAEL-AQPWRVMVLCAP--FRAVGHLSA-VLDLL 53

XP_027541513.1 ---MGAKLLCLCLLPALLAYYIYTPLPADL-AEPWTVMFASAP--FRAVGHLAT-ALDLL 53

XP_027587074.1 ---MGAKLLCLCLLPALLAYYIYIPLPAEL-AQPWRVMVLCAP--FRAVGHLSA-VLDLL 53

XP_027747710.1 ---MGAKLLCLCLLPALLAYYIYSPMPEGL-AEPWKLMVLLTP--FKAVGHLAA-AVDLL 53

. . : .


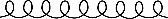


NP_001077.2 GLHHFMDSFKVVGSFDEVPPTSDENVTVTETKFNNILVRVYVPKRKSEALRRGLFYIHGG 113

NP_065413.1 GLNHFMDTVQLFMRFQVVPPTSDENVTVMETDFNSVPVRIYIPKRKSTTLRRGLFFIHGG 112

NP_075872.1 GISHFMETIQLLMSFQEVPPTSDEHVTVMETAFDSVPVRIYIPKRKSMALRRGLFYIHGG 112

XP_001145851.1 GLHHFMDSFKVVGSFDEVPPTSDENVTVTETKFNNILVRVYVPKRKSEALRRGLFYIHGG 113

XP_534309.2 GVNHFMNIFMFFMNLQEVPPTSDENVTVMETTFNNVPVRVYVPKRKPERLRRGLFYIHGG 113

XP_001106694.1 GLHHIMDSLKVVWSFYEVPPTSDENVTVTETKFNNILVRVYVPKRKSEVLRRGLFYIHGG 113

XP_028929988.1 GLAHYMDVINAVTNQGNVGPISDENITVTDTTFNGIPVRLYMPKRKSDTLKRGVFFIHGG 113

XP_019652071.1 GINHLLNMATFFVSLQEVPPTSDENVTVMETTFNNIPVRIYVPKRKPERLRRGLFYVHGG 113

XP_003416197.1 GTGHYMDFLMFTLSFAEVPPTSDENVTVSETTFNNIPVRVYVPKRKSAALRRGLFYIHGG 113

XP_003511717.1 GINHYMDTIQILMKFQELPATSDENVTVTDTAFNSVPVRIYVPKRKSESLRRGLFFIHGG 112

XP_003925073.1 GLHHIMDSLMFALSIYEVPPTSDENVTVTETKFNNILVRVYVPKRKSEALRRGLFYIHGG 113

XP_004037911.1 GLHHFMDSFKVVGSFDEVPPTSDENVTVTETKFNNILVRVYVPKRKSEALRRGLFYIHGG 113

XP_004682395.1 GLCNSMDSMQIFTSFLDAPPTSDENVTVTDTTFNNIPVRVFMPKRKSEALRRGLFYIHGG 113

XP_005077976.1 GINHSMDTIQLLMRFQEVPATSDENVTVTDTTFNSVPVRIYVPKRKTESLRRGLFFIHGG 112

XP_005344092.1 GISHFMSTIHLFSRFQEVPATSDENVTVIDTAFNSVPVRIYMPKRKSETLRRGLFYIHGG 114

XP_006099319.1 GINHFMNTLMFFKSFQDVPPTSDEIVTVMDTTFSNVPVRVYVPKRKSEALRRGLFYIHGG 113

XP_006202876.1 GLNHFMESMMFFSRFQNVPPTSDENVTVRDTTFNDIPVRIYVPQRKPKSLRRGLFYIHGG 113

XP_006733329.1 GMNHFMNIAMFFVNLQEVPPTSDENVTVTETTFNNIPVHVYVPKRKPERLRRVLFYVHGG 113

XP_006771196.1 GINHYMNTMNFFMSFPKVPPTSDETVTVMDTTFSNVPVRVYVPKRKSEALRRGLFYIHGG 113

XP_006901609.1 GISNLFDTMKLFSPFDEVPPTSDENVTVTETTFNNVPVRVYVPRRKSETLRRALFYIHGG 113

XP_006908288.1 GLYNFMDIIKILLHVKEVPPISDENITVTDTTFNHIPVRVYVPKKQSKALRRGIFYIHGG 113

XP_006994722.1 GINHLMDSVQFLMRFQEVPATSDENVTVTETTFDGVPVRIYMPKRKSEALRRGLFFIHGG 112

XP_007529626.1 GMNHFMSSAKIFMSFHEVPPTSDENVSVMETTFDNVPVRVYVPKRKSETLRRGVFYIHGG 113

XP_008006884.1 GLHHIMDSLKVVWSFYEVPPTSDENVTVTETKFNNIPVRVYVPKRKSEVLRRGLFYIHGG 113

XP_008584385.1 GINHFMESIVFLSSFLEVPPTSDENVTVRDTTFNNIPVRIYVPKRKPESLRRGLFYIHGG 113

XP_008843029.1 GINHFMDSMVFLMSLHNVPPTSDENVDVMDTTFNSVPVRVYVPKRQSETLRRGLFFIHGG 113

XP_010370405.1 GLHHIMDSLQVVWSFYEVPPTSDENVTVTETQFNNILVRVYVPKRKSEVLRRGLFYIHGG 113

XP_010950295.1 GLNNSMESMMFFSRFQSVPPTSDENVTVRDTTFNDIPVRIYVPQRKPKSLRRGLFYIHGG 113

XP_010984995.1 GLNNSMESMMFFSRFQSVPPTSDENVTVRDTTFNDIPVRIYVPQRKPKSLRRGLFYIHGG 113

XP_011814358.1 GLHHIMDSLKVVWSFYEVPPTSDENVTVTETKFNNILVRVYVPKRKSEVLRRGLFYIHGG 116

XP_011828136.1 GLHHIMDSLKVAWSFYEVPPTSDENVTVTETKFNNILVRVYVPKRKSEVLRRGLFYIHGG 113

XP_012292585.1 GLHHIMDSLMFALSFYEVPPTSDENVTVTETKFNNIPVRVYVPKRKSEALRRGLFYIHGG 113

XP_012646746.1 GISHIMDTLNFFMSFLEVPITSDENVTVTETTFNNVPVRVYVPNRKSEILRRGLFYIHGG 113

XP_012889095.1 GIIHFANSLMFFMGFLEVPPTSDENIIVTETFFNSVPVRVYVPKRKSEILKKGLLYVHGG 113

XP_014705074.1 GLNHLMDSMMIGMSFDEVPPTSDENVTVTETTFNHIPVRVYVPKRKSEALRRGVFYIHGG 113

XP_016078067.1 GLNNFVDSLTIFLGPSEVPPTSDENITVTETTFNHIPVRVYVPKRKSEALRRGVFYIHGG 113

XP_017710441.1 GLHHIMDSLQVVWSFYEVPPTSDENVTVTETQFNNILVRVYVPKRKSEVLRRGLFYIHGG 113

XP_020039411.1 GINHLMSTLMFLLNFAEVPPTSDENVTVTETTFNSVPVRVYIPKRKSETLKRGLFYIHGG 113

XP_021052676.1 GISHFMETMQLLMKFQEVPPTSDENVTVMETNFNSIPVRIYIPKRKSMTRRRGLFYIHGG 112

XP_021516657.1 GISHFMDTVKLFMKLHEVPPTSDENVTVMETTFNSVPVRIYIPKRESAPLRRGLFYIHGG 112


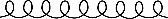


XP_023087853.1 GLHHIMDSLKVVWSFYEVPPTSDENVTVTETKFNNILVRVYVPKRKSEVLRRGLFYIHGG 113

XP_024415983.1 GINHLMDTLMFYMSFQEVPPTSDENVTVTETTFNNVPVRVYIPHRKSQALRRGVFYIHGG 113

XP_025231688.1 GLHHIMDSLKVVWSFYEVPPTSDENVTVTETKFNNILVRVYVPKRKSEVLRRGLFYIHGG 113

XP_025291926.1 GVNHFMNIFMFFMNLQEVPPTSDENVTVMETTFNNVPVRVYVPKRKPERLRRGLFYIHGG 113

XP_025871547.1 GVNHSMNIFMFFMSFQEVPPTSDENVTVMETTFNNVPVRVYVPKRKPERLRRGLFYIHGG 113

XP_026263974.1 GFD----FFKPMMIFLEVPPTSHKNVIVTDTKFNNTPVRVYVPRRKSDTLRRSVFYIHGG 107

XP_027789579.1 YFD----FFTLMMIFPEVPPTSDKNVIVTDTKFNNTPVRVYVPRRKSDTLRRSVFYIHGG 107

XP_028619475.1 GINHLMDTIQLLITFQEVPPTSDENVTVMETTFDSVPVRIYIPKRKSTTLRRGLFFIHGG 112

XP_028746441.1 GINHSMDSLQFLMRFQEVPATSDENVTVTETTFNGVPVSIYMPKRKSETLRRGLFFIHGG 112

XP_006034098.1 GLMHYMEVLMMITSYEYVPATSDENVTVTDTEFENVPVRLYVPRKQSSELKRAVIYIHGG 113

XP_019358692.1 GLMHYMEVLMMITSYEYVPPTSDENVTVTDTEFENVPVRLYVPRKQSSELKRAVIYIHGG 113

XP_019388142.1 GLMHYMEVLMMITSYEYVPPTSDENVTVTDTEFENVPVRLYVPRKQSSELKRAVIYIHGG 113

XP_018426679.1 GVTHYMDVMMLLTKVEHTEPISDENVTVTDTSFSNVPVRLYVPKRQSEGPRRAVIYIHGG 113

XP_004943624.1 GLANYMDVLMLFSTAEHVAPTSDENVTVTDTELSGVAVRLFLPKKPADGLQRAVLYFHGG 113

XP_003209327.1 GLANYMDVLMLLSATEYMAPTSDENVTVADTEFSGVAVRLFLPKKPTEGLRRAVLYFHGG 113

XP_005023727.2 GMMHYMEVMRLITVAEHVAPTSDDNVTVTDTEFSNVAVRLYLPRKPASGLRRAVIYFHGG 113

XP_005233810.1 GLMHYMEVLMLITAAEYVAPMSDENVTVIDTEFSNIAVRLYLPQKASDGLRRAVIYFHGG 113

XP_005434343.1 GLMHYMEVLMLITAAEYVAPMSDENVTVIDTEFSNIAVRLYLPQKASDGLRRAVIYFHGG 113

XP_003218232.1 GLAHYMETMMMLTYLEYAAPVSDEKVTVTDTKMNDVPVRLYVPKEKPDSLKRAVIFIHGG 113

XP_007439720.1 GLAHYMDVLMMITNIQYTPPASDEKVTVTEAKFNHVPVRLYIPTEQSDVLKRAVIYIHGG 113

XP_013910823.1 GVGHFMDVLNLHTIATYTPPTSDEKVIVTDTEFSHVPVRLYIPTKQSDVLKRAVIFIHGG 113

XP_015275105.1 GFAHYMDVMMLITRIDYTPPVSYEKVIVTDTKFNDVPVRLYIPKGQPDSLKRAMIYLHGG 114

XP_015669807.1 GLGHYMDVMNLYTIMEYTAPTSDEKVIVTDTKFSHVPVRLYIPTKQEDVLKRAMIFIHGG 113

XP_020637486.1 GLAHHMDVLMVITNVESTAPVSDEKTTVTDTAFNGVSVRLYIPKGRPESLKRAVIFVHGG 113

XP_026520917.1 DLAHYMDVINLHTIAEYTAPTSDEKVIVTETNFSHVPVRLYIPIKKSNVLKRAVIFIHGG 113

XP_026557410.1 NLAHYMDVINLHTIAEYTAPTSDEKVIVTDTDFSHVPVRLYIPIKKSNVLKRAVIFIHGG 113

XP_028587147.1 GLAHHMDVMMSLTSFEQTPPTSDEKVTVTDTEFNGVPVRLYIPKGQPDSLKRAMIYIHGG 113

XP_014425448.1 GLMHYMEVLMMITSMEYTAPVSDENMTVTDTKFNNVPVRLFIPKKQSSGLRRAVIYIHGG 113

XP_021013481.1 GISHFMETLQLLMRFQEVPPTSDENVTVMETAFDSVPVRIYIPKRKSMTLRRGLVYIHGG 112

XP_032752628.1 GINHFMDTLQFFLRFQDVPPTSDENVTVMETDFNSVPVRIYIPKRKSTTLRRGLFFIHGG 112

XP_032887718.1 GLKHYMEVMTLLTVAEMVGPMSDENVTVVDTTFNGVAVRVFEPTRRKPGLRRAVIYMHGG 113

XP_029884721.1 GLMHYMETLMLITAAEYVAPTSDENVTVMDTEFSNVAVRLYLPRKASDGLRRAVVYFHGG 113

XP_034357074.1 GISHFMDTMRLLMTFQEVPPTSDENVTVMETTFDSVPVRIYIPKRKSTTLRRGLFYIHGG 112

XP_032048897.1 GLMHYMEVMRLITIAEHVAPTSDENVTVTDTEFSNVAVRLYLPRKPASGLRRAVIYFHGG 113

XP_008498425.2 GLVHYMDTLMLLTDAEYVAPMSDENITVRDTEFSNVSVRLYLPKKPSERLRRAVVYFHGG 113

XP_030810306.1 GLMHYMEGLRLITTVELVAPTSDENVTVTDTELGGVPVRLYLPKRPPGGLRRAVLFFHGG 113

XP_006189146.2 GLNNSMESMMFFSRFQSVPPTSDENVTVRDTTFNDIPVRIYVPQRKPKSLRRGLFYIHGG 113

XP_032924696.1 GLMNYMEVMRLFTTVEIVPPTSDENVTVTEAEFNSVPVRLFLPRRAPGGLRRAIVYFHGG 113

XP_032630208.1 DLMHYMEVLMMITSLEYTAPISDENVTVTDTKFNNVPARLYMPRKQSSGLKRAVIYIHGG 113

XP_032554164.1 GLMHYMEGLRLITAVETIVVTSDENVTVTDTEFNNVPVLLYLPKRAPDRLRRAMVYFHGG 113

XP_006868037.1 GIKHCMETLIIAMSFSEVPVISDENVTVTETTFNNIPVRVYVPKRKSEELRRGLFYIHGG 113

XP_031975985.1 GLMHYMEGVRLITVLELVSPSPDENVTVTDTKFNNVPVRLYLPRRAPDGLRRAMVYFHGG 113

XP_035422447.1 GLMHYMEVLSLITVAEYVAPTSDENITVTDTEFSNVAVRLYLPRKSAGGLRRAVIYFHGG 113

XP_033814578.1 GLSHYMDVMMLLTDFKHTEPTSDENITVTDTTFNKVPVRLYVPKKESNKLRRAVIYIHGG 113

XP_035931406.1 GMNHFMNIATFFVNLQEVPPTSDENVTVTETTFNNIPVRVYVPKRKPERLRRVLFYVHGG 113

XP_032028132.1 GLHHFMDSFKVVGSFYEVPATSDENVTVTETKFNNILVRVYVPKRKSEALRRGLFYIHGG 113

XP_033006343.1 GLAHHMEVMMSLTSFEHTAPTSDEKVTVTDTEFNGVPVRLYLPKGQPNSLKRAMIYIHGG 113

XP_017934775.3 GLMHYMEGMRLITAMETVVVTSDENVTVTDTEFNNVPVLLYLPKRAPDGLRRAMVYFHGG 113

XP_031231873.1 GINHFMDTMQLFMKFQEVPPMSDENVTVMETAFDSVPVRIYIPKRKSMALRRGLFYIHGG 112

XP_005141224.2 GLQNYMEVLTLITNTEYVMPMSDENVTVTDTEFNNTTVRLYLPRKASDGLRRAVVYFHGG 113

XP_036244957.1 GLMNYMEGLRLITTVELVAPTSDENVTVTDTELRGVPVRLYLPKRPPGGLRRAVLFFHGG 113

XP_036207699.1 GINHYMNTVRFFMSFQDVPPTSDETVTVTDTTFSNVPVRVYVPKRKSEALRRGLFYIHGG 113

XP_012365981.2 GLHHFMDSFKVVGSFYEVPATSDENVTVTETKFNNILVRVYVPKRKSEALRRGLFYIHGG 113

XP_036045372.1 GINHFMDSLQFLIRLQEVPATSDENVTVTETTFNSVPVRIYMPKRKSEMLRRGLFFIHGG 113

XP_008950113.1 GLHHFMDSFKVVGSFDEVPPTSDENVTVTETKFNNILVRVYVPKRKSEALRRGLFYIHGG 113

XP_034295103.1 GLGHYMDVINLYTIATYTPPTSDEKVIVTDTKFGHVPVRLYIPTKQSDVLKRAMIFIHGG 113

XP_003894989.2 GLHHIMDSLKVVWSFYEVPPTSDENVTVTETKFNNILVRVYVPKRKSEVLRRGLFYIHGG 113

XP_031459500.1 GLANYMDVLMLLSAAEYMAPTSDENVTVTDTEFSGVAVRLFLPKKPTEGLRRAVLYFHGG 113

XP_032264231.1 GMNHFMNIATFFVNLQEVPPTSDENVTVTETTFNNIPVRVYVPKGKPERLRRVLFYVHGG 113

XP_036301415.1 GINHFMSTVVFFMSFQDVPPTSDETVTVLDTTFSNVPVRVYIPKRKSEALRRGLFYIHGG 113

XP_029472042.1 GLNQYMEVMMLLTAFEHTAPISDENVTVTDTAFNNVPVRLYVPTKPSKKLRRAVIYIHGG 113

XP_032118892.1 GLHHIMDSLGFAWGFYEVPPTSDENVTVTETKFNNVLVRVYVPKRKSEALRRALFYIHGG 113


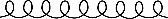


XP_030351736.1 GLMHYMDVVTLIGTMQYVAPMSDENVIVTDTEFNNVTVRLYLPRKASDGLRRAVVYFHGG 113

XP_030136362.2 GLMNYMEGLRLITAVELVAPTSDENVTVTDTEFSGVPVRLFLPRRAPGGLRRAVVYFHGG 113

XP_037380609.1 GISNSMDSFKIFMSFLEVSPTSDENVTVTDTTFNNIPVRVFMPKRKSEALRRGLFYIHGG 113

XP_034637747.1 GLMHYMEVLMMITSIEYTAPISDENVTVTDTKFNNVPARLYIPRKQSSGLKRAVIYIHGG 113

XP_033060172.1 GLHHIMDSLQVVWRFYEVPPTSDENVTVTETKFNNILVRVYVPKRKSEVLRRGLFYIHGG 113

XP_032842734.1 GLVHYMEALMLIMAAEYVAPTSDENVTVTDTEFSNVAVRLYLPRRAADGLRRAVLYFHGG 113

XP_034989473.1 GLAHHMDVMMSLTSFEHTAPTSDEKVTVTDTEFNGVPVRLYLPKGQPDSLKRAMIFIHGG 113

XP_005525205.1 GLMHYMESLRLITVVELVAPTSDENVTVTETEFEQVPVRLYLPRRAAGGLRRAMLYFHGG 113

XP_009277639.1 GLIHHVEALMLITTVEYIAPTSDENVTVMDTEFNNVGVRLYLPRKLSDGLRRAVVYFHGG 113

XP_009332236.1 GLMHHVEALMLITTVEYIAPTSDENVTVMDTQFNNVGVRLYLPRKPSDGLRRAVVYFHGG 113

XP_009507799.1 GLMHYMEALMLITAAEYVAPTSDENVTVTDTEFSNVAVRLYLPRKPSDGLRRAVVFFHGG 113

XP_009565977.1 GLMHYTEALNLLSCFDCVAPTSDENVTVTDTEFNNVAVRLYLPRKVPDGLRRAVVFFHGG 113

XP_009575589.1 GLMHSMEALKVIAAIEYIPPTSDENVTVMDTEFSNVAVRLYLPRKPSDGLRRAVVYFHGG 113

XP_009632710.1 GLMHYMEALMLITAVEYIAPTSDENVTVTDTEFSNVAIRLYLPRKPSDGLRRAVVYFHGG 114

XP_009674653.1 GLMHYMEVLMLITVTEHVPPTSDENVTVTDTEFSNVPVRLYLPRKQLSGLKRAILYFHGG 113

XP_009818640.1 GLMHYMEALMLITAVEYVAPMSDENVTVTDTEFSNVAVRLYLPRKPSDGLRRAVVYFHGG 113

XP_009882692.1 GLMHYMEALMLITAAEYVAPASDENVTVTDTEFSNVAVRLFVPKRAPEGLRRAVIYFHGG 113

XP_009922085.1 GLMHYMETLMLITVAEYVAPTSDENVTVMDTEFSDVAVRLYLPRKASDGLRRAVVYFHGG 113

XP_009940690.1 GLMHYMEALMLISTFQYIAPTSDENVTVTDTEFSNVPVRLFVPRRASDGLRRAVIFFHGG 113

XP_010017334.1 GLKHYMDVLILITTVEYVTPMSDENVTVTDTEFNNITVRLYLPRKASDGLRRAVIYFHGG 113

XP_010157376.1 GLMHYMEALMLITAAEYIAPTSDENVTVSDTEFSGVAVRLYLPRRATDGLRRAVVYFHGG 113

XP_010171133.1 GLMNYMEALMLITTAEYTPPTSDENVTVTDTELSNVAVRLFLPRKPSEGLRRAVIFFHGG 113

XP_010190764.1 GLMHYMDVLMLITFSECVAPSSDENVTVTDTEFSGVAVRLYLPRKAPEGLRRAVVYVHGG 113

XP_010296603.1 GLMHYMEALMLITSAEYVAPTSDENVTVMDTEFSNVAVRLYLPRKASDGLRRAVVYFHGG 113

XP_010583207.1 GLMHYMETLMLITVAEYVAPTSDENVTVMDTEFSDVAVRLYLPRKASDGLRRAVVYFHGG 113

XP_013054760.1 GLMHYMEVLSLITVAEYVAPTSDENVTVTDTEFSNVPVRLYLPRKPAGGLRRAVIYFHGG 113

XP_013797768.1 GLMHYMEALMLITVAEYVPPTSDENVTVTDTEFSNVPVRLYLPRKQSSGLKRGMVYFHGG 113

XP_014801236.1 GLMHYMEGLMLLTTAEYVAPTSDENVTVTDTEFNNVAVRLFLPRRTSDGLRRAVIYFHGG 113

XP_015493839.1 GLMHYMESLRLITVVELVAPTSDENVTVTETEFEQVPVRLYLPRRAAGGLRRAMLYFHGG 113

XP_015727689.1 GLANYMDVMKLFSTAEYTAPTSDENVTVTDKEISGVAVRLFLPQKPRDGLRRAVLYFHGG 113

XP_017690958.1 GLVHYMKGMRLITAMETVMVTSDENVTVTDTEFNNVPVLLYLPKRAPDGLRRAMVYFHGG 113

XP_021251550.1 GLAHYMDVLMLFSATEYVAPTSDENTTVTDTELSGVAVRLYLPKKPADGLRRAVLFFHGG 113

XP_021403179.1 GLMNYMEGLRLFTALELVAPTSDENVTVADTELGSVPVRLFVPRRAPGGLRRAVVYFHGG 113

XP_023788489.1 GLMHYMESLRLITVVELVAPTSDENVTVTETEFEQVPVRLYLPRRAAGGLRRAMLYFHGG 113

XP_025920950.1 GLMHYMEALMLITVAEYVPPTSDENVTVTDTEFSNVPVRLYLPRKQSSGLKRGMVYFHGG 113

XP_026711093.1 GLMHYMEVLTLITAAEYVAPTSDENVTVMDTEFSNVAVRLYLPRRVSDGPRRAVLFFHGG 113

XP_027503068.1 GLMHYMKGLRLITAIETIVVTSDENVTVTDTEFNNVPVLLYLPKRAPDGLRRAMVYFHGG 113

XP_027541513.1 GLMHYMEGMRLITVLETVMVTSDEDVTVTHTEFNNVPVLLYLPKRAPEGLRRAMVYFHGG 113

XP_027587074.1 GLMHYMEGMRLITAMETVMVASDENVTVTDTEFNNVPVLLYLPKRAPDGLRRAMVYFHGG 113

XP_027747710.1 GLMHYMEAMRLITVIETVVATSDENVTVTSTEFNNVPVRLYLPRRAPDGLRRAMVYFHGG 113

. * : :: * :: :.:.***


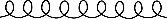

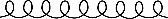

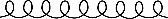


NP_001077.2 GWCVGSAALSGYDLLSRWTADRLDAVVVSTN----------YRLAPKYHFPIQFEDVYNA 163

NP_065413.1 GWCLGSAAYFMYDTLSRRTAHRLDAVVVSTD----------YGLAPKYHFPKQFEDVYHS 162

NP_075872.1 GWCLGSAAHFSYDTLSRWTAHKLDAVVVSTD----------YGLAPKHHFPRQFEDVYRS 162

XP_001145851.1 GWCVGSAALSDYDLLSRWTADRLDAVVVSTN----------YRLAPKYHFPIQFEDVYNA 163

XP_534309.2 GWCLGSAAFLGYDSLSRRTADRLDAVVISTN----------YRLAPKYHFPNQFEDVYNA 163

XP_001106694.1 GWCMGSAAINDYDLLSRWTADRLDAVVISTN----------YRLAPKYHFPIQFEDVYNA 163

XP_028929988.1 GWCIGDKAMKNYDLISRRTANQLDAVVVSTN----------YRLAPKYHFPVQFEDVYTA 163

XP_019652071.1 GWGLGSAAFFSYDFLSRRTADRLDAVVISAN----------YRLAPKYHFPNHFEDVYNA 163

XP_003416197.1 GWCAGSAALSSYDMLSRWTADRLDAVVISTN----------YRLAPKYHFPIQFEDVYNA 163

XP_003511717.1 GWCLGSNALLEYDLLARQIAERLDAVVLSTN----------YGLAPKYHFPRQFEDVYDA 162

XP_003925073.1 GWCLGSAASRYYDLLSRWTADRLDAVVISTN----------YRLAPKYHFPIQFEDVYNS 163

XP_004037911.1 GWCVGSAALSDYDLLSRWTADRLDAVVVSTN----------YRLAPKYHFPIQFEDVYNA 163

XP_004682395.1 GWCLGSAASQNYDLLSRWTADRLDAVVVSTN----------YRLAPKYRFPTQFEDVYNA 163

XP_005077976.1 GWCLGSAALLIYDLLARQAAERLDAVVVSTN----------YGLAPKYHFPRQFEDVYGA 162

XP_005344092.1 GWCLGSAALSMYDLLARQTADRLDAIVVSTD----------YGLAPKYHFPRQFEDVYSA 164

XP_006099319.1 GWCLGSAAQYSYDVLSRRTADRLDAVVISTN----------YRLAPKYHFPNQFEDVYNA 163

XP_006202876.1 GWCWGSNAYKSYDLLSRWTADRLDAVIISTN----------YRLAPKYHFPVQFEDVYTA 163

XP_006733329.1 GWCLGSAALFSYDFLSRQTADRLDAVVISAN----------YRLAPKYHFPNHFEDVYNA 163

XP_006771196.1 GFCLGSAAQYSYDVLSRRTANRLDAVVISTN----------YRLAPKYHFPNQFEDVYNA 163


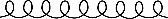

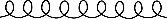

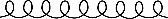


XP_006901609.1 GWCLGSAAMVNYDNLSRLTAHRLDAVVISTN----------YRLAPKYNFPIPFEDVYNS 163

XP_006908288.1 AWTFESAASQNNDLFSRWTADRLDAVVVSPN----------YRLLPKYHFPIQFEDVYNS 163

XP_006994722.1 GWCLGSAASVNYDLLTRQIADRLDAVVVSTN----------YGLAPKYHFPHQFEDVYSA 162

XP_007529626.1 GWCLGSAALLPYDLLSRNTAERLDAIIISTD----------YRLAPEYHFPNQFEDVYHA 163

XP_008006884.1 GWCMGSAAVNDYDLLSRWTADRLDAVVISTN----------YRLAPKYHFPIQFEDVYNA 163

XP_008584385.1 GWCLNSNALFSYDLLSRWTADRLDAVVISTN----------YRLAPKYHFPVQFEDVYTA 163

XP_008843029.1 GWCLGSAAYSPYDVLSRRTADRLNAVVVSTN----------YRLAPKHHFPSQFEDVYNA 163

XP_010370405.1 GWCLGSAAVNDYDLLSRWTADRLDAVVVSTN----------YRLAPKYHFPIQFEDVYNA 163

XP_010950295.1 GWCWGSNAYESYDLLSRWTADRLDAVIVSTN----------YRLAPKYHFPVQFEDVYTA 163

XP_010984995.1 GWCWGSNAYESYDLLSRWTADKLDAVIVSTN----------YRLAPKYHFPIQFEDVYTA 163

XP_011814358.1 GWCLGSAAVNDYDLLSRWTADRLDAVVISTN----------YRLAPKYHFPIQFEDVYNA 166

XP_011828136.1 GWCMGSAAVNDYDLLSRWTADRLDAVVISTN----------YRLAPKYHFPIQFEDVYNA 163

XP_012292585.1 GWCLGSAASYDYDLLSRWTADRLDAVVISTN----------YRLAPKYHFPIQFEDVYNS 163

XP_012646746.1 GWCLGDAAFFDYDLLSRRTADRLDAVVISTN----------YRLAPKYHFPSQFEDVYNA 163

XP_012889095.1 GWCLPGAAFYHYDYLSRSTAHSLDAVVVSVN----------YRLAPKYHFPSQFEDVYNA 163

XP_014705074.1 GWCLGSAALKGYDSLSRWTADRLDAVVISAD----------YRLAPKYHFPTQFEDVYNA 163

XP_016078067.1 GWCHGSAASKQQDLLSRWTADRLDAVVISVS----------YRLAPKYHFPVQFEDAYNS 163

XP_017710441.1 GWCLGSAAVNDYDLLSRWTADRLDAVVVSTN----------YRLAPKYHFPIQFEDVYNA 163

XP_020039411.1 GWCLLSAASFNYDFLSRRTADRLDAVVVSTN----------YRLAPKYHFPNQYEDVYNA 163

XP_021052676.1 GWCLGSAALLTYDTLSRWTAHKLDAVVVSTD----------YGLAPKHHFPRQFEDVYRS 162

XP_021516657.1 GWCLGSAALSMYDSLSRRTADRLDAVVVSTD----------YALAPKHHFPSQFEDVYSA 162

XP_023087853.1 GWCLGSAAVNDYDLLSRWTADRLDAVVISTN----------YRLAPKYHFPIQFEDVYNA 163

XP_024415983.1 GWCLGSAALLSYDLLSRQTVNRLDAVVISTN----------YRLAPNYHFPNQFEDVYNS 163

XP_025231688.1 GWCMGSAAVNDYDLLSRWTADRLDAVVISTN----------YRLAPKYHFPIQFEDVYNA 163

XP_025291926.1 GWCLGSAAFLGYDSLSRRTADRLDAVVISTN----------YRLAPKYHFPNQFEDVYNA 163

XP_025871547.1 GWCLGSAAFFGYDSLSRRTADRLDAVVISTN----------YRLAPKYHFPNQFEDVYNA 163

XP_026263974.1 GWVLGSAAFISYDLLSERTADRLNAVVVSTN----------YRLAPKYHFPVQFEDVYDA 157

XP_027789579.1 GWSLGSAAFISYDLLSRRTADRLDAVVVSTN----------YRLAPKYHFPVQFEDVYDA 157

XP_028619475.1 GWCLGSAASYIYDTLSRWTAHKLDAVVVSTD----------YGLAPKYHFPRQFEDVYRS 162

XP_028746441.1 GWCLGSAAYVNYDLLTRQTADRLDAVVVSTN----------YGLAPKYHFPRQFEDVYNA 162

XP_006034098.1 GWCVGGSAMEPYDLLSRWTSNKLNAVVISVE----------YRLAPKYHFPAQFEDVYKI 163

XP_019358692.1 GWCVGGSAMEPYDLLSRWTSNKLNAVVVSVE----------YRLAPKYHFPAQFEDVYMV 163

XP_019388142.1 GWCVGGSAMEPYDLLSRWTSNKLNAVVVSVE----------YRLAPKYHFPAQFEDVYMV 163

XP_018426679.1 GWCLGSAAMKPYDLLSRKTAHQLSAVVVSIN----------YRLAPKFHFPTQFDDVYAV 163

XP_004943624.1 GWCVGDAGMKGYDFLARRTSSQLNAVVVSVN----------YRLAPKYHFPVQFEDVYSV 163

XP_003209327.1 GFCVGDAGMKAYDFLARRTSSQLNAVVVSVN----------YRLAPKYHFPVQFEDVYSA 163

XP_005023727.2 GWCVGQAGMKSYDLLSRWTSSQLDAVVVSVD----------YRLAPKYHFPVQFEDVYSV 163

XP_005233810.1 GWCLGQAGMKSYDHLTRWTSNRLNAVVVSVS----------YRLAPKYHFPVQFEDVYSV 163

XP_005434343.1 GWCLGQAGMKSYDHLTRWTSNRLNAVVVSVS----------YRLAPKYHFPVQFEDVYSV 163

XP_003218232.1 GWCLGGTAMSSYDLLSRWTSERLNAVVISIE----------YRLAPKYHFPVQFEDVYAV 163

XP_007439720.1 GWCIGSATMKSYDLLSRWTSERLNAVVVSVD----------YRLAPKYRFPVPFEDVYSV 163

XP_013910823.1 GWCTGSATMKSYDLLSRWTSERLNAVVVSVD----------YRLAPKYRFPVAFEDVYSV 163

XP_015275105.1 GWCLGGKGMKTYDFLSRWTSEKLNAVVVSVD----------YRLAPKYHFPVQFEDVYAV 164

XP_015669807.1 GWCIGSANMKSYDLLSRWTSERLTAVVVSVD----------YRLAPKYRFPVAFEDVYSV 163

XP_020637486.1 GWCVGGSAMKSYDNLSRLTSEKLNAVVVSVE----------YRLAPKYHFPVQFEDVYTA 163

XP_026520917.1 GWCIGSATMKSYDLLSRWTSERLSAVVVSVD----------YRLAPKYRFPVAFEDVYSV 163

XP_026557410.1 GWCIGSATMKSYDLLSRWTSERLSAVVVSVD----------YRLAPKYRFPVAFEDVYSV 163

XP_028587147.1 GWCLGSAAMQPYDLLSRRTSETLNAVVVSVE----------YRLAPKHHFPAQFEDVFTV 163

XP_014425448.1 GWCVGGSAMEPYDLLSRWTASKLNAVVVSVEGLIQSSSVKWYRLAPKYHFPVQFEDVYTV 173

XP_021013481.1 GWCLGSAAEFTYDTLSRWTAHKLDAIVVSTD----------YGLAPKHHFPRQFEDVYRS 162

XP_032752628.1 GWCLGSAAYFMYDTLSRQTAHRLDAVVVSTE----------YGLAPKYHFPKQFEDVYRS 162

XP_032887718.1 GWCLGSAKMQPYDALSRKTATELNAVIVSVE----------YRLAPQHHFPDQFNDVYAV 163

XP_029884721.1 GWSLGHAGMKPYDHLTRWTSNRLNAVVVSVN----------YRLAPKYHFPVQFEDVYSV 163

XP_034357074.1 GWCLGSAANYIYDTLSRRTAHKLDAVVVSTD----------YGLAPKYHFPRQFEDVYSS 162

XP_032048897.1 GWCVGQAGMKSYDLLSRWTSSQLDAVVVSVN----------YRLAPKYHFPVQFEDVYSV 163

XP_008498425.2 GWCLGHAGMRSYDQVTRWTSDRLNAVVVSVN----------YRLAPQHRFPAQFEDVYTV 163

XP_030810306.1 GWCLGDAGMHGYDLMSRRISNELNAVVVSVN----------YRLAPLHRFPAQFEDVYVV 163

XP_006189146.2 GWCWGSNAYESYDLLSRWTADRLDAVIVSTN----------YRLAPKYHFPVQFEDVYTA 163

XP_032924696.1 GWCMGDAGMHSYDHMARRFSNELDAVVVSVN----------YRLAPPHHFPVQFEDVYSV 163

XP_032630208.1 GWCVGGSAMEPYDLLSRWTANKLNAVVVSVE----------YRLAPMYHFPVQFEDVYTV 163

XP_032554164.1 GWCLGDAGMKSYDHTSRRTSNELDAVVVSVN----------YRLAPEHRFPVPFEDVYSA 163

XP_006868037.1 GWCLGSAAFHNCDMLSRWTADRLDAVVISTN----------YRLAPKYHFPIQFEDAYNA 163


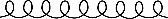

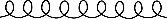

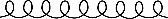
L

XP_031975985.1 GWCLGDAGMKSYDHISRRISNELNAVVVSVN----------YRLAPQHHFPVQFEDVYSV 163

XP_035422447.1 GWCVGQAGMKSYDLLSRWTSNQLDAVVVSVD----------YRLAPKYHFPVQFEDVYSA 163

XP_033814578.1 GWCIGSSAMKAYDDLSRWTVERLNAIVISAD----------YRLAPKYHFPIQFEDSYAV 163

XP_035931406.1 GWCLGSAALFGYDFLSRQTADRLDAVVVSTN----------YRLAPKYRFPNHFEDVYNA 163

XP_032028132.1 GWCIGSAALSDYDLLSRWTADRLDAVVVSTN----------YRLAPKYHFPIQFEDVCNA 163

XP_033006343.1 GWCVGSAAMQPYDLLSRRTSEALNAVVVSVE----------YRLAPKHHFPAHFEDVFTV 163

XP_017934775.3 GWCLGDAGMKTYDHISRRTSNELDAVVVSVN----------YRLAPEHRFPVPFEDVYSA 163

XP_031231873.1 GWCLGSAAYITYDTLSRRTAHKLDAVVVSTD----------YGLAPKHHFPRQFEDVYRS 162

XP_005141224.2 GWCLGQAGMKSYDRLTRRTSDMLNAVVVSVN----------YRLAPKYHFPIQFEDVYSV 163

XP_036244957.1 GWCLGDAGMHGYDLMSRRISNELNAVVVSVN----------YRLAPPHRFPAQFEDVYLV 163

XP_036207699.1 GFCLGSAAQYSYDVLSRRTAKRLDAVVISTN----------YRLAPKYHFPNQFEDVYNA 163

XP_012365981.2 GWCIGSAALSDYDLLSRWTADRLDAVVVSTN----------YRLAPKYHFPIQFEDVYNA 163

XP_036045372.1 GWCLGSAAFVKYDLLARQTADRLDAVVVSTN----------YGLAPKYHFPHQFEDVYSA 163

XP_008950113.1 GWCVGSAALSDYDLLSRWTADRLDAVVVSTN----------YRLAPKYHFPIQFEDVYNA 163

XP_034295103.1 GWCIGSATMKSYDLLSRWTSERLNAVVVSVD----------YRLAPKYRFPVAFEDAYSV 163

XP_003894989.2 GWCIGSAAVNDYDLLSRWTADRLDAVVISTN----------YRLAPKYHFPIQFEDVYNA 163

XP_031459500.1 GFCVGDAGMKAYDFLARRTSSQLNAVVVSVN----------YRLAPKYHFPVQFEDAYSA 163

XP_032264231.1 GWCLGSAALFGYDFLSRQTADRLDAVVISTN----------YRLAPKYRFPNHFEDVYNA 163

XP_036301415.1 GWCLGSATSYSYDTLSRRTADRLDAVVISTN----------YRLAPKYHFPNQFEDVYNA 163

XP_029472042.1 GWCLGSPAMKAYDHLTRWTADRLNAIVVSTD----------YRLAPKYNFPAQFEDAYAV 163

XP_032118892.1 GWCLGSAASHSYDLLSRWTADRLDAVVISTN----------YRLAPKHHFPIQFEDVYNS 163

XP_030351736.1 GWCIGHVGMKPYDLLARWTSNTLNAVVVSVN----------YRLAPKYRFPIQFEDVYSV 163

XP_030136362.2 GWCLGDAGMHGYDLVSRRISNEINAVVVSVN----------YRLAPPHHFPAQFEDVYSV 163

XP_037380609.1 GWCLGSAALQHYDLLSRWTADRLDAVVISTN----------YRLAPQYHFPIQFEDVYNA 163

XP_034637747.1 GWCVGGSAMEPYDLLSRWTANKLNAVVISVE----------YRLAPTYHFPVQFEDVYTV 163

XP_033060172.1 GWCLASAAVNDYDLLSRWTADRLDAVVVSTN----------YRLAPKYHFPIQFEDVYNA 163

XP_032842734.1 GWCLGQAGMRSYDHLARWTSNSLNAVVVSAN----------YRLAPKYHFPVQFEDVYSV 163

XP_034989473.1 GWCVGAAAMQPYDLLSRRTSESLNAVVVSVE----------YRLAPKHHFPAQFEDVYTV 163

XP_005525205.1 GWCLGDAGMHGYDYMSRRISNELNAVVVSVN----------YRLAPPHRFPAQFEDVYVV 163

XP_009277639.1 GWCIGQAGMKPYDRLTRWTSNRLNAVVVSVN----------YRLAPKYRFPVQFEDVYLV 163

XP_009332236.1 GWCLGQAGTKPYDRLTRWTSNRLNAVVVSVN----------YRLAPKYRFPVQFEDVYSV 163

XP_009507799.1 GWCVGQAGMKSYDHLTRWTSSRLNAVVVSVN----------YRLAPKYRFPVQFEDVYSV 163

XP_009565977.1 GWCLGDAAAKNYDLLTRWTSDRLNAVVVSVN----------YRLAPKYSFPIPFEDAYSV 163

XP_009575589.1 GWCLGQAGMKSYDHLTRWTSNRLNAVVVSVN----------YRLGPKYRFPVQFEDVYSV 163

XP_009632710.1 GWCLGQAGMKSYDHLTRWTSNRLNAVVVSVN----------YRLAPKYHFPIQFEDVYSV 164

XP_009674653.1 GWCVGEAGMKPYDLLSRWTSNQLNAVVVSVN----------YRLAPKYHFPVQFEDVYAV 163

XP_009818640.1 GWCVGQAGMKSYDHLSRWTSNMLNAVVVSVN----------YRLAPKYHFPVQFEDVYSV 163

XP_009882692.1 GWCVGHAGMKSYDHLTRWTSNMLNAVVVSVN----------YRLAPKHHFPIQFEDVYSV 163

XP_009922085.1 GWSLGHAGMKPYDHLARWTSNRLNAVVVSVN----------YRLAPKYHFPVQFEDVYSV 163

XP_009940690.1 GWCVGRAGMKSYDHLARWTSNMLNAVVVSVD----------YRLAPKYHFPVQFEDVYSV 163

XP_010017334.1 GWCAGQAGMKSYDHLTRWTSNMLNAVVVSVN----------YRLAPKYHFPIQFEDVYSV 163

XP_010157376.1 GWCVGQAGMKSYDHLTRWTSNKLNAVVVSVN----------YRLAPKYHFPVQFEDVYSV 163

XP_010171133.1 GWCLGHAGMKSYDRLARWTSNRLNAVVVSVN----------YRLAPKYHFPVQFEDVYAV 163

XP_010190764.1 GWCLGQAGMKGYDLLSRWTSHRLNAVVVSVN----------YRLAPKHHFPVQFDDVYAV 163

XP_010296603.1 GWCVGQAGMKGYDYLTRQTSHRLNAVVVSVN----------YRLAPQHHFPVQFEDVYSV 163

XP_010583207.1 GWSLGHAGMKPYDHLARWTSNRLNAVVVSVN----------YRLAPKYHFPVQFEDVYSV 163

XP_013054760.1 GWCVGQAGMKSYDLLSRWTSNQLDAVVVSVD----------YRLAPKYHFPVQFEDVYSV 163

XP_013797768.1 GWCVGGAGMKSYDLLSRQTSNQLNAVVVSVN----------YRLAPKYHFPVQFEDAYSV 163

XP_014801236.1 GWCVGSAGMKPYDHLTRWTSNVLNAVVVSVN----------YRLAPKYHFPIQFEDVYSV 163

XP_015493839.1 GWCLGDAGMHGYDYMSRRISNELNAVVVSVN----------YRLAPPHRFPAQFEDVYAV 163

XP_015727689.1 GWCFGDAGMTAYDLLSRRTASQLNAVVVSVN----------YRLAPKHHFPVQFEDVYSV 163

XP_017690958.1 GWCLGDAGMKSYDHTSRRTSNELDAVVVSVN----------YRLAPEHRFPVPFEDVYSA 163

XP_021251550.1 GWCVGSAGMKSYDLLSRRTSSHLNAVVVSVN----------YRLAPRYHFPVQFEDVYSV 163

XP_021403179.1 GWCVGDAGMRGYDLMSRRISNEINAVVVSVN----------YRLAPPHRFPAQFEDAYSV 163

XP_023788489.1 GWCLGDAGMHGYDYMSRRISNELNAVVVSVN----------YRLAPPHRFPAQFEDVYVV 163

XP_025920950.1 GWCVGGAGMKSYDLLSRQTSNQLNAVVVSVN----------YRLAPKYHFPVQFEDAYSV 163

XP_026711093.1 GWCLGQAGMKSYDQLSRWTSNLLNAVVVSVN----------YRLAPKYHFPTQFEDVYSV 163

XP_027503068.1 GWCLGDAGMKSYDHTSRRTSNELDAVVVSVN----------YRLAPEHRFPVPFEDVYSA 163

XP_027541513.1 GWCLGDAGMKPYDHMARRVSNELDAVVVSVN----------YRLAPEHPFPVPFEDVYAV 163

XP_027587074.1 GWCLGDAGMKSYDHISRRTSNELDAVVVSVN----------YRLAPEHRFPVPFDDVYSA 163

XP_027747710.1 GWCLGDAGMKSYDHMCRRVSNELNAVVVSVN----------YRLAPEHRFPVPFEDVYSV 163

.: * . : *:::* . * * * . ** ::*


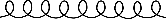

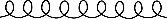

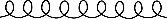

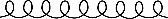


NP_001077.2 LRWFLRKKVLAKYGVNPERIGISGDSAGGNLAAAVTQQLLDDPDVKIKLKIQSLIYPALQ 223

NP_065413.1 LRWFLQEDILEKYGVDPRRVGVSGDSAGGNLTAAVTQQILQDPDVKIKLKVQALIYPALQ 222

NP_075872.1 LRWFLQEDVLEKYGVDPRRVGVSGDSAGGNLAAAVTQQLIQDPDVKIKLKVQALIYPALQ 222

XP_001145851.1 LRWFLHKKVLAKYGVNPERIGISGDSAGGNLAAAVTQQLLDDPDVKIKLKIQSLIYPALQ 223

XP_534309.2 LKGFMRQDVLDKYGVDPERIGISGDSAGGNLAAAVAQQLIDDPDVEINLKTQCLIYPALQ 223

XP_001106694.1 LSWFLRKTVLAKYGVNPERIGISGDSAGGNLAAAVTQQLLDDPDVKIKLKIQALIYPALQ 223

XP_028929988.1 VRWFLRGQILEKYGVDPSRICISGDSAGGNLAAAVNQQLLDDPDVKIKIKIQSLFYPVLQ 223

XP_019652071.1 LKWFLHQHVLDKYGVDPERIGISGDSAGGNLVAAATQQLIDDPDVKIKLKTQSLIYPALQ 223

XP_003416197.1 LRWFLRRTVLAEYGVNPERIGISGDSAGGNLAAAVTQQLLDDPDVKIKLKVQCLIYPALQ 223

XP_003511717.1 LRWFLQEDILERYGVDPQRVGVSGDSAGGNLAAAVAQQIVQDPDVKTKLKVQALVYPALQ 222

XP_003925073.1 LRWFLRKNVLAKYGVNPERIGISGDSAGGNLAAAVTQQLLDDPDVKIKLKIQALIYPALQ 223

XP_004037911.1 LRWFLRKKVLAKYGVNPERIGISGDSAGGNLAAAVTQQLLDDPDVKIKLKIQSLIYPALQ 223

XP_004682395.1 LRWFLRPKVLAQYGVNPERIAVSGDSAGGNLAAAVTQQLLDDPDVKIKLKIQSLIYPALQ 223

XP_005077976.1 LQWFLKEDILERYGVDPRRVGVSGDSAGGNLAAAVTQQLIQDPDVKTKVKVQALVYPALQ 222

XP_005344092.1 LRWFLQEDILERYGVDPRRVGVAGDSAGGNLAAAVTQQLIQDPDVKTKLKVQALIYPALQ 224

XP_006099319.1 LKWFLRKDILKGYGVDPKRIGISGDSAGGNLAAAVTQQLIDDPDVKIKLKIQSLIYPALQ 223

XP_006202876.1 LKWFLHPNVLENYGVDPSRVGISGDSAGGNLAAAVTQQLLEDPDVKIKLKVQSLIYPALQ 223

XP_006733329.1 LKWFLRQDVLDKYGVDPGRIGISGESAGGNLVAAVTQRLIDDPDIKIKLKTQSLIYPALQ 223

XP_006771196.1 LKWFLRKDILKGYGVDPKRIGISGDSAGGNLAAAVTQQLIDDPDVKIKLKIQSLIYPAVQ 223

XP_006901609.1 LKWFLQQDILEKYGVHPERIGVSGDSAGGNLAAAVTQQLLHDPNIKIKLKVQALIYPALQ 223

XP_006908288.1 LKWFLRKEVLAKYGVNPERIGLVGSSSGGNLAAAVVQKILDDPESKFKPKIQSLFYPALQ 223

XP_006994722.1 LRWFLQENILERYGVDPRRVGVSGDSAGGNLAAAVTQQVIQDPDVKTKLKVQALVYPALQ 222

XP_007529626.1 VKWFLRQDVLERYGVDAERIGVAGDSAGGNLAAAVVQQLQDDPDVKIKLKIQALIYPALQ 223

XP_008006884.1 LKWFLRKTVLAKYGVNPERIGISGDSAGGNLAAAVTQQLLDDPDVKIKLKIQALIYPALQ 223

XP_008584385.1 LKWFLHPKFLEGYGVDPGRVGISGDSAGGNLAAVVTQQLLDDPDVKVKLKIQSLIYPALQ 223

XP_008843029.1 LRWFLQQDVLSNYGVDPERVGVSGDSAGGNLAAAVTQQLIKDPDVKIKLKIQALIYPALQ 223

XP_010370405.1 LRWFLHKTVLAKYGVNPERIGISGDSAGGNLAAAVTQQLLDDPDVKIKLKIQSLIYPALQ 223

XP_010950295.1 LKWFLHPNVLENYGVDPRRVGISGDSAGGNLAAAVTQQLLEDPDVKIKLKVQSLIYPALQ 223

XP_010984995.1 LKWFLHPNVLENYGVDPRRVGISGDSAGGNLAAAVTQQLLEDPDVKIKLKVQSLIYPALQ 223

XP_011814358.1 LRWFLRKTVLAKYGVNPERIGISGDSAGGNLAAAVTQQLLDDPDVKIKLKIQSLIYPALQ 226

XP_011828136.1 LRWFLRKTVLAKYGVNPERIGISGDSAGGNLAAAVTQQLLDDPDVKIKLKIQALIYPALQ 223

XP_012292585.1 LRWFLRKNVLANYGVNPERIGISGDSAGGNLAAAVTQQLLDDPDVKIKLKIQALIYPALQ 223

XP_012646746.1 LRWFLHQDVLEKYGVDPERIGVSGDSAGGNLAAAVAQQLIEDPDVKIKLKIQSLIYPALQ 223

XP_012889095.1 LKWFVRQDVLEEYGIDSERIGIAGDSAGGNLAAAVTQQFKDDPDVKIKLKVQALIYPVLQ 223

XP_014705074.1 LKWFLHEKVLAKYGVNPERVAVSGDSAGGNLAAAVTQQLLDDPDVKIKLKVQSLIYPALQ 223

XP_016078067.1 LKWFLRKEVLAKYGVNPERIGVSGSSSGANLAAAVAQQLLDDPDVKIKLKTQSLIYPVLQ 223

XP_017710441.1 LRWFLHKTVLAKYGVNPERIGISGDSAGGNLAAAVTQQLLDDPDVKIKLKIQSLIYPALQ 223

XP_020039411.1 LTWFLRRDVLEKYGVDPERVGVSGDSAGGNLAAAVTQQLIEDPDVKIKLKVQVLVYPALQ 223

XP_021052676.1 LRWFLQEDVLEKYGVDPKRVGVSGDSSGGNLAAAVTQQLIQDPDVKIKLKVQALIYPALQ 222

XP_021516657.1 LRWFLQEDILKKYGVEPTRVGVSGDSAGGNLAAAVTQQLIQDPDVKIKLKVQVLVYPALQ 222

XP_023087853.1 LRWFLRKTVLAKYGVNPERIGISGDSAGGNLAAAVTQQLLDDPDVKIKLKIQSLIYPALQ 223

XP_024415983.1 LKWFLRQDVLEKYGVNPKRIGISGDSAGGNLAAAVTQKLIDDPDVKIKLKTQSLIYPALQ 223

XP_025231688.1 LRWFLRKTVLAKYGVNPERIGISGDSAGGNLAAAVTQQLLDDPDVKIKLKIQALIYPALQ 223

XP_025291926.1 LKGFMRQDVLDKYGVDPERIGISGDSAGGNLAAAVAQQLIDDPDVEINLKTQCLIYPALQ 223

XP_025871547.1 LKWFLRQDVLDKYGVDPERIGISGDSAGGNLAAAVAQQLIDDPDVEINLKTQCLIYPALQ 223

XP_026263974.1 LKWFLRKNILEEYGVDPGRIAVSGDSAGGNLAAAVAQQLLDDPDVKIRLKIQSLIYPALQ 217

XP_027789579.1 IKWFLRKNILEEYGVDPGRIAVSGDSAGGNLAAAVAQQLLDDPDVKIRLKIQCLIYPALQ 217

XP_028619475.1 LRWFLQEDILEKYGVDPRRVGVSGDSAGGNLAAAVTQQLLQDPDIKIKLKVQVLIYPALQ 222

XP_028746441.1 LRWFLQENILERYGVDPRRVGVSGDSAGGNLAAAVTQQTIQDPDVKTKLKVQALVYPALQ 222

XP_006034098.1 LKFILQNNILAQYGIDPSRISVAGDSSGGNLAAAVAQELLDDPEVKIKLKTQALLYPALQ 223

XP_019358692.1 LKFFLQNNILAQYGVDPSRISVAGDSSGGNLAAAVAQELLDDPEVKIKLKTQALLYPALQ 223

XP_019388142.1 LKFFLQNNILAQYGVDPSRISVAGDSSGGNLAAAVAQELLDDPEVKIKLKTQALLYPALQ 223

XP_018426679.1 VRFFLQKSILDKYSVDPNRIAVSGDSAGGNLAAAITQTLFHDPEVKVKLRIQALIYPVLQ 223

XP_004943624.1 SKFFLQSRVLSQYGVDPTRVCVAGDSAGGNLAAAVAQKLLEDSEVTTKLKAQALIYPALQ 223

XP_003209327.1 SKFFLQSRVLSQYRVDPTRVCVAGDSAGGNLAAAVAQKLLEDSEVTNKLKAQALVYPALQ 223

XP_005023727.2 TKFFLQSSVLSQYGVDPNRVCVAGDSAGGNLAAAVVQKLLEDSEVTTRLKAHVLLYPALQ 223

XP_005233810.1 TKFFLQSSILSQYEVDPERVCVAGDSAGGNLAAAVAQQLLEDTEVETKLKAQALIYPALQ 223

XP_005434343.1 TKFFLQSSILSQYEVDPERVCVAGDSAGGNLAAAVAQQLLEDTEVETKLKAQALIYPALQ 223

XP_003218232.1 TKYFLQRSVLEEHKVDPERVCISGDSAGGNLAAAVTQQLLDDPDVKVKLKIQVLIYPALQ 223

XP_007439720.1 AKYFLQSSILEKYNVEPSRICIAGDSAGGNLAAAVTQKLLDDPDVKVKFKIQALFYPFLQ 223

XP_013910823.1 SKYFLQSSILEKYNVDPSRIGIAGDSAGGGLAAAVTQQLLDDPDVRTKFKIQALLYPGLQ 223

XP_015275105.1 AKYFLQNSVLDQYNVDSSRVCVSGDSAGGNLAAAVAQQLLHDPEVKVKLKIQVLIYPALQ 224


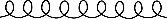

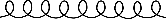

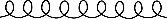

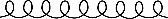


XP_015669807.1 AKYFLQSSILKKYNVDPSRIGIAGDSAGGNLAAAVTQQLLDDPDVKAKFKIQALIYPALQ 223

XP_020637486.1 TKYFLQSNVLDQYKVDPSRVCIAGDSAGGNLAAAVTQQLLHDPDIDVQLKIQALLYPALQ 223

XP_026520917.1 AKYFLQSSILEKYTVDPSRIGIAGDSAGGNLAAAVTQQLLDDPDVNAKFKVQALLYPGLQ 223

XP_026557410.1 AKYFLQSSILEKYTVDPSRIGIAGDSAGGNLAAAVTQQLLDDPDVKAKFKVQALLYPGLQ 223

XP_028587147.1 AKYFLQSSVLEQYNVDASRVCVAGDSAGGNLAAAVAQQVLRDPGVKIKFKIQVLIYPALQ 223

XP_014425448.1 VKFFLQNSVLAQYGVDPHRICVSGDSAGGNLAAAVAQKLLEDLEVKVKLKIQVLLYPALQ 233

XP_021013481.1 LRWFLQKDILEKYGVDPRRVGVSGDSAGGNLAAAVTQQLIQDPDVKIKLKVQALIYPALQ 222

XP_032752628.1 LRWFLQEDILEKYGVDPRRVGVSGDSAGGNLAAAVTQQILQDPDVKIKLKVQALIYPALQ 222

XP_032887718.1 VKYFLQREILAQYMVDSGRVAVAGDSAGGNLAAAVSQQIQEDPKVEVAIKIQVLVYPALQ 223

XP_029884721.1 TKFFLQSSILSQYGVDPDRVCVAGDSAGGNLAAAVAQQLLKDPEVKTKLKAQALLYPALQ 223

XP_034357074.1 LRWFLQEDILEKYGVDPRRVGVSGDSAGGNLAAAVTQQLLQDPDIKNKLKVQVLIYPALQ 222

XP_032048897.1 TKFFLQSSVLSQYGVDPNRVCVAGDSAGGNLAAAVVQKLLEDSEVTTRIRAHVLLYPALQ 223

XP_008498425.2 TKFFLQSSVLSQYGVDPDRVCVSGDSAGGNLAAAVVQQLLEDPEVKTKIKVQALIYPALQ 223

XP_030810306.1 TKFFLQSKVLSQYGVDPARVCVAGDSAGGNLAAAVAQQLSEDPEVKTKLRAQVLIYPALQ 223

XP_006189146.2 LKWFLHPNVLENYGVDPRRVGISGDSAGGNLAAAVTQQLLEDPDVKIKLKVQSLIYPALQ 223

XP_032924696.1 TKFFLQSKVLSQYGVDPARVCVAGDSAGGNLAAAVAQELLEDAEVQTKLKAQVLIYPALQ 223

XP_032630208.1 VKFFLQNSVLAQYGVDPNRICISGDSAGGNLAAAVAQQLLDDLEVKVKLKIQALLYPALQ 223

XP_032554164.1 TKFFLQSSVLARYGVDPARVCVAGDSAGGNLAAAVAQQLLEDPEVKTKLKAQVLLYPALQ 223

XP_006868037.1 LRWFLRKKVLAEYGVNPERIGISGGSAGGNLAAALAQQLLDDPDVKTKLKIQSLIYPALQ 223

XP_031975985.1 TKFFLQSKVLSQYGVDPARVCVAGDSAGGNLAAAVAQQLLDDPEVKTKLKAQILLYPALQ 223

XP_035422447.1 TKFFLQSSVLSQYGVDPNRVCVAGDSAGGNLAAAVAQKLLEDSEVTTRLRAQVLLYPALQ 223

XP_033814578.1 VKYFLKKEVLSEYGVDPNRICIAGDSAGGNLAAAITQQLQDDAEVDVKPKMQALIYPALQ 223

XP_035931406.1 LKWFLRQDVLDKYGVDPGRIGISGESAGGNLVASVTQQLIDDPDIKIKLKTQSLIYPALQ 223

XP_032028132.1 LRWFLRKKVLAKYGVNPERIGISGDSAGGNLAAAVTQQLLDDPDVKIKLKIQSLIYPALQ 223

XP_033006343.1 AKYFLQSSVLERYNVEASRVCVAGDSAGGNLAAAVAQQVLHDPGVKIKFKIQALMYPALQ 223

XP_017934775.3 TKFFLQSSVLAQYGVDPARVCVAGDSAGGNLAAAVAQQLLEDPEVKTKLKAQVLLYPALQ 223

XP_031231873.1 LRWFLQEDILEKYGVDPRRVGVSGDSAGGNLAAAVTQQLIQDPDVKIKLKVQVLIYPALQ 222

XP_005141224.2 TKFFLQSSVLSQYGVDPDRVGVAGDSAGGNLAAAVAQQLLEDPEVKNKLKIQALLYPALQ 223

XP_036244957.1 TKFFLQSKVLSQYGVDPARVCVAGDSAGGNLAAAVAQQLSEDPEVKTKLRAQVLIYPALQ 223

XP_036207699.1 LKWFLRKDILKGYGVDPKRIGISGDSAGGNLAAAVTQQLIDDPDVKIKLKIQSLIYPALQ 223

XP_012365981.2 LRWFLRKKVLAKYGVNPERIGISGDSAGGNLAAAVTQQLLDDPDVKIKLKIQSLIYPALQ 223

XP_036045372.1 LRWFLQEDILETYGVDPRRVGVSGDSAGGNLAAAVTQQLIQDPDVKTKLKVQALVYPALQ 223

XP_008950113.1 LRWFLRKKVLAKYGVNPERIGISGDSAGGNLAAAVTQQLLDDPDVKIKLKIQSLIYPALQ 223

XP_034295103.1 AKYFLQSSILEKYNVDASRIGIAGDSAGGNLAAAVTQQLLDDPEVKAKFKIQALFYPGLQ 223

XP_003894989.2 LRWFLRKTVLAKYGVNPERIGISGDSAGGNLAAAVTQQLLDDPDVKIKLKIQALIYPALQ 223

XP_031459500.1 SKFFLQSRVLSQYAVDPTRVCVAGDSAGGNLAAAVAQKLLEDSEVTNKLKAQALIYPALQ 223

XP_032264231.1 LKWFLRQDVLDKYGVDPGRIGISGESAGGNLVAAVTQQLIDDPDIKIKLKTQSLIYPALQ 223

XP_036301415.1 LKWFLRQDILKGYGVDPKRIGISGDSAGGNLAAAVTQQLIDDPDVKIKLKIQSLIYPALQ 223

XP_029472042.1 AKYFLQKEVLTEYGVDPNRICVAGDSAGGNLAAAVTQQLQDDIEVEVRPKIQALIYPALQ 223

XP_032118892.1 LRWFLRKNVLAKYGVNPERIGISGDSAGGNLAAAVTQQLLDDPDVKIKLKIQALIYPALQ 223

XP_030351736.1 TKFFLQSSVLSQYGVDPDRVCVAGDSAGGNLAAAVAQQLSEDPEVKTKLKVQALLYPALQ 223

XP_030136362.2 TKFFLQSEVLSQYGVDPARVCVAGDSAGGNLAAAVAQQLVEDPEVKTKLRAQLLIYPALQ 223

XP_037380609.1 LRWFLRTKVLAQYGVDPERIAVSGDSAGGNLAAAVTQQLLDDPDVKVKLKIQSLIYPALQ 223

XP_034637747.1 VKFFLQNSVLAQYGVDPNRICISGDSAGGNLAAAVAQQLLDDLEVKVKLKIQALLYPALQ 223

XP_033060172.1 LRWFLRKTVLAKYGVNPERIGISGDSAGGNLAAAVTQQLLDDPDVKIKLKIQSLIYPALQ 223

XP_032842734.1 TKFFLQSSVLSQYGVDPDRVCVAGDSAGGNLAAAVAQQLLEDSEVKTKLKAQALIYPALQ 223

XP_034989473.1 AKYFLQSSVLERYNVEASRVCVAGDSAGGNLAAAVAQQVLHDPDVKIKFKIQALLYPALQ 223

XP_005525205.1 TKFFLQSEVLAQYGVDPARVCVAGDSAGGNLAAAVAQQLSEDPEVKTKLKAQILIYPALQ 223

XP_009277639.1 MKFFLQRSVLSQYGVDPDRVCVAGDSAGGNLAAAVAQQLLEDSEVETKLKAQALLYPVLQ 223

XP_009332236.1 TKFFLQSSVLSQYGVDPDRVCVAGDSAGGNLAAAVAQQLLEDPEVETKLKAQALLYPALQ 223

XP_009507799.1 TKFFLQSSVLSQYGVDPDRVCVAGDSAGGNLAAAVAQQLLEDSEVKTKLKAQALLYPALQ 223

XP_009565977.1 TKFFLQSSVLSQHGVDPDRVCVAGDSAGGNLAAAVAQQLLQDSEVKTKLKAQALIYPALQ 223

XP_009575589.1 TKFFLQSSVLSQYGVDPDRVCVAGDSAGGNLAAAVAQQLLEDSEVKTRLKAQALLYPALQ 223

XP_009632710.1 TKFFLQSSILSQYGVDPDRVCVAGDSAGGNLAAAVAQQLLKDSEVKTKLKAQALLYPALQ 224

XP_009674653.1 TKFFLQSSVLSQYGVDPNRICVAGDSAGGNLAAAVAQQLLDDPEVKTKLKVQALLYPALQ 223

XP_009818640.1 TKFFLQSSVLSQYGVDPDRVCVAGDSAGGNLAAAVAQQLLQDAEVETKLKAQALLYPALQ 223

XP_009882692.1 TKFFLQSSVLSQYGVDPERVCVAGDSAGGNLAAAVAQQLLEDSEVKTKLKAQALLYPALQ 223

XP_009922085.1 TKFFLQSSILSQYGVDPDRVCVAGDSAGGNLAAAVAQQLLKDPDVKTKLKVQALLYPALQ 223

XP_009940690.1 TKFFLQSNVLSQYGVDPDRVCVVGDSAGGNLAAAVAQQLLQDSEVKTKLKAQALLYPALQ 223

XP_010017334.1 TKFFLQSSVLSQYGVDPDRVCVAGDSAGGNLAAAVAQQLLEDPEVKTKLKVQALLYPALQ 223

XP_010157376.1 TKFFLQSSVLSQYGVDPDRVCVAGDSAGGNLAAAVAQQLLEDTEVKTKLKAQALLYPALQ 223

XP_010171133.1 TKFFLQRNILSQYGVDPERVCVAGDSAGGNLAAAVAQQLLEDSEVETKLKAQVLIYPALQ 223


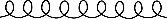

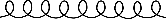

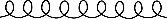

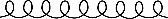


XP_010190764.1 TKFFLQRSVLSRYSVDPHHVGIAGDSAGGNLAAAVAQQLLEDSEVETKLKAQALIYPALQ 223

XP_010296603.1 TKFFLQSSVLSQYGVDPDRVCVAGDSAGGNLAAAVAQQLLEDSEVKTKLKVQALIYPALQ 223

XP_010583207.1 TKFFLQSSILSQYGVDPDRVCVAGDSAGGNLAAAVAQQLLKDPDVKTKLKVQALLYPALQ 223

XP_013054760.1 TKFFLQSSVLSQYGVDPNRVCVAGDSAGGNLAAAVAQKLLEDSEVTTRLRAQVLLYPALQ 223

XP_013797768.1 TKFFLQGSVLSQYGVDPNRICVAGDSAGGNLAAAVAQQLLDDPEVKTKLKVQALLYPALQ 223

XP_014801236.1 TKFFLQSSVLSQYGVDPDRICVAGDSAGGNLAAAVAQQLLKDSEVKTKLKAQALLYPALQ 223

XP_015493839.1 TKFFLQSEVLARFGVDPARVCVAGDSAGGNLAAAVAQQLSEDPEVKTKLKAQILIYPALQ 223

XP_015727689.1 SKFFLQSRVLSQYSVDPTRVCVAGDSAGGNLAAAVAQKLLEDSEVTTKLKAQALIYPALQ 223

XP_017690958.1 TKFFLQSSVLAQYGVDPARVCVAGDSAGGNLAAAVAQQLLEDPEVKTKLKAQVLLYPALQ 223

XP_021251550.1 TKFFLQSSVLSQYGVDPQRVCVAGDSAGGNLAAAVAQQLLEDSEVKTKLKVQALIYPALQ 223

XP_021403179.1 TKFFLQSQVLSQYGVDPARVCVAGDSAGGNLAAAVAQQLVEDPEVKTKLRAQLLIYPALQ 223

XP_023788489.1 TKFFLQSEVLAQYGVDPARVCVAGDSAGGNLAAAVAQQLSEDPEVKTKLKAQILIYPALQ 223

XP_025920950.1 TKFFLQGSVLSQYGVDPNRICVAGDSAGGNLAAAVAQQLLDDPEVKTKLKVQALLYPALQ 223

XP_026711093.1 TKFFLQSSVLSQYGVDPDRVCVAGDSAGGNLAAAVAQQLLADAEVKTKLRAQALIYPALQ 223

XP_027503068.1 TKFFLQSSVLARYGVDPARVCVAGDSAGGNLAAAVAQQLLEDPEVKTKLKAQVLLYPALQ 223

XP_027541513.1 TKFFLQSRVLSQYGVDPTRVCVAGDSAGGNLAAAVAQQLLQDPEVKTKLKAQVLLYPALQ 223

XP_027587074.1 TKFFLQSSVLAQYGVDPARVCVAGDSAGGNLAAAVAQQLLEDPEVKTKLKAQVLLYPALQ 223

XP_027747710.1 TKFFLQSRVLSQYGVDPTRVCVAGDSAGGNLAAAVAQQLLEDAEVKTKLKAQVLLYPALQ 223

::: .* . :. :: : * *:*..*.* * * : : *.** :*


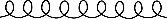

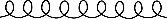

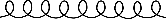

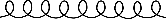

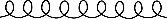

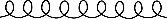


NP_001077.2 PLDVDLPSYQENSNFLFLSKSLMVRFWSEYFTTDR-SLEKAMLSRQHVPVESSHLFKFVN 282

NP_065413.1 ALDMNVPSQQENSQYPLLTRSLLIRFWSEYFTTDR-DLEKAMLLNQHVPVEFSHLLQFVN 281

NP_075872.1 ALDTNVPSQQEGSHFPVLTRSLMVRFWSEYFTTDR-GLEKAMLLNQHVPMESSHLLQFVN 281

XP_001145851.1 PLDVDLPSYQENSNFLFLSKSLMVRFWSEYFTTDR-SLEKAMLSRQHVPVESSHLFKFVN 282

XP_534309.2 TLDMDLPSYRENSHFPILPKSLMVRFWSEYFTVDK-SLVKAMFSNQHVPVESSHLYKFIN 282

XP_001106694.1 PLDVDLPSYQENSDFLILSKSLMVRFWSEYFTTDR-SLEKAMLSRQHVPVESSHLFKFVN 282

XP_028929988.1 SLDMDLPSYRENEHMPILKKSFMVRLWSEYFTTDR-ALEEAMRSNRHVPVDSSHLFKFVN 282

XP_019652071.1 TLDTDLPSYRECSHFPTLSRSMTVRMWSEYFTTDR-SLEKAMLFNQHVPVESSHLFKFVN 282

XP_003416197.1 PLDVDLPSYQENAHFPVLPKSSMVRLWSDYFTTDR-SLERAMLSNQHIPLESSHLLQFVN 282

XP_003511717.1 ALDMNLPSYQEGLYFPILSPLLMTRFWSEYFTTDR-TLEKAMLLNQHVPMESSHLFQFVN 281

XP_003925073.1 PLDVDSPSYQENSHFPFLSKSLMVRFWSEYFTTDR-SLEKAMLSRQHVPVESSHLFKFVN 282

XP_004037911.1 PLDVDLPSYQENSNFLFLSKSLMVRFWSEYFTTDR-SLEKAMLSRQHVPVESSHLFKFVN 282

XP_004682395.1 PLDVDSPSYRENSEFPFLSRSLMIRFWSEYLTADPASLEKAMLSNQHVPAESHHLLKFVN 283

XP_005077976.1 ALDMNLPSYQEGSDFPILTPSTMIRFWSEYFTTDR-TLEKAMHLNQHVPMESSHLFQFVN 281

XP_005344092.1 ALDINLPSYQEGSYFPLLTPSVMARFWSEYFTTDR-TLEKAMLLNQHVPLESSHLLQFVN 283

XP_006099319.1 TLDMDLPSLRENSQIPPLPKSLMVRFWSEYFTTDR-SLEKAMFVNQHVPVESSHLFKFVN 282

XP_006202876.1 NFDMDLPSYRENSHFPILSKSLMVRFWSEYFTTDR-SLEKAMLSNQHIPLESIHQFKFVN 282

XP_006733329.1 TLDMDLPSYRECSRFPTLSKSMMVRLWSGYFTEDR-SLAKAMLFNQHVPVESRHLFKFVN 282

XP_006771196.1 TLDMDLPSLRENSQIPPLPKSLMVRFWSEYFTTDR-SLEKAMFINQHVPVESSYLFKFVN 282

XP_006901609.1 TLDMDLPSYRENAFSFFLPKWIMVRFWSEYITTDR-SLEKAMLSKQHVPVEASHLFNFVN 282

XP_006908288.1 ILDMDTPSYQENSNFLPF-RRLIARLRSEYLSIDR-SLEKTIFSNQIVPVESSHLLKFVN 281

XP_006994722.1 ALDLKLPSYQEGSNFPFLTTSLMGRLWSEYFSTDR-TLEKAMLLNQHVPMEFSHLFQYVN 281

XP_007529626.1 TLDVNSPSYKEDTNFLVLSKSLMIRFWSEYFTTDR-SLEKAMLSNQHVPLESSHLFKFVN 282

XP_008006884.1 PLDVDLPSYQENSNFLLLSKSLMVRFWSEYFTTDR-SLEKAMLSRQHVPVESSHLFKFVN 282

XP_008584385.1 TLDVDSPSYRENSHFPVLPKSLMVRFWSEYFTTDR-SLEEAMLSNQHVPVESSHLFQFVN 282

XP_008843029.1 ALDLDLPSYRENSHGPILSRSVMVRFWSEYFTTDQ-SLEKSMLFNQHVPMESTHLFKFVN 282

XP_010370405.1 PLDVDLPSYQENSNAPVLSKSLMVRFWSEYFTTDR-SLEKAMLSRQHVPVESSHLFKFVN 282

XP_010950295.1 NFDMDLPSYRENSHFPILSKSLMVRFWSEYFTTDR-SLEKAMLSNQHIPLESIHQFKLVN 282

XP_010984995.1 NFDMDLPSYRENSHFPILSKSLMVRFWSEYFTTDR-SLEKAMLSNQHIPLESIHQFKLVN 282

XP_011814358.1 PLDVDLPSYQENSNVLLLSKSLMVRFWSEYFTTDR-SLEKAMLSRQHVPVESSHLFKFVN 285

XP_011828136.1 PLDVDLPSYQENSNFLLLSKSLMVRFWSEYFTTDR-SLEKAMLSRQHVPVESSHLFKFVN 282

XP_012292585.1 PLDVDSPSYQENSHSPFLQKSFMVRFWSEYFTTDR-SLEKAMLSRQHVPVESSHLFKFVN 282

XP_012646746.1 TLDMDLPSYREYPNFPLLPRSLMVRFWSEYFTTDR-SLEKAMLFNQHIPVESSHLFKFVN 282

XP_012889095.1 TLNMNLPSYQDHADLPFLTRSLMIKFWSSYFTTDR-SLEKAMLFNQHIPMELSHLFQFVN 282

XP_014705074.1 PLDVDTPSYQGYSHFPVLSKSLMVRFWSEYFTTDR-SLEKAMLSHQHVPVESSHLFKFVN 282

XP_016078067.1 ALDVDTPSYREHSHFPLLFKSVMVRHWSEYFTTDR-SLEKAMLSHQHVPVESSHLFKFVN 282

XP_017710441.1 PLDVDLPSYQENSNAPVLSKSLMVRFWSEYFTTDR-SLEKAMLSRQHVPVESSHLFKFVN 282

XP_020039411.1 ALDMDLPSYRENAYFPSLSTSVMVRFWSEYFTTDR-SLEEAMLSNQHVPMESIHLFKFVN 282

XP_021052676.1 ALDTNVPSYQEGSHFPMLTRSLMVRFWSEYFTTDQ-GLEKAMLLNEHVPMESSHLLQFVN 281

XP_021516657.1 ALDLNLPSFQENSHFPFLSRSLMVRLWSEYFTTDQ-ALEKAMLVNQHVPMEHSHLFRFVN 281

XP_023087853.1 PLDVDLPSYQENSNVLLLSKSLMVRFWSEYFTTDR-SLEKAMLSRQHVPVESSHLFKFVN 282


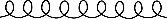

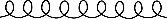

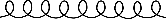

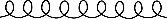

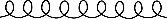


XP_024415983.1 TLDMDLPSYRENSLLPTLPRSLMVRFWSEYFTTDR-SLEKAMLFNQHVPVESVHLFQFVN 282

XP_025231688.1 PLDVDLPSYQENSNFLLLSKSLMVRFWSEYFTTDR-SLEKAMLSRQHVPVESSHLFQFVN 282

XP_025291926.1 TLDMDLPSYRENSHFPILPKSLMVRFWSEYFTVDK-SLVKAMFSNQHVPVESSHLYKFIN 282

XP_025871547.1 TLDMDLPSYRENSHFPFLPKSLMVRFWSEYFTVDK-SLIKAMFSNQHVPVESSHLYKFIN 282

XP_026263974.1 TLDVETPSYQDNAHMPVLPKSLAIRFYSNYITSDS-ALRKAMMSNQHVPVEAAPLFKLVN 276

XP_027789579.1 TLDMETPSYQDNAHMPVLPKSLTIWFYSNYITSDS-ALRKAMKRNQHVPVEAAPLFKLVN 276

XP_028619475.1 ALDMNVPSLQEGAHFPLLTRSLMVRFWSEYFTTDK-DLEKAMLLNQHVPMEFSHLLQLVN 281

XP_028746441.1 ALDMKLPSYQEGSNFPFLTTSLMGRLWSEYFTTDR-TLEKAMLLNQHVPMEFSHLLQFVN 281

XP_006034098.1 TLDLNLPSYQDNEDKPILPKSLMVRFWSEYFTNDT-SLNEAMASNRHVPEESSHLFAFVN 282

XP_019358692.1 TLDLDLPSYQDNEDKPILPKSLMVRFWSEYFTNDT-SLNEAMASNSHVPEESSHLFAFVN 282

XP_019388142.1 TLDLDLPSYQDNEDKPILPKSLMVRFWSEYFTNDT-SLNEAMASNRHVPEESSHLFAFVN 282

XP_018426679.1 TLDLYTPSYRENGNMPLLSRTLMVRFWSEYFTTDQ-KLFKAMLSNRYIPSQEAHLFKFVN 282

XP_004943624.1 TLDLNLPSYHQNADMPVLSKSLMVRFWSEYFTSDP-SLREAITSNRHVPAQWGHLFQFVN 282

XP_003209327.1 ILDLNLPSYQQNADMPILSKSLMIRFWSEYFTSDP-SLREAMTSNRHVPAQWGHLFQFVN 282

XP_005023727.2 TLDLNLPSYQQNENGLILPKSLMVRFWSEYFTSDS-SLREAMTSNSHVPAESGHLFQFVN 282

XP_005233810.1 TLDLNLPSYQENKNKLILPKSLMVKFWSEYFTSDS-SLREAMASNRHVPVESSHLFQFVN 282

XP_005434343.1 TLDLNLPSYQENKNKLILPKSLMVKFWSEYFTSDS-SLREAMASNRHVPVESSHLFQFVN 282

XP_003218232.1 TIDMNLPSYQDNKNMPILPKSLMLRFWSEYFTTDL-SLMEAIETNQYVPPEFSHLFKFVN 282

XP_007439720.1 TVDLELPSYQDNKNMPVLTKSLVIRFLSEYISSDD-SLFKVLETNQHVPAEFNHLFKFVN 282

XP_013910823.1 SIDLELPSYQDNKNIPILSKTLVIRFLSEYITSDE-SFSKALETNQHVPAEFNHLFKFVN 282

XP_015275105.1 TLDMDLPSYRDNENKPILPKSLMVRFWSEYFTNDT-SLKAAMETNQHVPVEWSGLFKFVN 283

XP_015669807.1 SIDMELPSYQDNKNMPILPKGILFRFFSEYITSDK-SFAKALETNQHVPAEFNHLFKFVN 282

XP_020637486.1 TLDLDLPSYRDNKDMPILPKSLMLRFLSEYFTTDL-SLMEAMETNQHVPPEASNLLKFVN 282

XP_026520917.1 IIDVELPSYQDNKNMPILPKALIIRFFSEYIKTDD-SFLKALETNQHVPVEYNHLFTFVN 282

XP_026557410.1 IIDVELPSYQDNKNMPILPKALMIRFFSEYIVTDD-SFLKALETNQHVPVEYNHLFTFVN 282

XP_028587147.1 TIDLDLPSYRDNANMLILPKSLMVRFWSEYFTTDM-SLNRAMEANQHVPAELSHLFKFAN 282

XP_014425448.1 TLDLDSPSYRDNENKPILPRSLMVRFWSEYFTTDI-SLKEAMASNRHVPAESSHLFRLVN 292

XP_021013481.1 ALDTNVPSYQEGSHFPVLTRSLMVRFWSEYFTTDR-GLEKAMNLNQHVPMESSHLLQFVN 281

XP_032752628.1 ALDMNVPSQQENSQYPLLTRSLLIRFWSEYFITDR-DLEKAMLLNQHVPVEFSHLLQFVN 281

XP_032887718.1 TIDFNTPSYQQNGNMPILPKTLMVRFWSEYFSRDK-LLLKDMKDNTHTGREDRDLNSLVN 282

XP_029884721.1 TLDLNLPSYRENENKPVLPRLLMVKFWSEYFTSDS-SLREAMASNRHVPVESSHLFQFVN 282

XP_034357074.1 ALDMNVPSLQEDAHFLLLTKSLMVRFWSEYFTTDQ-DLEKAMLLNQHVPMEFSHLFQFVN 281

XP_032048897.1 TLDLNLPSYQQNENGLILPKSLMVRFWSEYFTTDS-SLREAMTSNRHVPAESGHLFQFVN 282

XP_008498425.2 TLDLNLPSYQDNDNKPILPRSLMIRFWGEYFTSDP-SLQEAMATNSHVPLESSHLLQFVN 282

XP_030810306.1 ALDLDLPSYRDNAHKPLLPRSLMVRFWSEYLSPEP-ALRAAMAANRHAPPEAGRLLPLVN 282

XP_006189146.2 NFDMDLPSYRENSHFPILSKSLMVRFWSEYFTTDR-SLEKAMLSNQHIPLESIHQFKLVN 282

XP_032924696.1 ALDLALPSYQDNAHKPILSRSLMVRFWSEYFSSDP-ALREAMASNTHVPPEASALWPLLN 282

XP_032630208.1 TLDLDLPSYKDNENKPILPKSLMVRFWSEYFTTDV-SLQEAMASNRHVPAESSHLFRFVN 282

XP_032554164.1 ALDLDLPSYRDNANKPLLSRSLMVRFWSEYFTSDP-SLREAMASNRHVPAEAGHLLHFVN 282

XP_006868037.1 PLDLDTPSYQENSHFPFLSKSFMVRFWSEYFTTDR-SLEKAMLSNQHIPEESSHLFKFVN 282

XP_031975985.1 ALDLNLPSYRDNDNKPLLPRSLMVRFWSEYFTSDP-ALREAMASNRHVPADSGHLFQFVN 282

XP_035422447.1 TLDLNLPSYQQNENGLILPKSLMVRFWSEYFTSDS-SLREAMASNRHVPAESGHLFQFVN 282

XP_033814578.1 ALNLDTPSYRDNAHMPILTKSLMAKFWSEYFTTDK-DLFNAMHSNSLQPSEASHLFSFIN 282

XP_035931406.1 TLDMDLPSYRECSHFPTLSKSMMVRLWSEYFTEDR-SLAKAMLFNQHVPVESRHLFKFVN 282

XP_032028132.1 PLDVDLPSYQENSNFLFLSKSLMVRFWSEYFTTDR-SLEKAMLSRQHVPVESSHLFKFVN 282

XP_033006343.1 TIDLDLPSYRDNGNMPILPKSLMVRFWSEYFTTDM-SLNRAMEANQHVPAEQSHLFKFAN 282

XP_017934775.3 ALDLDLPSYRDNANKPLLSRSLMVRFWSEYFTSDP-SLREAMASNRHVPAEAGHLLQFVN 282

XP_031231873.1 ALDLNSPSYQEGLHFPILTRSLMVRFWREYFTTDQ-GLEKAMLLNQHVPMEFSHLLQFVN 281

XP_005141224.2 ALDLNLPSYRENENKPILSKSLIIRFWSGYFTSDL-SLREAMTSNRHVPVESSHLFQFVN 282

XP_036244957.1 ALDLDLPSYRDNAHKPLLSRALMVRFWSEYLSADP-ALRAAMASNRHVPPEAGPLLPLVD 282

XP_036207699.1 TLDMDLPSLRENSQIPPLSKSLMVRFWSEYFTTDR-SLEKAMSINQHVPVESSYLFKFVN 282

XP_012365981.2 PLDVDLPSYQENSNFLFLSKSLTVRFWSEYFTTDR-SLEKAMLSRRHVPVESSHLFKFVN 282

XP_036045372.1 ALDMKLPSHQEGSYFPFLPTSLLIRLWSEYFTTDR-TLEKAMLLNQHVPMEFSHLFQFVN 282

XP_008950113.1 PLDVDLPSYQENSNFLFLSKSLMVRFWSEYFTTDR-SLEKAMLSRQHVPVESSHLFKFVN 282

XP_034295103.1 TIDLELPSYQDNKNMPILPKTLLIRFLSEYITSDE-SFFNALETNQHVHAEFNHLFKFVN 282

XP_003894989.2 PLDVDLPSYQENSNFLLLSKSLMVRFWSEYFTTDR-SLEKAMLSRQHVPVESSHLFKFVN 282

XP_031459500.1 TLDLNLPSYQQNANMPILSKSLMIRFWSEYFTSDP-SLREAMTSNRHVPAQWGHLFQFVN 282

XP_032264231.1 TLDMDLPSYRECSHFPTLSKSMMVRLWSEYFTEDR-SLAKAMLFNQHVPVESRHLFKFVN 282

XP_036301415.1 SLDMDLPSYRENAHFSTLPRSLMVRFWSEYITTDR-SLEKAMLVNQHVPVESSYLFKFVN 282

XP_029472042.1 PLDLDTPSYRDNSHMPILSKSLMVRFWSEYFTVDK-ALFNAMSSNTHKLIESNHLLRFIN 282

XP_032118892.1 PLDVDSPSYQENAHFPFLHKSLMVRFWSEYFTTDR-SLEKAMLSRQHVPVESSHLFKFVN 282

XP_030351736.1 VLDLNLPSYQENENDPILSRSLTVRFWSEYFTSDL-SLREAMISNRHVPVESSHLFQFVN 282


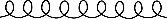

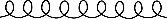

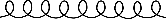

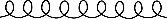

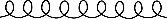


XP_030136362.2 VLDLDLPSYRDNAEKPLLPRSLMVRFWSEYFTSDP-ALREAMASNRHVPAEAAQLLPLVN 282

XP_037380609.1 PLDVDSPSYRENSHFPLLSRSSMVRFWREYFTTDP-SLEKAMLSNQHVPVESHHLLKFVN 282

XP_034637747.1 TLDLDLPSYKDNENKPILPKSLMVRFWSEYFTTDV-SLQEAMASNSHVPAESSHLFRLVN 282

XP_033060172.1 PLDVDLPSYQENSNAPVLSKSLMVRFWSEYFTTDR-SLEKAMLSRQHVPVESSHLFKFVN 282

XP_032842734.1 ALDLNLPSYQANENKPLLSKLLMARFWSEYFASDP-SLWEAIASNRHVPAEASHLFQFVN 282

XP_034989473.1 TIDLDLPSYRDNGNMPILPKPLMVRFWSEYFTTDM-SLNRAMEANQHVPAEQSHLFKFAN 282

XP_005525205.1 ALDLNLPSYRDNADKPILSRSLMVRFWSEYFTSDP-ALREAMDSNRHVPAESSHLFQFVN 282

XP_009277639.1 SLDLNLPSYQENENKPVLSKLYMVRLWSEYFTSDS-SLREAMASNRHVPVESSHLFPFVN 282

XP_009332236.1 SLDLNLPSYQENENKPVLSKSYMVRLWSEYFTSDS-SLREAMASNRHVPVESSHLFPFVN 282

XP_009507799.1 TLDLNLPSYQDNENKPILPKSLMVKFWSEYFTSDS-SLREAMASNRHVPVESSHLFQFVN 282

XP_009565977.1 TLDLNLPSYRDNANKPLLPRSFMVRFWSVYFTLDP-SLREAMASNRHVPVEASHLFQFVN 282

XP_009575589.1 TLDLNLPSYRENENKPILPRLFMVKFWSEYFTSDS-SLREAMASNRHVPVESSHLFQFVN 282

XP_009632710.1 TLDLNLPSYQENENKPILLKSLMVKFWSEYFTSDP-SLREAMASNRHVPVESSHLFQFVN 283

XP_009674653.1 TLDLNLPSYQENENKLILPKSLMVRFWSEYFTTDS-SLREAMASNRHVPAEASHLFQFVN 282

XP_009818640.1 TLDLNLPSYRENENKPILSRLLMVRFWSEYFTSDS-SLREAMASNSYVPVESSHLFQFVN 282

XP_009882692.1 TLDLNLPSYQQNENKPILTKPLMAKFWSEYFTSDS-SLTEAMATNRHVPIEWSHLFQFVN 282

XP_009922085.1 TLDLNLPSYRENENKPILPRLLMVKFWSEYFTSDS-SLREAMASNRHVPVESSHLFQFVN 282

XP_009940690.1 TLDLNLPAYQDNENKPLLSKSLMVRFWSEYFTSDP-SLREAMASNTHIPVESSHLFQFVN 282

XP_010017334.1 TLDLNLPSYQENENKPILSKSLMVRFWSEYFTSDL-SLREAMISNRHVPVESSHLFQFVN 282

XP_010157376.1 ALDLNLPSYQKNENKPILPRSLMVKFWSEYFTSDP-SLREAMASNRHVPVESSHLFQFVN 282

XP_010171133.1 SLDLNLPSYQENADQVILSRSLTVKFWSEYFTSDL-ALRDAMASNRHVPLESSHLFQFVN 282

XP_010190764.1 TLDLNLPSYQENKHSPILSRLLMVRFWSEYFTSDP-SLRQAMASNAHVPVESSHLFQFVN 282

XP_010296603.1 ALDLNLPSYRQNENKPILPKLLMVKFWSEYFTSDL-SLREAMASNRHVPLESSHLFQFVN 282

XP_010583207.1 TLDLNLPSYRENENKPILPRLLMVKFWSEYFTSDS-SLREAMASNRHVPVESSHLFQFVN 282

XP_013054760.1 TLDLNLPSYQENENGLILPKSLMVRFWSEYFTSDS-SLREAMASNRHVPAESGHLFQFVN 282

XP_013797768.1 TLDLNLPSYQDNENKPILSKSLMVRFWSEYFTTDS-SLREAMASNRHVPAESSHLFQFVN 282

XP_014801236.1 TLDLNLPAYQENEHKPILPKSLMVKFWSEYFTSDS-SLREAMASNRHVPVESSHLFQFVN 282

XP_015493839.1 ALDLNLPSYRDNADKPILSRSLMVRFWSEYFTSEP-ALREAMDSNRHVPPESSQLLQFVN 282

XP_015727689.1 TLDMNLPSYHQNADMPILSKSLMIRFWSEYFTSDP-SLREAMTSNRHVPAQWGHLFQFVN 282

XP_017690958.1 ALDLDLPSYRDNANKPLLSRLLMVRFWSEYFTSDP-SLREAMASNRHVPAEAGHLLQFVN 282

XP_021251550.1 TLDLNLPSYQQNQNMPILSKSLMVRFWSEYFTSDP-SLREAMSSNRHVPAKWGHLFQFVN 282

XP_021403179.1 VLDLDLPSYRDNAEKPMLPRSLMVRFWSEYFASDP-ALREAMASNRHVPAEAAQLLPLVN 282

XP_023788489.1 ALDLNLPSYRDNADKPILSRSLMVRFWSEYFTSEP-ALREAMDSNRHVPAESSHLFQFVN 282

XP_025920950.1 TLDLNLPSYQDNENKPILSKSLMVRFWSEYFTTDS-SLREAMASNRHVPAESSHLFQFVN 282

XP_026711093.1 PLDLNLPSYQENANKPILSKSLMVRFWSEYFTSDS-SLREAMALNRHVPVEYSHLFQFVN 282

XP_027503068.1 ALDLDLPSYRDNTDKPLLSRSLMVRFWSEYFTSDP-SLREAMASNSHVPAEVGHLLQFVN 282

XP_027541513.1 ALDLDLPSYRDNADKPLLSGPLMVRFWSEYFTSDP-ALREAMASNRHVPAEAGHLFQFVN 282

XP_027587074.1 ALDLDLPSYRDNANKPLLSRSLMIRFWSEYFTSDP-SLREAMASNRHVPAEAGHLLQFVN 282

XP_027747710.1 TLDLDLPSYRDGDNKPLLPRRLMVRFWSEYFTSDP-SLREAMASNRHVPAESGHLFQFVN 282

.: *: : : *: : : : . . :


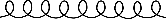

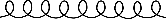

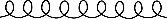


NP_001077.2 WSSLLPERFIKGHVYNN-PNYGS-SELAKKYPGFLDVRAAPLLADDNKLRGLPLTY---- 336

NP_065413.1 WSSLLPQRYKKGYFYKT-PTPGS-LELAQKYPGFTDVKACPLLANDSILHHLPMTY---- 335

NP_075872.1 WSSLLPERYKKSPVYKN-PTPGS-SELAQKYPGFIDVKACPLLANDNILHHLPKTY---- 335

XP_001145851.1 WSSLLPERFIKGHVYNN-PNYGS-SELAKKYPGFLDVRAAPLLADDNKLRGLPLTY---- 336

XP_534309.2 WSSLLPEKFKKGYFYKS-PTYGS-SELAKKYPGFLDVRAAPLLADDNKLRSLPLTY---- 336

XP_001106694.1 WSSLLPERFIKGHVYNN-PNYGS-SELAKKYPGFLDVRAAPLLADDKKLHSLPLTY---- 336

XP_028929988.1 WSALLPEKFKKGHIYNS-PIPGT-PDLAKKYPGYLDVRASPLLAADAKLRRLPLTY---- 336

XP_019652071.1 WSSLLPEKFKKGHFYNS-PTYGS-SELAKKYPGFLDVRAAPLLADDNKLRGLPLTY---- 336

XP_003416197.1 WSSLLPERFKKGHVYKN-PIYGS-SELSNKYPGFLDVRAAPLLADDNKLRDLPRTY---- 336

XP_003511717.1 WSSLLPERYKKGYFYKS-PTPGS-SELSKKYPGFLDVKACPLLANDNILSQLPRTY---- 335

XP_003925073.1 WSSLLPERFIKGYVYSN-PNYGS-SELAKKYPGFLDVRAAPLLADDNRLHGLPLTY---- 336

XP_004037911.1 WSSLLPERFIKGHVYNN-PNYGS-SELAKKYPGFLDVRAAPLLADDNKLHGLPLTY---- 336

XP_004682395.1 WSSLLPERYIKGNVYKD-PTYGS-SELAMKYPGLLDVRVAPLLADDKKLHRLPLTY---- 337

XP_005077976.1 WSSLLPERYTEGHLYKS-PTPGS-SELSRKYPGFLDVKACPLLANDNTLSHLPPTY---- 335

XP_005344092.1 WSSLLPERYKKGRVYKS-PTPGS-SELSKKYPGFLDVKACPLLANDNTLSHLPLTY---- 337

XP_006099319.1 WSSLLPENFKKGHIYSS-PTHGP-SELARKYSGFLDVRAAPLLADDNKLRNLPLTY---- 336

XP_006202876.1 WSSLLPEKFKKGHIYKT-PTHGS-SELAKKYPGFLDVKASPLLADDNKLRNLPLTY---- 336

XP_006733329.1 WSSLLPEKFKKGHFYNS-PTYGS-SELTKKYPGFLDVRAAPLLADDNKLRSLPLTY---- 336

XP_006771196.1 WSSLLPENFKKGHFYNS-PIHGP-SELARKYPGFLDVRAAPLLADDNKLRNLPLTY---- 336

XP_006901609.1 WSSLLPEKFKKSHFYNS-PTAGP-SQLAKKYPGFLDVRAAPLLADDSKLRGLPPTY---- 336


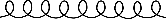

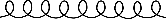

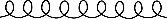


XP_006908288.1 WSSLLPKRFIKGHVYNN-RTYGS-SELLKKYPDLLDVRVSPLLADDKKLRSLPQTY---- 335

XP_006994722.1 WSSLLPERYKTGHFYES-PTPGS-SELAKKYPGFLDVKACPLLANDNTLSHLPLTY---- 335

XP_007529626.1 WSSLLPERFTKGYVYNN-PTFGG-SELSKKYPGFLDVRAAPLLADDSKLRGLPMTY---- 336

XP_008006884.1 WSSLLPERFIKGHVYNN-PNYGS-SELAKKYPGFLDVRAAPLLADDKKLHSLPLTY---- 336

XP_008584385.1 WSSLLPEKFKKGHIYKN-PTHGS-SELAKKYPGFLDVRAAPLLADDKKLRGLPLTY---- 336

XP_008843029.1 WSSWLPERFKKGHIYKN-PVPGS-SELGKKYPGFLDVRASPLLADDNILRHLPLTY---- 336

XP_010370405.1 WSSLLPERFIKGHVYNN-PNYGS-SELAKKYPGFLDVRAAPLLADDKKLHSLPLTY---- 336

XP_010950295.1 WSSLLPEKFKKGHIYKT-PTHGS-SELAKKYPGFLDVKASPLLADDNKLRNLPLTY---- 336

XP_010984995.1 WSSLLPEKFKKGHIYKT-PTHGS-SELAKKYPGFLDVKASPLLADDNKLRNLPLTY---- 336

XP_011814358.1 WSSLLPERFIKGHVYNN-PNYGS-SELAKKYPGFLDVRAAPLLADDKKLHSLPLTY---- 339

XP_011828136.1 WSSLLPERFIKGHVYNN-PNYGS-SELAKKYPGFLDVRAAPLLADDKKLHSLPLTY---- 336

XP_012292585.1 WSSLLPERFIKGYVYSN-PNYGS-SELAKKYPGFLDVRAAPLLADDSKLHGSPLTY---- 336

XP_012646746.1 WSSLLPEKFRKGHVYNN-PTHGS-SKLAKKYPGFLDVRAAPLLADDNKLRGLPVTY---- 336

XP_012889095.1 WSSLLPERFVKGHIYRH-PIPGS-SELVKKYPGLLDVRAAPLLADESKLRGLPLAY---- 336

XP_014705074.1 WSSLLPERFLKGYVYNN-PIYGS-SELSKKYPGYLDVRAAPLLADDHKLHGLPLTY---- 336

XP_016078067.1 WSSLLPERFIKGHVYNN-PTYGK-SELSKKYPGFLDVRAAPLLADDKKLRGLPRTH---- 336

XP_017710441.1 WSSLLPERFIKGHVYNN-PNYGS-SELAKKYPGFLDVRAAPLLADDKKLHSLPLTY---- 336

XP_020039411.1 WSSLLPERFKNGHFYNS-PSTGS-SELAKKYPGFLDVRASPLLAEDSKLRGLPLTY---- 336

XP_021052676.1 WSSLLPERYKKSHIYKN-PTPGS-SELAQKYPGFIDVKACPLLANDNILRQLPLTY---- 335

XP_021516657.1 WSSLLPERYKKGYFYKS-PTPGS-SELAQQYPGFVDVKACPLLADDNILLHLPLTY---- 335

XP_023087853.1 WSSLLPERFIKGHVYNN-PNYGS-SELAKKYPGFLDVRAAPLLADDKKLHSLPLTY---- 336

XP_024415983.1 WSSLLPEIFKKGHFYSS-PTNGT-SELFKKYPGFLDERAAPLLADDNKLQGLPVTY---- 336

XP_025231688.1 WSSLLPERFIKGHVYNN-PNYGS-SELAKKYPGFLDVRAAPLLADDKKLHSLPLTY---- 336

XP_025291926.1 WSSLLPEKFKKGYFYKS-PTYGS-SELAKKYPGFLDVRAAPLLADDNKLRSLPLTY---- 336

XP_025871547.1 WSSLLPEKFKKGYFYKS-PTYGS-SELAKKYPGFLDVRAAPLLADDNKLRSLPLTY---- 336

XP_026263974.1 WSSLLPERFLKGHVYKA-PTFGS-SELARKHPGFLDVRVAPLLAADSKLRDLPLTY---- 330

XP_027789579.1 WSSLLPERFLKGHVYKA-PTFGR-SELARKHPGFLDVRVAPLLAADSKLRDLPRTY---- 330

XP_028619475.1 WSSLLPERYKKGHFYKN-PSPGS-SELAQKYPGFIDVKACPLLANDNILHHLPLTY---- 335

XP_028746441.1 WSSLLPDRYKTGHFYES-PTPGS-SELAKKYPGFLDVKACPLLANDNTLSHLPLTY---- 335

XP_006034098.1 WSNWLPEKFKKDHTYKG-PIYGS-SKFEEKYPGFLDPRAAPLLADNVKLHGLPLTY---- 336

XP_019358692.1 WSNWLPEKFKKDHTYKG-PIYGS-SKFGEKYPGFLDPRAAPLLADNVKLQGLPLTY---- 336

XP_019388142.1 WSNWLPEKFKKDHTYKG-PIYGS-SKFGEKYPGFLDPRAAPLLADNVKLQGLPLTY---- 336

XP_018426679.1 WSTLLPNSLKNNHVYH-KPEYGD-SSFVKKYPGILDIKSSPLLQEDENLKGLPLTY---- 336

XP_004943624.1 WSTLLPDRMKKGHVYTG-PVFGS-PALAKKYPGFLDRRAAPLLAAEAQLRGLPPTY---- 336

XP_003209327.1 WSTLLPDRLKKGHVYTG-PAFGS-PALAEKYPGFLDPRAAPLLAAEARLRGLPPTY---- 336

XP_005023727.2 WSNLLPEEMKKDYVYTS-PVFGS-SELAKKYPGFLDPRAAPLLAEDARLRGLPPTY---- 336

XP_005233810.1 WSNLLPEELKKDHVYSS-PTYGS-SELAQKYPGFLDRRAAPLLVSDAQLHRLPLTY---- 336

XP_005434343.1 WSNLLPEELKKDHVYSS-PTYGS-SELAQKYPGFLDRRAAPLLVSDAQLHRLPLTY---- 336

XP_003218232.1 WSEWLPERFKRDHVYTS-PVPGK-SKFGQKYPGFFDPRAAPLLVDDVKLRGLPLTY---- 336

XP_007439720.1 WSHWLPERFKKGHIYTA-PSHGN-SKIGQKYPGILDPRTAPLLVDDIKLQGLPLTY---- 336

XP_013910823.1 WSKWLPERFKKGHIYTD-PSHRN-SKTGHKYPDLLDPRSGPLLVEDIKLQGLPLTY---- 336

XP_015275105.1 WSNWLPEEFKKGHIYTS-PSHGS-SKVGKKYPGLLDPRAAPLLADNTKLQGLPLTY---- 337

XP_015669807.1 WSNWLPERFKKGHIYTD-PSHRI-SKIGHKYPGLVDPRLAPLLVDEIKLRGLPLTY---- 336

XP_020637486.1 WSNWLPERFKKGHIYTI-PKHEN-SKIGQKYPGLLDPRAAPLLVDDAKLHGLPLTY---- 336

XP_026520917.1 WSNWLPERFKKGHVYTD-PSHRN-SKIGLKYQDFVDPRVAPLLVEDIKLRGLPLTY---- 336

XP_026557410.1 WSNWLPERFKKGHVYTD-PSHRN-SKIGPKYQDFVDPRIAPLLVEDIKLRGLPLTY---- 336

XP_028587147.1 WSHWLPERFKKGHVYTK-PVYGN-SELGKKYPGFLDPRAAPLLVDESKLRGLPLTY---- 336

XP_014425448.1 WSNLLPEEFKKDHVYVG-PKYGS-SRFGKKYPGFLDPRAAPLLADDTKLLGLPMTY---- 346

XP_021013481.1 WSSLLPERYKKSPVYKN-PTPGS-SELAQKYPGFIDVKACPLLANDNILHHLPKTY---- 335

XP_032752628.1 WSSLLPQRYKKGYFYKT-PPPGS-SELAQKYPGFTDVKACPLLANDSILHHLPMTY---- 335

XP_032887718.1 WSILLPEKFRKQYKYSP-PGQGNEGTSSTIVPGIFDPRAAPLLAPDEKLRALPRAF---- 337

XP_029884721.1 WSNLLPEELKKDHVYTS-PTYGS-SELAEKYPGFLDPRAAPLLASDAQLRGLPLTY---- 336

XP_034357074.1 WSSLLPERYKKGHFYKN-PSPGS-SELAQKYPGFIDVKACPLLANDNILHHLPPTY---- 335

XP_032048897.1 WSNLLPEEMKKDYVYTS-PVFGS-SKLAKKYPGFLDPRAAPLLAEDARLHGLPPTY---- 336

XP_008498425.2 WSKLLPEEMKRGHVYRA-PVPGS-PGLARRYPGFLDVRAAPLLAPDARLLRLPHTY---- 336

XP_030810306.1 WSRWLPAAMRGARTPTR-ARPCARWRGAGGAPALLDRV-RAAAGAAGALRCRPRRS---- 336

XP_006189146.2 WSSLLPEKFKKGHIYKT-PTHGS-SELAKKYPGFLDVKASPLLADDNKLRNLPLTY---- 336

XP_032924696.1 WSLWLPPDMRGAHRPSS-PVWGG-AELARRHPGLLDPRASPLLASQARLRGLPPAF---- 336

XP_032630208.1 WSNLLPEKFKKDHVYAS-PIYGS-SMLGKKYPGFLDPRAAPLLVDDTKLLGLPVTY---- 336

XP_032554164.1 WSRLLPAGMRQGHVYAA-PPAGS-AALARRYPGFLDPRAAPLLGSDARLRQLPPTY---- 336

XP_006868037.1 WSSLLPERFKKGYVYKN-PTYGS-SELSNKYPGFLDVRAAPLLADDNKLRHLPLTY---- 336

XP_031975985.1 WSRLLPAEMRKAHEYGG-PAVGG-AGLAHGHRGFLDPRASPLLASDARLHRLPPAY---- 336


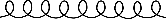

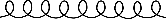

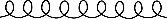


XP_035422447.1 WSNLLPEEMKKDYVYTS-PVFGS-SKIAKKYPGFLDPRAAPLLAEDTRLHGLPPTY---- 336

XP_033814578.1 WPALLPKEMQKAHTYNHSPQSRM-LEFAKKYPGILDPRAAPLLVEDAKLARLPQTY---- 337

XP_035931406.1 WSSLLPEKFKKGHVYNS-PTYGS-SELAKKYPGFLDVRAAPLLADDNKLRSLPLTY---- 336

XP_032028132.1 WSSLLPERFIKGHVYND-PNYGS-SELAKKYPGFLDVRAAPLLADDNKLHGLPLTY---- 336

XP_033006343.1 WSHWLPEKYKKGHVYTK-PVYGN-SELGQKYPGFLDPRAAPLLVDESKLRGLPLTY---- 336

XP_017934775.3 WSRLLPAGMREGHTYAA-PTPGS-AALARRYPGFLDPRAAPLLGNSEWR--LPPSCPRLH 338

XP_031231873.1 WSSLLPARYKKNHVYKN-PTPGS-SELAQKYPGFIDVKACPLLASDNILHHLPLTY---- 335

XP_005141224.2 WSNLLPEEMKKDHVYVS-PTYGS-SELAQKYPGFLDLRASPLLANDAQLQKLPRTY---- 336

XP_036244957.1 WSRWLPAAMRGAHTYRG-PALGA-AGPARRFPGLLDPRACPLLASEARLRRLPPAF---- 336

XP_036207699.1 WSSLLPENFKKGHFYSS-PTHGP-SELARKYSGFLDVRAAPLLADDNKLRNLPLTY---- 336

XP_012365981.2 WSSLLPERFIKGHVYND-PNYGS-SELAKKYPGFLDVRAAPLLADDNKLHGLPLTY---- 336

XP_036045372.1 WSSLLPERYKTDHFYEN-PTPGS-NELAKKYPGFLDVKACPLLANDNILSHLPLTY---- 336

XP_008950113.1 WSSLLPERFIKGHVYNN-PNYGS-SELAKKYPGFLDVRAAPLLADDNKLRGLPLTY---- 336

XP_034295103.1 WSNWLPERFKKGHVYTD-PSHRN-FKIGHKYPDLLDPRMAPLLIEDIKLRGLPLTY---- 336

XP_003894989.2 WSSLLPERFIKGHVYNN-PNYGS-SELAKKYPGFLDVRAAPLLADDKKLHSLPLTY---- 336

XP_031459500.1 WSTLLPDRLKKDHVYTG-PVFGS-PALAEKYPGFLDPRAAPLLATEAQLQGLPPTY---- 336

XP_032264231.1 WSSLLPEKFKKGHVYNS-PTYGS-SELAKKYPGFLDVRAAPLLADDNKLRSLPLTY---- 336

XP_036301415.1 WSSLLPENFKKGHIYSN-PTHGP-SELARKYSGFLDVRAAPLLVDDNKLRSLPLTY---- 336

XP_029472042.1 WTDLLPEKMKKIHTYSNI-QSGT-SEFTKKYPGILDPRAAPLLVDDAKLSRLPQTY---- 336

XP_032118892.1 WSSLLPERFIKGYVYSN-PNYGS-SELAKKYPGFLDVRAAPLLADDNKLHGLPLTY---- 336

XP_030351736.1 WSNLLPEEMKKDHIYIS-PTYGS-SKLAQKYPGFLDLRAAPLLAGDAQLRKLPRTY---- 336

XP_030136362.2 WSRWLPAEMRRAHAYAR-AAPGS-AELARRYPGLLDPRASPLLASEARLRRLPRTF---- 336

XP_037380609.1 WSSLLPERYIKGHVYKN-PIYGS-SEIAVKYPGLFDVRAAPLLADDKKLRSLPLTY---- 336

XP_034637747.1 WSNLLPEKFKKDHVYAS-PIYGS-SRLGKKYPGFLDPRAAPLLVDDTKLLGLPVTY---- 336

XP_033060172.1 WSSLLPERFIKGHVYNN-PNYGS-SELAKKYPGFLDVRAAPLLADDKKLHSLPLTY---- 336

XP_032842734.1 WSNLLPEELKKDHVYAG-PSYGS-SELVQKYPGLLDVRAAPLLASDARLRGLPLSY---- 336

XP_034989473.1 WSRWLPERYKKGHVYTK-PVYGS-SELGQKYPGFLDPRAAPLLVDESKLRGLPLTY---- 336

XP_005525205.1 WSRWLPAEMRKAHAYGG-PTPGG-AELARRFPGFLDPRASPLLASEARLRHVPPAY---- 336

XP_009277639.1 WSNLLPEELKKGHVYTS-PTYGS-SELAQKYPGFLDPRAAPLLASDAWLRRLPLTY---- 336

XP_009332236.1 WSNLLPEELKKDHVYTS-PTYGN-SELAQKYPGFLDPRAAPLLASDARLRRLPLTY---- 336

XP_009507799.1 WSNLLPEELKKGHVYTS-PAYGS-SELAQKYPGFLDPRAAPLLASDGQLRGLPLTY---- 336

XP_009565977.1 WSNLLPEELKKDHVYTT-PTYGS-SELAQKYPGFLDLRAAPLLASDAQLRELPLTY---- 336

XP_009575589.1 WSNFLPEELKKGHVYTS-PAYGS-SELAQKYPGFLDPRAAPLLVSDAQLRGLPLTY---- 336

XP_009632710.1 WSNLLPEELKKDHVYTS-PTYGS-SELAQKYPGFLDPRAAPLLVSDDQLRRLPLTY---- 337

XP_009674653.1 WSNLLPEELKKDHVYTS-PVYGS-SNLGKKYPGFLDPRAAPLLAEDAKLHSLPLTY---- 336

XP_009818640.1 WSNLLPEEVKKDHIYTS-PTYGS-SELAQKYPGFLDPRAAPLLASDAQLRGLPLTY---- 336

XP_009882692.1 WSHLLPEELKKDYVYTG-PTYGS-SEFAQKYPGFLDTRAAPLLASDAQLRGLPLTY---- 336

XP_009922085.1 WSNLLPEELKKDYVYTS-PTYGS-SELAEKYPGFLDPRAAPLLASDAQLRGLPLTY---- 336

XP_009940690.1 WSNWLPEELKKDHVYTS-PVYGS-SELVGKYPGLLDRRAAPLLASDAQLHGLPLSY---- 336

XP_010017334.1 WSNLLPEEMKKDHVYSS-PTYGN-SKLVQKYPGFLDPRAAPLLAGDAQLHKLPRTY---- 336

XP_010157376.1 WSTLLPEELKKGHVYAH-PAYGS-PELAQKYPGFLDARAAPLLTSDARLRGLPLTY---- 336

XP_010171133.1 WSQFLPEELKKGHVYTG-PVYGS-SELAQKYPGFLDPRAAPLLVSDARLRRLPLTY---- 336

XP_010190764.1 WSHLLPEEMKKDHVYTG-PVYSS-PALAQKYPGFLDPRAAPLLASDARLHGLPLTY---- 336

XP_010296603.1 WSNLLPEELKKDHVYTS-PTYGS-PELARKYPGFLDLRAAPLLASDAQLRGLPLTY---- 336

XP_010583207.1 WSNLLPEELKKDYVYTS-PTYGS-SELAEKYPGFLDPRAAPLLASDAQLRGLPLTY---- 336

XP_013054760.1 WSNLLPEEMKKDYVYTS-PVFGS-SKIAKKYPGFLDPRAAPLLAEDTRLHGLPPTY---- 336

XP_013797768.1 WSNLLPEELKKDHVYTS-PIYES-SNLGKKYPGFLDPRAAPLLAEDAKLRGLPLTY---- 336

XP_014801236.1 WSNLLPEELKKDHVYTS-PTYGS-SELAQKYPGFLDTRAAPLLARDAQLRGLPLAY---- 336

XP_015493839.1 WSRWLPAEMRKAHAYGA-PAPGG-SELARRFPGFLDPRASPLLASEARLLHVPPAY---- 336

XP_015727689.1 WSTLLPDRMKKDHVYTD-PVYGS-PALAKKYPGFLDLRAAPLLAAEAQLRALPPTY---- 336

XP_017690958.1 WSRLLPAGMRKGHVYAA-PRPGS-AALARRYPGFLDPRAAPLLGSDARLRQLPPTY---- 336

XP_021251550.1 WSALLPDQLKKDHVYTS-PVFGS-PALAKKYPGFLDPRAAPLLAAEAQLRGLPPTY---- 336

XP_021403179.1 WSRWLPAEMRGAHAYAS-PAVGD-TELARRYPGLLDPRASPLLAGEARLRRLPRTL---- 336

XP_023788489.1 WSRWLPAEMRKAHAYGG-PTPGG-AELARRFPGFLDPRASPLLASEARLRHVPPAY---- 336

XP_025920950.1 WSNLLPEELKKDHVYTS-PIYES-SNLGKKYPGFLDPRAAPLLAEDAKLRGLPLTY---- 336

XP_026711093.1 WSALLPAELKKGHAYTS-PTFGS-PELAHKYPGFLDPRAAPLLGSDERLRGLPPTY---- 336

XP_027503068.1 WSRLLPAGMRRGHVYAA-PTPGS-AALARRYPGFLDPRAAPLLGSDARLRQLPPTY---- 336

XP_027541513.1 WSRLLPAGMRRGHEYVT-PSPGS-AELAQRYPGFLDPRASPLLVSDALLRQLPPTY---- 336

XP_027587074.1 WSRLLPAGMRQGHAYAA-PTPGS-AALARRYPGFLDPRAAPLLGSDARLRQLPPTY---- 336

XP_027747710.1 WSQLLPAGMRKGLAYTA-PTYGG-AGLARRYPGFLDPRAAPLLASDARLRHLPRTY---- 336

* ** * *


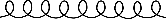

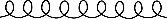

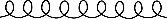


NP_001077.2 VITCQYDLLRDDGLMYVT-RLRNTGVQVTHNHVEDGFHGAFSF----LGLKISHRLINQY 391

NP_065413.1 IITCQYDVLRDDGLMYVK-RLQNTGVHVTHHHIEDGFHGALTL----PGLKITYRMQNQY 390

NP_075872.1 IITCQYDVLRDDGLMYVK-RLQNVGVHVTHHHVEDGFHGTFSF----PGLKLSERMKNQY 390

XP_001145851.1 VITCQYDLLRDDGLMYVT-RLRNAGVQVTHNHVEDGFHGAFSF----LGLKISHRLINQY 391

XP_534309.2 VITCQYDVLRDDGIMYVT-RLQNAGVQVTHNHIEDGFHGAFSF----SGFKIADRLENQY 391

XP_001106694.1 VITCQYDPLRDDGLMYVT-RLRNAGVQVTHNHVEDGFHGAFSI----LELKISHRLINQY 391

XP_028929988.1 IMTCQHDVLRDDGLMYVS-RLRQAGVQVVHEHIENGFHGILSFISFPTDLNVGHRVGNEY 395

XP_019652071.1 VITCQYDVLRDDGIMYVT-RLQNAGVPVIHNHIEDGFHGAFSY----YGLKIGYRIENQY 391

XP_003416197.1 VITCQYDVLRDDGLMYVT-RLRNAGVQVTHNHAEAGFHGAFGF----LELKISSRMISQY 391

XP_003511717.1 IITCQYDVLRDDGLMYAM-RLQNAGVHVTHHHVENGFHGAFTL----HGFKISYRLQNQY 390

XP_003925073.1 VVTCQYDVLRDDGLMYVT-RLRNTGVQVTHNHVENGFHGVFSF----PELKIHHRLINQY 391

XP_004037911.1 VITCQYDLLRDDGLMYVT-RLRNAGVQVTHNHVEDGFHGAFSF----LGLKISHRLINQY 391

XP_004682395.1 IITCQYDVLRDDGLMYVT-RLRNAGVPVTHNHIENGFHGIFSF----LEFKISDKIINNY 392

XP_005077976.1 IITCQYDVLRDDGIMYVR-RLQNAGVQVTHHHIENGFHGAFTL----HGFKIAYRLQNQY 390

XP_005344092.1 IITCQYDVLRDDGLMYVT-RLQNVGVQVTHHHIENGFHGAFTL----HGYKIAHKMQNQY 392

XP_006099319.1 VITCQYDVLRDDGIMYVT-RLRNAGVRVTHNHIEGGFHGVMTF----PGFKIGHRVENQY 391

XP_006202876.1 IITCQYDVLRDDGLMYVT-RLRNSGVQVVHNHVEGAFHGTVSF----LFMKIGYRLANQY 391

XP_006733329.1 VITSQYDVLRDDGIMYVT-RLQNAGVPVTHNHIEDGFHAAFSY----YGFKIGYRIENQY 391

XP_006771196.1 VITCQYDVLRDDGIMYVT-RLRNAGVRVTHNHVEGGFHGLISI----PGFKIGLKVENQY 391

XP_006901609.1 VLTCQYDVLRDDGVMYVT-RLKNAGVQVTHNHADDGFHGALYL----PNFKIGERLKNHY 391

XP_006908288.1 IVTCQHDIFRDDGLMYVT-RLQNAGVSVTHNHIENGIHGLLSL----SGLKITHEMKNQY 390

XP_006994722.1 IITCQYDVLRDDGLMYVR-RLQNVGVHVTHHHFEDGFHGAFTL----HRFKIADKMQNQY 390

XP_007529626.1 ILTCQYDVLRDDGVMYVT-RLRNAGVRVTHNHIEDGFHGTLSF----RELKIGSRTENQY 391

XP_008006884.1 VITCQYDPLRDDGLMYVT-RLRNAGVQVTHNHVEDGFHGAFSI----LELKISHRLINQY 391

XP_008584385.1 VITCQYDVLRDDGLMYVT-RLRNAGVQVTHNHIEGAFHGTLSF----LQFNIGHRTVNQY 391

XP_008843029.1 IITCQYDVLRDDGLMYVT-RLQNAGVQVTLDHIENGFHGAFSL----HKLKISNRLENKY 391

XP_010370405.1 VITCQYDPLRDDGIMYVT-RLRNAGVQVTHNHVEDGFHGAFSF----LEFKISHRLINQY 391

XP_010950295.1 IITCQYDVLRDDGLMYVT-RLRNSGVQVVHSHVEGAFHGTVSF----LFMKIGYRLANQY 391

XP_010984995.1 IITCQYDVLRDDGLMYVT-RLRNSGVQVVHSHVEGAFHGTVSF----LFMKIGYRLANQY 391

XP_011814358.1 VITCQYDPLRDDGLMYVT-RLRNAGVQVTHNHVEDGFHGAFSF----LELKISHRLINQY 394

XP_011828136.1 VITCQYDPLRDDGLMYVT-RLRNAGVQVTHNHVEDGFHGAFSI----LELKISHRLINQY 391

XP_012292585.1 VVTCQYDVLRDDGLMYVT-RLRNAGVQVTHNHVEDGFHGVFSF----LELKISHRLINQY 391

XP_012646746.1 VITCQYDVLRDEGIMYVT-RLRNAGVQVTHNHVEDGYHGALSY----NGFKIEYRIENQY 391

XP_012889095.1 IITCQYDILRDDGLLYVA-RLRSAGVNVTHDHMEDGFHGLLAY----IWLKIKDRTENQY 391

XP_014705074.1 IITCQYDVLRDDGLMYVS-RLRNAGVRVTHNHVEDGFHGSFSL----LNFKIGYRLINQY 391

XP_016078067.1 VITCEYDVLRDDGIMYVT-RLQNAGVPVTHNHIEDGFHGSFLL----LKFKISSRMINQY 391

XP_017710441.1 VITCQYDPLRDDGIMYVT-RLRNAGVQVTHNHVEDGFHGAFSF----LEFKISHRLINQY 391

XP_020039411.1 IITCQYDVLRDDGVMYVT-RLQNAGVQVTHNHIEDGFHGAFSY----NDMKIFYRMENQY 391

XP_021052676.1 IITCQYDVLRDDGLMYVK-RLQNVGVHVTHHHIEDGFHGAFAF----PGLRLSERMQNQY 390

XP_021516657.1 IITCQYDVLRDDGLMYVT-RLQNVGVHVTHHHIEDGFHGAFTF----HDFKITYRMQNQY 390

XP_023087853.1 VITCQYDPLRDDGLMYVT-RLRNAGVQVTHNHVEDGFHGAFSF----LELKISHRLINQY 391

XP_024415983.1 VITCQYDVLRDDGIMYVT-RLQKAGVRVTHQHIEDGFHGAISY----GGFKIGYRIQNHY 391

XP_025231688.1 VITCQYDPLRDDGLMYVT-RLRNAGVQVTHNHVEDGFHGAFSI----LELKISHRLINQY 391

XP_025291926.1 VITCQYDVLRDDGIMYVT-RLQNAGVQVTHNHIEDGFHGAFSF----SGFKIADRLENQY 391

XP_025871547.1 VITCQYDVLRDDGIMYVT-RLQNAGVQVTHNHIEDGFHGAFSF----SGFKIADRLENQY 391

XP_026263974.1 LVTCQYDVVRDDGLMYVR-RLRDAGVPVTHNHLEDGFHGILSFPV--LSFKIYYRLVDEY 387

XP_027789579.1 LVTCQYDVLRDDGLMYVR-RLRDAGVPVTHNHVEDGLHGILSFPV----FKIYYRLMDEY 385

XP_028619475.1 IITCQYDVLRDDGLMYVK-RLQNAGVHVTHHHIEDGFHGAFTL----FGLKIANRMQNQY 390

XP_028746441.1 IITCQYDVLRDDGLMYVT-RLQNVGVHVTHHHFEDGFHGAFTL----HHFKIADKIQNQY 390

XP_006034098.1 VLTCQHDVLRDDGIMYVS-RLREAGIQVTHDHIEDAFHGALMFITSPTDLALGHRLANKY 395

XP_019358692.1 VLTCQHDVLRDDGIMYVS-RLREAGIQVTHDHIEDAFHGALMFITSPTNLALGHRLANKY 395

XP_019388142.1 VLTCQHDVLRDDGIMYVS-RLREAGIQVTHDHIEDAFHGALMFITSPTDLALGHRLANKY 395

XP_018426679.1 VLTCMYDVLRDDGFMYAS-RLRQAGVQVVHHHYDSTFHGILLLNTLPFDFAIAHHISDKY 395

XP_004943624.1 ILTCEHDVLRDDGVMYAT-RLKAAGVPVTHEHAEDGFHGALIFVTSPADMAVGHRLLGRC 395

XP_003209327.1 ILTCQHDVLRDDGFMYAS-RLRAAGVPVTHEHAEDGFHGALTFVTSPADMAVGHRLMGGY 395

XP_005023727.2 VLTCEYDVLRDDGVMYVS-RLRAAGVRVTHDHAKDAFHGALMFVSSPANLPVGNRLRNRY 395

XP_005233810.1 ILTCEHDVLRDDGVMYAR-RLQAAGVPVTHDHIKDAFHGAIMFVSGPAELAVGHRLLNRY 395

XP_005434343.1 ILTCEHDVLRDDGVMYAR-RLQAAGVPVTHDHIKDAFHGAIMFVSGPAELAVGHRLLNRY 395

XP_003218232.1 VITCQHDVLRDDGLMYVS-RLRKVGVPVIHEHLEGAIHGMLLMNSGPLFLNGGQIAVNNY 395

XP_007439720.1 VITCQHDVLRDDGIIYVS-RLREAGVPVIHEHVENAVHGVLTFIAGPVILNVGHRMANNY 395

XP_013910823.1 IMTCQYDVLRDDGIMYVS-RLREAGVEVTHEHVDNAFHGVVTFITGPFAQNIGHRMANNY 395

XP_015275105.1 VITCQHDVLRDDGIMYVS-RLREAGVPVVHKHVEDAVHGALTFIMSPFNLSVGQRMANNY 396


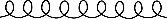

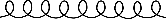

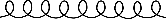


XP_015669807.1 VITCQYDILRDDGIIYVF-RLREAGVQVIHEHVDNTIHGPLTFITGPFALNIGHRIANNY 395

XP_020637486.1 VLTCQYDVLRDDGIMYVS-RLREAGVPVIHEHVEDAVHGILLFTTSPFILTAGERVVNNY 395

XP_026520917.1 ILTCQYDILRDDGIIYAS-RLREAGVQVAHEHADNAFHGSMTFLSGPFALNTGHRMANNY 395

XP_026557410.1 ILTCQYDILRDDGIIYAS-RLREAGVQVAHEHFDDAFHGSMTFLSGPFALNTGHRIANNY 395

XP_028587147.1 VITCQYDVLRDDGLMYVS-RLREAGVNVIHQH-ENTVHGAVMFSTGPLILTVGEKMANDY 394

XP_014425448.1 VLTCQHDVLRDDGLMYVS-RLREAGIEVIHDHIEGAIHGVLMFITSPTHLALGHRVANKY 405

XP_021013481.1 IITCQYDVLRDDGLMYVK-RLQNVGVHVTHHHVEDGFHGAFAF----PGLKLSERMQNQY 390

XP_032752628.1 IITCQYDVLRDDGLMYVK-RLQNTGVHVTHHHIEDGFHGALTL----PGLKITYRMQNQY 390

XP_032887718.1 IMTCEFDVLRDDGVMYAT-RLRGAGVHVTLEHFEDCFHGVLMFITWPTNFAIGQKLMGRY 396

XP_029884721.1 VLTCEHDVLRDDGVMYAG-RLQAAGVPVTHDHAKDAFHGVMMFVLGPAKLAVGYRLLNRY 395

XP_034357074.1 IITCQYDVLRDDGIMYVK-RLQNVGVHVTHHHIEDGFHGAFTF----PGLKIANRIQNQY 390

XP_032048897.1 VLTCEYDVLRDDGVMYVS-RLRAAGVRVTHDHAKDAFHGALMFVSSPTSLPVGNRLRNRY 395

XP_008498425.2 VLTCQHDVLRDDGLMYVK-RLRALGVPVTHHHAQDAFHGAMMFISWPMELAVGHRLLQQY 395

XP_030810306.1 VLTCEHDVLRDDGAMYAAPRLRAAGVPVTHHHAGDAFHGAMTFLAWPLELAVGHRLFNTC 396

XP_006189146.2 IITCQYDVLRDDGLMYVT-RLRNSGVQVVHSHVEGAFHGTVSF----LFMKIGYRLANQY 391

XP_032924696.1 VLTCEHDVLRDDGVMYAA-RLRDAGVPVTHHHAKDGFHGAVTFLTWPLELALGHRLFNTC 395

XP_032630208.1 VLTCQHDVLRDDGIMYVS-RLREVGIEVIHDHIEDAIHGVLMFITSPTDLALGHRVANRY 395

XP_032554164.1 ILTCEHDVLRDDGAMYAQ-RLRDAGVSVTHDHVRDAFHGALLFTSWPFELALGHRLFNRY 395

XP_006868037.1 LITCQYDVLRDDGLMYVT-RLRNAGVQVTHYHVEEGFHGAFSQ----LESKISYRMINQY 391

XP_031975985.1 ILTCEHDVLRDDGVMYAQ-RLRAAGVPVTHHHAKDAFHGAMMFVLWPLELAVGHRLFSTC 395

XP_035422447.1 VLTCEYDVLRDDGVMYVS-RLRAAGVRVTHDHAKDAFHGALMFVSSPTSLPVGNRLRKRY 395

XP_033814578.1 IMTCMYDVLRDDGIIYAQ-RLRKAGVEVTHDHYD-TFHGVLLFLSFPTDLSIAKTMADRY 395

XP_035931406.1 VITCQYDVLRDDGIMYVT-RLQNAGVPVTHNHIEDGFHGAFSY----YGFKIGYRIENQY 391

XP_032028132.1 VITCQYDLLRDDGLMYVT-RLRNAGVQVTHNHVEDGFHGAFSY----LGLKISHRLINQY 391

XP_033006343.1 VITCQYDVLRDDGLMYVS-RLREAGVTVIHQH-ENTLHGVAMFSAGPIILTVGEKMANDY 394

XP_017934775.3 PDLRDDVPLRDDGAMYAQ-RLRNAGVSVTHDHARDAFHGALLFILWPYELAVGHRLCNRY 397

XP_031231873.1 IITCQYDVLRDDGLMYVK-RLQNVGAHVTHHHIEDGFHGAFTS----PGLKITNRLQSQY 390

XP_005141224.2 ILTCQHDVLRDDGFMYAR-RLQRAGVPVTHDHAKDAFHGAITFVSFPAELAVGHRLFNRY 395

XP_036244957.1 VLTCEHDVLRDDGAMYAA-RLRAAGVPVTHHHAKDAFHGAMTFLAWPLELAVGQRLFNTC 395

XP_036207699.1 VITCQYDVLRDDGIMYVT-RLRNAGVRVTHNHVEGGFHGLITF----PGFKIGHRVESQY 391

XP_012365981.2 VITCQYDPLRDDGLMYVT-RLRNAGVQVTHNHVEDGFHGAFSY----LGLKISHRLINQY 391

XP_036045372.1 ITTCQYDVLRDDGLMYVT-RLQNVGVHVTHHHFEDGFHGAFTL----HGFKIADKIQNQY 391

XP_008950113.1 VITCQYDLLRDDGLMYVT-RLRNAGVQVTHNHVEDGFHGAFSF----LGLKISHRLINQY 391

XP_034295103.1 VITCQYDVLRDDGIIYVS-RLRKAGVQVTHEHVDNAVHGVITFLTGPFALNIGQRMANNY 395

XP_003894989.2 VITCQYDPLRDDGLMYVT-RLRNAGVQVTHNHVEDGFHGAFSI----LELKISHRLINQY 391

XP_031459500.1 ILTCQHDVLRDDGFLYAT-RLRALGVPVTHEHAEDGFHGALIFVTSPGDLAVGHRLLSGY 395

XP_032264231.1 VITCQYDVLRDDGIMYVT-RLQNAGVPVTHNHIEDGFHGAFSY----YGFKIGYRIENQY 391

XP_036301415.1 VITCQYDVLRDDGIMYVT-RLRDAGVRVTHNHVEDGFHGIVTL----PGFKIGPRVENQY 391

XP_029472042.1 ILTCMYDVLRDDGVMYAQ-RLKKAGVQVTHDHYD-TFHGVLLFVSSPANLSIAHKMRNEY 394

XP_032118892.1 VVTCQYDVLRDDGLMYVT-RLRNAGVQVTHNHVEDGFHGAFSF----LELKISHRLIKQY 391

XP_030351736.1 ILTCEHDVLRDDGVMYAR-RLQAAGIPVTHDHAKDAFHGAMAFISFPTELAVGRRLLNRY 395

XP_030136362.2 ILTCEHDVLRDDGAMYAV-RLREAGVPVTHLHAKDAFHGAMTFLAWPLELALGHRLLSAC 395

XP_037380609.1 VITCQYDVLRDDGLMYVT-RLRDAGVPVIHKHIENGFHGAFSF----LEFKISHRLINDY 391

XP_034637747.1 VLTCQHDVLRDDGIMYVS-RLREVGIEVIHDHIEDAIHGALMFITSPTDLALGHRAANKY 395

XP_033060172.1 VITCQYDPLRDDGLMYVT-RLRNAGVQVTHNHVEDGFHGAFAF----LEFKISHRLINQY 391

XP_032842734.1 ILTCEHDVLRDDGAMYAE-RLRAAGVPVTHDHAKDAFHGAMTFVLSPVNLAVGHRLLNRY 395

XP_034989473.1 VITCQYDVLRDDGLMYVS-RLREAGVTVKHQH-ENTVHGVAMFSAGPLILTVGEKMANDY 394

XP_005525205.1 ILTCEHDVLRDDGVMYAG-RLRTAGVPVTHHHAKDAFHGAVTFLVWPFKLAVGHRLFDAC 395

XP_009277639.1 ILTCEHDVLRDDGVMYAR-RLRAAGVPVTHDHAKDAFHGAMLFVLGPIELAVGHRLVNRY 395

XP_009332236.1 ILTCEHDVLRDDGVMYAR-RLQAAGVPVTHDHAKDAFHGTMLFVLGPIELAVGHRLVNRY 395

XP_009507799.1 VLTCEHDVLRDDGVMYAR-RLQAVGVPVTHDHAKDAFHGAMMFVLGPTELAVGHRLLNRY 395

XP_009565977.1 ILTCEHDVLRDDGFMYAA-RLRAAGVPVTHDHAKDTFHGAMSFISPPADLVAGHRLVNRY 395

XP_009575589.1 ILTCGHDVLRDDGVMYAR-RLQAAGVPVTHDHAKDAFHGAMIFVSSPAELAVGHRLMNRY 395

XP_009632710.1 VLTCEHDVLRDDGVMYAR-RLQAAGVSVTHDHAKDAFHGVMMFVSGPTKLAVGHRLLNRY 396

XP_009674653.1 VLTCEHDVLRDDGIMYVS-RLREAGVKVTHNHAEDAFHGALAFISSPANLAVGHRLLNHY 395

XP_009818640.1 ILTCEHDVLRDDGVMYAT-RLRAAGVPVTHDNAKDAFHGVMMFASGPVELAIGHRLLNRY 395

XP_009882692.1 ILTCEHDVLRDDGVMYAR-RLQAAGVPVTHDHAKDAFHGAVMFVISPTDLAVGHRLLNRY 395

XP_009922085.1 VLTCEHDVLRDDGVMYAG-RLQAAGVPVTHDHAKDAFHGVMMFVLGPANLAVGYRLLNRY 395

XP_009940690.1 VLTCEHDVLRDDGVMYAR-RMRAVGVPVTHVHAKDAFHGAMMFVSGAAELAVGHRLLNGY 395

XP_010017334.1 ILTCEHDVLRDDGVMYAR-RLQAAGIPVTHDHAKDAFHGAMMFVSFPAELAVGHRLLNRY 395

XP_010157376.1 ILTCEHDVLRDDGVMYAE-RLRAAGVPVAHDHAKDAFHGAMMFVSSPAELAVGHRLLNGY 395

XP_010171133.1 ILTCQYDVLRDDGAMYAQ-RLRAAGVPVTHDHAKDAFHGAMTFISAPTDLAVGHRLANRY 395


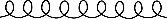

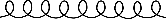

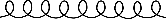


XP_010190764.1 VLTCEHDVLRDDGVMYVQ-RLRAAGIPVVHHHAKDAFHGAMMFVSGLTELAVGHQLANGY 395

XP_010296603.1 LLTCEHDVLRDDGVMYAR-RLQAAGVPIVHDHAKDAFHGVMMFVLSPVNLAVGHRLLNRY 395

XP_010583207.1 VLTCEHDVLRDDGVMYAG-RLQAAGVPVTHDHAKDAFHGVMMFVLGPANLAVGYRLLNRY 395

XP_013054760.1 VLTCEYDVLRDDGVMYVS-RLRAAGVRVTHDHAKDAFHGALMFVSSPASLPVGNRLRNRY 395

XP_013797768.1 VLTCEHDVLRDDGIMYVS-RLREAGVKVTHNHAEDAFHGALMFVSSPGELAVGHRLSNQY 395

XP_014801236.1 VLTCEHDVLRDDGVMYAE-RLRAAGVPVTHDHAKDAFHGAMMFVLSPAELAVGHRLLNRY 395

XP_015493839.1 ILTCEHDVLRDDGTMYAG-RLRAAGVPVTHHHAKDAFHGALTFLVWPFRLAVGHRLFDTC 395

XP_015727689.1 ILTCEHDVLRDDGVMYAT-RLRAAGVPVSHEHAEDGFHGALTFTASPTDMPVGLRLMNNY 395

XP_017690958.1 ILTCEHDVLRDDGAMYAQ-RLRNAGVSVTHDHAQDAFHGALLFISWPYELAVGHRLFNRY 395

XP_021251550.1 ILTCEHDVLRDDGVMYAT-RLRAAGVPLTHEHAEDGFHGALIFVSSPNDMPVGHRLMSRY 395

XP_021403179.1 VLTCEHDVLRDDGAMYAG-RLRQAGVPVTHLHAKDAFHGAVTFLAGPLELAVAHRLLDAC 395

XP_023788489.1 ILTCEHDVLRDDGIMYAE-RLRATGVPVTHHHAKDAFHGAVTFLLWPFKLAVGHRLFDAC 395

XP_025920950.1 VLTCEHDVLRDDGIMYVS-RLREAGVKVTHNHAEDAFHGALMFVSSPGELAVGHRLSNQY 395

XP_026711093.1 VLTCQHDVLWDDGAMYVG-RLRALGVPVTHQHAADAFHGAMTFVLGPAKLAVGQRLLSSY 395

XP_027503068.1 ILTCEHDVLRDDGAMYAR-RLRDAGVSVTHDHARDAFHGALLFTSWPFDLALGHRLFNRY 395

XP_027541513.1 ILTCEHDVLRDDGVMYAR-RLRDAGVSVTHDHAQDAFHGALMFVSWPFELAVGHRLFDRF 395

XP_027587074.1 ILTCEHDVLRDDGAMYAQ-RLRNAGVSVTHDHARDAFHGALLFISWPYELAVGHRLFDRY 395

XP_027747710.1 ILTCEHDVLRDDGAMYAQ-RLRAAGVTVTHDHTQDAFHGAIMFTLWPFELAVAHRLFNSY 395

. *:* :*. *:: * : : *.


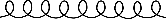


NP_001077.2 IEWLKENL---- 399

NP_065413.1 LNWLHKNL---- 398

NP_075872.1 LSWLIKNL---- 398

XP_001145851.1 IEWLKENL---- 399

XP_534309.2 MNWLSENL---- 399

XP_001106694.1 IEWLKENL---- 399

XP_028929988.1 NSWLKENL---- 403

XP_019652071.1 MNWLSENL---- 399

XP_003416197.1 ISWLSENL---- 399

XP_003511717.1 FSWLMENL---- 398

XP_003925073.1 IEWLKENL---- 399

XP_004037911.1 IEWLKENL---- 399

XP_004682395.1 ISWLHENL---- 400

XP_005077976.1 FSWLMENL---- 398

XP_005344092.1 LSWLMKNL---- 400

XP_006099319.1 LSWLSENL---- 399

XP_006202876.1 ISWLDENL---- 399

XP_006733329.1 MNWLSEHL---- 399

XP_006771196.1 LSWLSENL---- 399

XP_006901609.1 INWLSENL---- 399

XP_006908288.1 LSWLRENL---- 398

XP_006994722.1 LSWLMKNL---- 398

XP_007529626.1 ISWLSDNL---- 399

XP_008006884.1 IEWLKENL---- 399

XP_008584385.1 ISWLHTNL---- 399

XP_008843029.1 MRWLMENL---- 399

XP_010370405.1 IEWLKENL---- 399

XP_010950295.1 ISWLDENL---- 399

XP_010984995.1 ISWLDENL---- 399

XP_011814358.1 IEWLKENL---- 402

XP_011828136.1 IEWLKENL---- 399

XP_012292585.1 IEWLKENL---- 399

XP_012646746.1 MSWLHENL---- 399

XP_012889095.1 LSWLRQNL---- 399

XP_014705074.1 ISWLSENL---- 399

XP_016078067.1 ISWLSEYL---- 399

XP_017710441.1 IEWLKENL---- 399

XP_020039411.1 ISWLRENL---- 399

XP_021052676.1 LSWLIKNL---- 398

XP_021516657.1 LSWLMKNL---- 398

XP_023087853.1 IEWLKENL---- 399


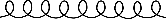


XP_024415983.1 LSWLNENL---- 399

XP_025231688.1 IEWLKENL---- 399

XP_025291926.1 MNWLSENL---- 399

XP_025871547.1 MNWLSENL---- 399

XP_026263974.1 IGWLDENL---- 395

XP_027789579.1 IRWLDENL---- 393

XP_028619475.1 LNWLIKNL---- 398

XP_028746441.1 LSWLMKNL---- 398

XP_006034098.1 IAWLDENL---- 403

XP_019358692.1 IAWLDLNL---- 403

XP_019388142.1 IAWLDMNL---- 403

XP_018426679.1 LMWLDSNL---- 403

XP_004943624.1 LCCPALLPRSLN 407

XP_003209327.1 LQWLEEHL---- 403

XP_005023727.2 IAWLNENL---- 403

XP_005233810.1 IEWLNENL---- 403

XP_005434343.1 IEWLNENL---- 403

XP_003218232.1 IEWLNKNL---- 403

XP_007439720.1 IEWLNKNL---- 403

XP_013910823.1 IEWLNKNL---- 403

XP_015275105.1 IEWLNENL---- 404

XP_015669807.1 IEWLNKNL---- 403

XP_020637486.1 IEWLNNNL---- 403

XP_026520917.1 IEWLNKNL---- 403

XP_026557410.1 IEWLNKNL---- 403

XP_028587147.1 IEWLNKNL---- 402

XP_014425448.1 IEWLNMNL---- 413

XP_021013481.1 LSWLIKNL---- 398

XP_032752628.1 LSWLHKNL---- 398

XP_032887718.1 IDWLQENL---- 404

XP_029884721.1 IEWLNENL---- 403

XP_034357074.1 FNWLIKNL---- 398

XP_032048897.1 IAWLNENL---- 403

XP_008498425.2 IEWLDQNL---- 403

XP_030810306.1 VEWLKENL---- 404

XP_006189146.2 ISWLDENL---- 399

XP_032924696.1 LGWLRDNL---- 403

XP_032630208.1 IEWLNINL---- 403

XP_032554164.1 LQWLKENL---- 403

XP_006868037.1 ISWLRENL---- 399

XP_031975985.1 MDWLSENL---- 403

XP_035422447.1 TEWLNENL---- 403

XP_033814578.1 LNWLNEKL---- 403

XP_035931406.1 MNWLSENL---- 399

XP_032028132.1 IEWLKENL---- 399

XP_033006343.1 IEWLNKNL---- 402

XP_017934775.3 VQWLKENL---- 405

XP_031231873.1 WSWLIKNL---- 398

XP_005141224.2 IEWLNENL---- 403

XP_036244957.1 VDWLKENL---- 403

XP_036207699.1 LSWLSENL---- 399

XP_012365981.2 IEWLKENL---- 399

XP_036045372.1 LSWLMKNL---- 399

XP_008950113.1 IEWLKENL---- 399

XP_034295103.1 IEWLNKNL---- 403

XP_003894989.2 IEWLKENL---- 399

XP_031459500.1 LQWLEEHL---- 403

XP_032264231.1 MNWLSENL---- 399

XP_036301415.1 LSWLSENL---- 399

XP_029472042.1 INWLNEKL---- 402

XP_032118892.1 IEWLKENL---- 399

XP_030351736.1 IEWLNENL---- 403


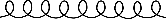


XP_030136362.2 VGWLRENL---- 403

XP_037380609.1 ISWLHENL---- 399

XP_034637747.1 IEWLNINL---- 403

XP_033060172.1 IEWLKENL---- 399

XP_032842734.1 VEWLNENL---- 403

XP_034989473.1 IEWLNKNL---- 402

XP_005525205.1 LDWLKEHL---- 403

XP_009277639.1 IKWLNENL---- 403

XP_009332236.1 IKWLNENL---- 403

XP_009507799.1 IEWLNENL---- 403

XP_009565977.1 IEWLNENL---- 403

XP_009575589.1 IGWLNENL---- 403

XP_009632710.1 IEWLNENL---- 404

XP_009674653.1 INWLNDNL---- 403

XP_009818640.1 IEWLNENL---- 403

XP_009882692.1 IEWLNENL---- 403

XP_009922085.1 VEWLNENL---- 403

XP_009940690.1 IEWLNENL---- 403

XP_010017334.1 IEWLNENL---- 403

XP_010157376.1 IKWLDENL---- 403

XP_010171133.1 LEWLDENL---- 403

XP_010190764.1 MAWLNENL---- 403

XP_010296603.1 IEWLNENL---- 403

XP_010583207.1 VEWLNENL---- 403

XP_013054760.1 IEWLNENL---- 403

XP_013797768.1 IKWLNDNL---- 403

XP_014801236.1 IEWLNENL---- 403

XP_015493839.1 LGWLKEHL---- 403

XP_015727689.1 LQWLEEHL---- 403

XP_017690958.1 MQWLKENL---- 403

XP_021251550.1 LQWLEEHL---- 403

XP_021403179.1 VGWLRENL---- 403

XP_023788489.1 LDWLKEHL---- 403

XP_025920950.1 IKWLNDNL---- 403

XP_026711093.1 IEWLNENL---- 403

XP_027503068.1 VQWLKENL---- 403

XP_027541513.1 LGWLNENL---- 403

XP_027587074.1 VQWLKENL---- 403

XP_027747710.1 VEWLKENL---- 403

**Fig. S2.** The complete motifs analysis using MEME-suite of AADAC orthologues.


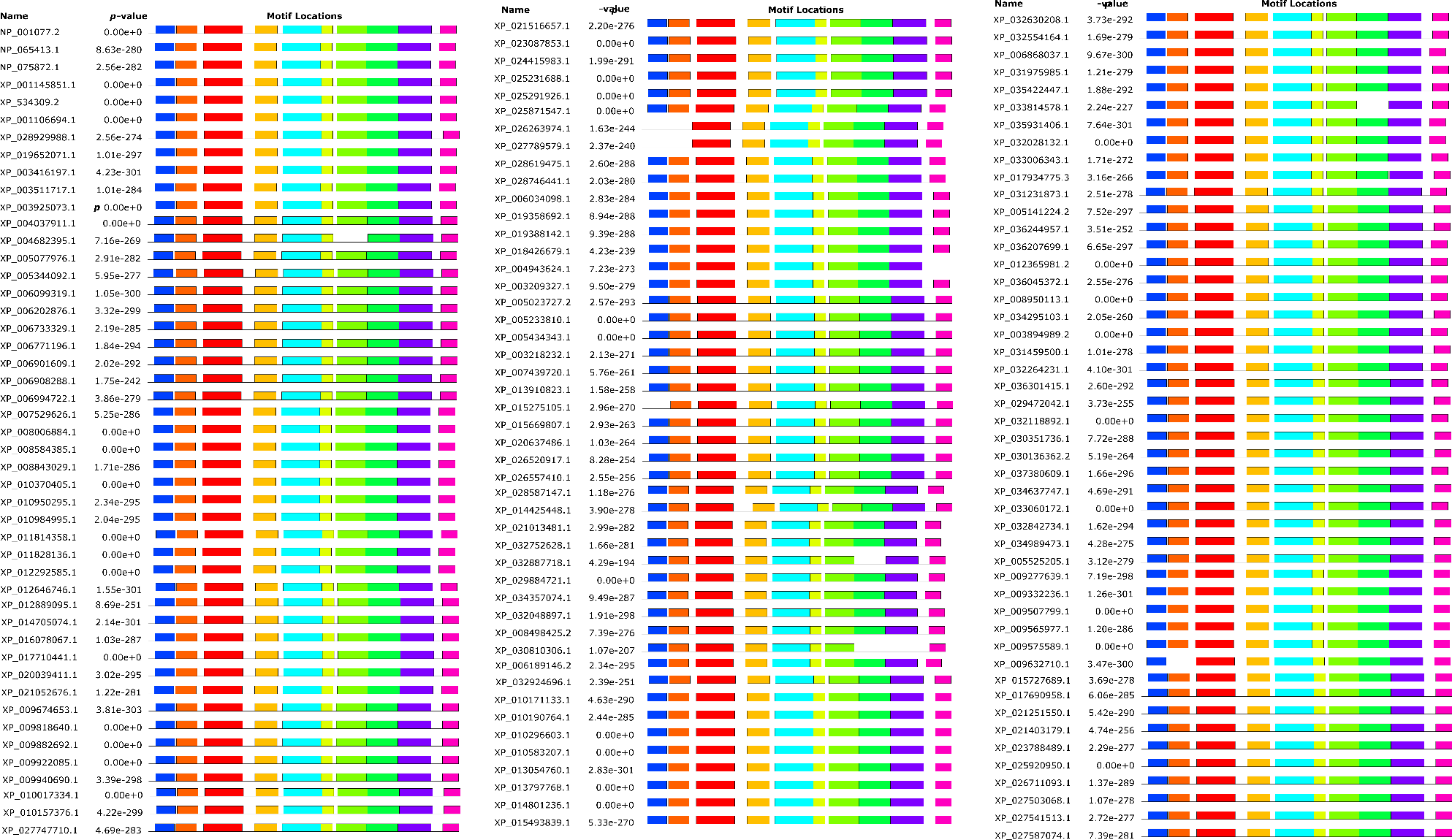


**Table S1.** Summary of the physicochemical characterization, transmembrane regions and N-glycosilation sites predicted for AADAC orthologues.

| **Gene - Accesion number** | **Protein - Accession number** | **Taxonomy** | **Scientific name** | **Molecular mass (kDa)** | **Theorical pI** | **GRAVY** | **Aliphatic index** | **Inestability index** | **Total number of positively charged residues (Arg + Lys)** | **Total number of negatively charge dresidues (Asp + Glu)** | **Predicted transmembrane regions** | **Probable N-glycosilation sites** |
| --- | --- | --- | --- | --- | --- | --- | --- | --- | --- | --- | --- | --- |
| XM_019796512.1 | XP_018426679.1 | Amphibian | *Nanorana parkeri* | 46.07 | 8.3 | -0.04 | 96.95 | 39.93 | 40 | 38 | 0 | 78 (NVTV) |
| XM_006034036.3 | XP_029472042.1 | Amphibian | *Rhinatrema bivittatum* | 45.62 | 6.9 | -0.09 | 96.79 | 49.06 | 41 | 42 | 0 | 78 (NVTV) |
| XM_033031827.1 | XP_033814578.1 | Amphibian | *Geotrypetes seraphini* | 45.90 | 6.2 | -0.12 | 92.95 | 46.07 | 41 | 46 | 1 | 78 (NITV) |
| XM_005023670.5 | XP_032887718.1 | Amphibian | *Amblyraja radiata* | 45.75 | 6.0 | -0.05 | 92.62 | 39.89 | 41 | 46 | 1 | 78 (NVTV) |
| XM_003218184.3 | XP_030810306.1 | Bird | *Camarhynchus parvulus* | 44.05 | 9.2 | 0.07 | 98.61 | 53.81 | 43 | 34 | 0 | 78 (NVTV) |
| XM_013199306.1 | XP_036244957.1 | Bird | *Molothrus ater* | 44.08 | 8.6 | 0.09 | 100.79 | 50.17 | 40 | 36 | 0 | 78 (NVTV) |
| XM_010172831.1 | XP_017690958.1 | Bird | *Lepidothrix coronata* | 44.90 | 8.4 | 0.00 | 95.41 | 43.73 | 40 | 37 | 0 | 78 (NVTV) |
| XM_012437162.2 | XP_027503068.1 | Bird | *Corapipo altera* | 44.98 | 8.2 | 0.02 | 97.82 | 47.44 | 41 | 39 | 1 | 78 (NVTV) |
| XM_009279364.1 | XP_032554164.1 | Bird | *Chiroxiphia lanceolata* | 44.96 | 7.7 | 0.01 | 99.03 | 46.83 | 40 | 39 | 1 | 78 (NVTV) |
| XM_013942314.1 | XP_027747710.1 | Bird | *Empidonax traillii* | 45.09 | 7.7 | 0.02 | 94.42 | 45.05 | 40 | 39 | 0 | 78 (NVTV), 282 (NWSQ) |
| XM_026065165.1 | XP_004943624.1 | Bird | *Gallus gallus* | 44.78 | 7.6 | 0.08 | 94.18 | 38.77 | 37 | 36 | 1 | 78 (NVTV) |
| XM_030028861.1 | XP_017934775.3 | Bird | *Manacus vitellinus* | 45.10 | 6.9 | -0.02 | 95.41 | 48.33 | 39 | 40 | 0 | 78 (NVTV) |
| XM_034501183.1 | XP_027587074.1 | Bird | *Pipra filicauda* | 44.82 | 6.9 | 0.03 | 97.37 | 47.02 | 38 | 39 | 1 | 78 (NVTV) |
| XM_026855292.1 | XP_032048897.1 | Bird | *Aythya fuligula* | 45.16 | 6.9 | -0.01 | 94.07 | 43.18 | 38 | 39 | 0 | 78 (NVTV) |
| XM_032193006.1 | XP_031975985.1 | Bird | *Corvus moneduloides* | 44.80 | 6.8 | -0.01 | 96.10 | 45.85 | 38 | 40 | 0 | 78 (NVTV) |
| XM_010298301.1 | XP_030136362.2 | Bird | *Taeniopygia guttata* | 44.48 | 6.6 | 0.09 | 102.95 | 52.91 | 39 | 41 | 0 | 78 (NVTV), 282 (NWSR) |
| XM_014945750.1 | XP_015493839.1 | Bird | *Parus major* | 44.87 | 6.6 | 0.02 | 95.19 | 60.68 | 38 | 41 | 0 | 78 (NVTV), 282 (NWSR) |
| XM_008500203. | XP_035422447.1 | Bird | *Cygnus atratus* | 45.16 | 6.6 | -0.06 | 91.19 | 44.94 | 39 | 41 | 0 | 78 (NITV) |
| XM_030954446.1 | XP_021403179.1 | Bird | *Lonchura striata domestica* | 44.20 | 6.6 | 0.08 | 101.74 | 49.66 | 39 | 41 | 0 | 78 (NVTV), 282 (NWSR) |
| XM_010951993.1 | XP_021251550.1 | Bird | *Numida meleagris* | 44.84 | 6.5 | -0.02 | 92.23 | 49.86 | 35 | 38 | 1 | 78 (NVTV) |
| XM_010986693.2 | XP_009575589.1 | Bird | *Fulmarus glacialis* | 45.06 | 6.5 | -0.06 | 91.71 | 44.47 | 38 | 41 | 0 | 78 (NVTV), 282 (NWSN) |
| XM_006189084.3 | XP_003209327.1 | Bird | *Meleagris gallopavo* | 44.73 | 6.5 | 0.02 | 89.85 | 44.19 | 35 | 38 | 1 | 78 (NVTV) |
| XM_025436141.2 | XP_010190764.1 | Bird | *Mesitornis unicolor* | 44.56 | 6.5 | 0.06 | 96.33 | 45.29 | 31 | 36 | 1 | 78 (NVTV) |
| XM_534309.7 | XP_005525205.1 | Bird | *Pseudopodoces humilis* | 44.93 | 6.5 | 0.01 | 93.95 | 56.94 | 38 | 42 | 0 | 78 (NVTV), 282 (NWSR) |
| XM_020183822.1 | XP_008498425.2 | Bird | *Calypte anna* | 45.60 | 6.4 | -0.08 | 95.78 | 46.97 | 38 | 42 | 0 | 78 (NITV), 88 (NVSV) |
| XM_033068805.1 | XP_009632710.1 | Bird | *Egretta garzetta* | 45.41 | 6.4 | 0.00 | 99.16 | 41.24 | 37 | 41 | 2 | 78 (NVTV) |
| XM_009884390.1 | XP_023788489.1 | Bird | *Cyanistes caeruleus* | 45.01 | 6.4 | 0.01 | 94.69 | 58.06 | 38 | 43 | 0 | 78 (NVTV), 282 (NWSR) |
| XM_032774317.1 | XP_009277639.1 | Bird | *Aptenodytes forsteri* | 45.44 | 6.3 | 0.01 | 100.65 | 42.21 | 39 | 43 | 1 | 78 (NVTV) |
| XM_032698273.1 | XP_005023727.2 | Bird | *Anas platyrhynchos* | 45.06 | 6.3 | -0.02 | 92.61 | 43.53 | 37 | 41 | 0 | 78 (NVTV) |
| XM_008008693.2 | XP_005141224.2 | Bird | *Melopsittacus undulatus* | 45.84 | 6.3 | -0.15 | 92.41 | 44.95 | 39 | 43 | 0 | 78 (NVTV), 88 (NTTV) |
| XM_006867975.1 | XP_030351736.1 | Bird | *Strigops habroptila* | 45.52 | 6.3 | -0.03 | 97.72 | 45.10 | 38 | 42 | 0 | 78 (NVTV) |
| XM_011958968.1 | XP_032924696.1 | Bird | *Catharus ustulatus* | 44.62 | 6.2 | 0.08 | 96.60 | 50.44 | 33 | 39 | 1 | 78 (NVTV), 282 (NWSL) |
| XM_004682338.1 | XP_013797768.1 | Bird | *Apteryx australis mantelli* | 45.24 | 6.2 | -0.12 | 91.19 | 39.48 | 37 | 42 | 1 | 78 (NVTV) |
| XM_027647267.1 | XP_025920950.1 | Bird | *Apteryx rowi* | 45.24 | 6.2 | -0.12 | 91.19 | 39.48 | 37 | 42 | 1 | 78 (NVTV) |
| XM_032120094.1 | XP_026711093.1 | Bird | *Athene cunicularia* | 44.84 | 6.2 | 0.04 | 94.89 | 40.35 | 32 | 37 | 1 | 78 (NVTV) |
| XM_015872203.2 | XP_015727689.1 | Bird | *Coturnix japonica* | 44.99 | 6.2 | -0.02 | 91.51 | 43.30 | 35 | 40 | 1 | 78 (NVTV) |
| XM_003511669.3 | XP_031459500.1 | Bird | *Phasianus colchicus* | 44.71 | 6.2 | 0.05 | 93.23 | 44.00 | 33 | 38 | 1 | 78 (NVTV) |
| XM_019532597.1 | XP_010157376.1 | Bird | *Eurypyga helias* | 44.92 | 6.2 | -0.02 | 95.14 | 41.60 | 37 | 43 | 0 | 78 (NVTV) |
| XM_009567682.1 | XP_009332236.1 | Bird | *Pygoscelis adeliae* | 45.27 | 6.1 | -0.08 | 96.05 | 42.37 | 38 | 43 | 1 | 78 (NVTV) |
| XM_023932721.1 | XP_009674653.1 | Bird | *Struthio camelus australis* | 45.34 | 6.1 | -0.05 | 97.74 | 44.01 | 34 | 41 | 1 | 78 (NVTV) |
| XM_035566554.1 | XP_013054760.1 | Bird | *Anser cygnoides domesticus* | 45.10 | 6.1 | -0.03 | 92.85 | 44.79 | 37 | 42 | 0 | 78 (NVTV) |
| XM_024560215.1 | XP_010296603.1 | Bird | *Balearica regulorum gibbericeps* | 45.17 | 6.1 | 0.01 | 98.96 | 42.05 | 35 | 42 | 0 | 78 (NVTV) |
| XM_013033641.1 | XP_029884721.1 | Bird | *Aquila chrysaetos chrysaetos* | 45.20 | 6.0 | -0.04 | 94.14 | 38.52 | 36 | 43 | 0 | 78 (NVTV) |
| XM_009634415.2 | XP_010017334.1 | Bird | *Nestor notabilis* | 45.68 | 6.0 | -0.06 | 95.56 | 41.57 | 36 | 44 | 1 | 78 (NVTV), 88 (NITV) |
| XM_006901547.1 | XP_009940690.1 | Bird | *Opisthocomus hoazin* | 45.18 | 6.0 | 0.00 | 92.66 | 42.71 | 34 | 42 | 1 | 78 (NVTV), 282 (NWSN) |
| XM_027891909.1 | XP_009565977.1 | Bird | *Cuculus canorus* | 44.95 | 5.9 | 0.01 | 96.10 | 42.89 | 35 | 42 | 1 | 78 (NVTV) |
| XM_014849588.1 | XP_032842734.1 | Bird | *Tyto alba alba* | 44.61 | 5.9 | 0.05 | 98.59 | 39.72 | 33 | 41 | 1 | 78 (NVTV) |
| XM_007529564.2 | XP_014801236.1 | Bird | *Calidris pugnax* | 45.18 | 5.9 | -0.03 | 95.36 | 41.97 | 35 | 44 | 1 | 78 (NVTV) |
| XM_010159074.1 | XP_027541513.1 | Bird | *Neopelma chrysocephalum* | 44.91 | 5.9 | 0.06 | 95.61 | 47.68 | 34 | 43 | 0 | No sites |
| XM_005434286.3 | XP_009922085.1 | Bird | *Haliaeetus albicilla* | 45.23 | 5.9 | -0.01 | 95.58 | 36.32 | 36 | 44 | 0 | 78 (NVTV) |
| XM_005233753.3 | XP_010583207.1 | Bird | *Haliaeetus leucocephalus* | 45.23 | 5.9 | -0.01 | 95.58 | 36.32 | 36 | 44 | 0 | 78 (NVTV) |
| XM_009577294.1 | XP_009507799.1 | Bird | *Phalacrocorax carbo* | 45.01 | 5.9 | -0.03 | 93.85 | 42.58 | 35 | 43 | 1 | 78 (NVTV) |
| XM_008586163.1 | XP_009882692.1 | Bird | *Charadrius vociferus* | 45.17 | 5.8 | -0.02 | 92.48 | 43.44 | 33 | 43 | 0 | 78 (NVTV) |
| XM_422836.6 | XP_010171133.1 | Bird | *Antrostomus carolinensis* | 45.33 | 5.8 | -0.04 | 95.11 | 41.00 | 36 | 44 | 0 | 78 (NVTV) |
| XM_009820338.1 | XP_005434343.1 | Bird | *Falco cherrug* | 45.46 | 5.8 | -0.01 | 98.98 | 49.40 | 35 | 45 | 1 | 78 (NVTV) |
| XM_019503147.1 | XP_005233810.1 | Bird | *Falco peregrinus* | 45.46 | 5.8 | -0.01 | 98.98 | 49.40 | 35 | 45 | 1 | 78 (NVTV) |
| XM_015419619.1 | XP_009818640.1 | Bird | *Gavia stellata* | 45.03 | 5.6 | 0.00 | 94.12 | 43.41 | 32 | 43 | 0 | 78 (NVTV) |
| XM_033958687.1 | XP_006908288.1 | Mammal | *Pteropus alecto* | 45.93 | 9.3 | -0.13 | 106.01 | 44.71 | 48 | 38 | 1 | 78 (NITV), 281 (NWSS), 298 (NRTY) |
| XM_004037863.3 | XP_036207699.1 | Mammal | *Myotis myotis* | 45.32 | 9.1 | -0.15 | 92.06 | 42.98 | 44 | 37 | 3 | 282 (NWSS) |
| XM_028763642.1 | XP_006771196.1 | Mammal | *Myotis davidii* | 45.22 | 9.1 | -0.10 | 95.96 | 38.97 | 43 | 36 | 3 | 282 (NWSS) |
| XM_009923783.1 | XP_006099319.1 | Mammal | *Myotis lucifugus* | 45.24 | 8.9 | -0.13 | 93.03 | 40.58 | 43 | 38 | 2 | 282 (NWSS) |
| XM_010584905.1 | NP_001077.2 | Mammal | *Homo sapiens* | 45.73 | 8.8 | -0.12 | 99.15 | 33.96 | 44 | 40 | 1 | 78 (NVTV), 282 (NWSS) |
| XM_036075513.1 | XP_006202876.1 | Mammal | *Vicugna pacos* | 46.04 | 8.7 | -0.20 | 91.35 | 32.93 | 43 | 39 | 0 | 78 (NVTV), 282 (NWSS) |
| NM_001086.3 | XP_027789579.1 | Mammal | *Marmota flaviventris* | 44.97 | 8.7 | -0.08 | 97.74 | 46.77 | 46 | 42 | 1 | 276 (NWSS) |
| XM_032172241.1 | XP_031231873.1 | Mammal | *Mastomys coucha* | 45.62 | 8.7 | -0.06 | 97.46 | 45.71 | 40 | 36 | 3 | 77 (NVTV) |
| XM_033150452.1 | XP_006901609.1 | Mammal | *Elephantulus edwardii* | 45.53 | 8.6 | -0.14 | 91.85 | 38.73 | 41 | 38 | 1 | 78 (NVTV), 127 (NLSR) |
| XM_017835469.1 | XP_008950113.1 | Mammal | *Pan paniscus* | 45.76 | 8.6 | -0.12 | 99.40 | 34.60 | 44 | 41 | 1 | 78 (NVTV), 282 (NWSS) |
| XM_006733266.1 | XP_016078067.1 | Mammal | *Miniopterus natalensis* | 45.68 | 8.6 | -0.13 | 96.69 | 39.88 | 44 | 41 | 1 | 78 (NITV), 282 (NWSS) |
| XM_021547504.1 | XP_012365981.2 | Mammal | *Nomascus leucogenys* | 45.78 | 8.6 | -0.14 | 98.92 | 36.18 | 44 | 41 | 1 | 78 (NVTV), 282 (NWSS) |
| XM_003416149.1 | XP_028929988.1 | Mammal | *Ornithorhynchus anatinus* | 45.92 | 8.5 | -0.18 | 93.35 | 42.16 | 44 | 41 | 0 | 78 (NITV), 282 (NWSA) |
| XM_001106694.4 | XP_036301415.1 | Mammal | *Pipistrellus kuhlii* | 45.34 | 8.3 | -0.07 | 98.15 | 37.19 | 42 | 40 | 2 | 282 (NWSS) |
| XM_018079286.3 | XP_004037911.1 | Mammal | *Gorilla gorilla gorilla* | 45.72 | 8.3 | -0.11 | 100.38 | 34.14 | 43 | 41 | 1 | 78 (NVTV), 282 (NWSS) |
| XM_011972746.1 | XP_001145851.1 | Mammal | *Pan troglodytes* | 45.74 | 8.3 | -0.12 | 99.40 | 34.71 | 43 | 41 | 1 | 78 (NVTV), 282 (NWSS) |
| XM_027933778.1 | XP_003925073.1 | Mammal | *Saimiri boliviensis boliviensis* | 46.00 | 8.3 | -0.15 | 96.97 | 37.61 | 42 | 40 | 1 | 78 (NVTV), 282 (NWSS) |
| XM_031376013.1 | XP_010950295.1 | Mammal | *Camelus bactrianus* | 45.92 | 8.3 | -0.19 | 92.08 | 33.66 | 42 | 40 | 0 | 78 (NVTV), 282 (NWSS) |
| XM_003209279.4 | XP_006189146.2 | Mammal | *Camelus ferus* | 45.92 | 8.3 | -0.19 | 92.08 | 33.66 | 42 | 40 | 0 | 78 (NVTV), 282 (NWSS) |
| XM_005141167.3 | XP_032118892.1 | Mammal | *Sapajus apella* | 45.85 | 8.3 | -0.14 | 95.76 | 41.05 | 43 | 41 | 1 | 78 (NVTV), 282 (NWSS) |
| XM_021660982.1 | XP_032028132.1 | Mammal | *Hylobates moloch* | 45.74 | 8.2 | -0.12 | 99.17 | 35.36 | 43 | 41 | 1 | 78 (NVTV), 282 (NWSS) |
| XM_010192462.1 | XP_006733329.1 | Mammal | *Leptonychotes weddellii* | 45.91 | 8.2 | -0.10 | 92.08 | 47.93 | 42 | 40 | 3 | 78 (NVTV), 282 (NWSS) |
| XM_005077919.3 | XP_012889095.1 | Mammal | *Dipodomys ordii* | 45.58 | 7.8 | 0.04 | 101.90 | 39.60 | 40 | 39 | 3 | 362 (NVTH) |
| XM_012791292.2 | XP_011828136.1 | Mammal | *Mandrillus leucophaeus* | 45.84 | 7.8 | -0.12 | 102.36 | 40.21 | 43 | 42 | 1 | 78 (NVTV), 282 (NWSS) |
| XM_005344035.2 | XP_021013481.1 | Mammal | *Mus caroli* | 45.39 | 7.8 | -0.11 | 93.99 | 41.43 | 40 | 39 | 1 | 77 (NVTV) |
| XM_016222581.1 | XP_003894989.2 | Mammal | *Papio anubis* | 45.85 | 7.8 | -0.11 | 103.81 | 39.40 | 43 | 42 | 1 | 78 (NVTV), 282 (NWSS) |
| XM_036389064.1 | XP_008006884.1 | Mammal | *Chlorocebus sabaeus* | 45.70 | 7.8 | -0.13 | 101.60 | 38.01 | 43 | 42 | 1 | 78 (NVSV), 282 (NWSS) |
| XM_029475372.1 | XP_011814358.1 | Mammal | *Colobus angolensis palliatus* | 46.25 | 7.8 | -0.12 | 102.54 | 41.02 | 43 | 42 | 1 | 81 (NVTV), 285 (NWSS) |
| NM_023383.1 | XP_021052676.1 | Mammal | *Mus pahari* | 45.37 | 7.8 | -0.11 | 95.98 | 43.84 | 40 | 39 | 2 | 77 (NVTV) |
| XM_021197017.2 | XP_023087853.1 | Mammal | *Piliocolobus tephrosceles* | 45.83 | 7.8 | -0.12 | 103.31 | 41.09 | 43 | 42 | 1 | 78 (NVTV), 282 (NWSS) |
| XM_006771133.2 | XP_033060172.1 | Mammal | *Trachypithecus francoisi* | 45.94 | 7.8 | -0.14 | 100.63 | 42.73 | 43 | 42 | 1 | 78 (NVTV), 282 (NWSS) |
| XM_006099257.3 | XP_010984995.1 | Mammal | *Camelus dromedarius* | 45.88 | 7.7 | -0.19 | 92.33 | 33.73 | 42 | 41 | 0 | 78 (NVTV), 282 (NWSS) |
| XM_036351806.1 | XP_026263974.1 | Mammal | *Urocitellus parryii* | 44.77 | 7.7 | 0.02 | 101.90 | 46.13 | 42 | 41 | 3 | 276 (NWSS) |
| XM_008844807.2 | XP_006868037.1 | Mammal | *Chrysochloris asiatica* | 46.08 | 7.7 | -0.17 | 94.06 | 43.23 | 42 | 41 | 1 | 78 (NVTV), 282 (NWSS) |
| XM_018571177.1 | XP_035931406.1 | Mammal | *Halichoerus grypus* | 45.85 | 7.7 | -0.12 | 92.06 | 45.91 | 42 | 41 | 3 | 78 (NVTV), 282 (NWSS) |
| XM_027685712.1 | XP_037380609.1 | Mammal | *Talpa occidentalis* | 45.72 | 7.3 | -0.08 | 98.92 | 42.01 | 40 | 40 | 1 | 78 (NVTV), 282 (NWSS) |
| XM_010019032.1 | NP_065413.1 | Mammal | *Rattus norvegicus* | 45.69 | 7.3 | -0.11 | 98.17 | 42.91 | 38 | 38 | 3 | 77 (NVTV) |
| XM_012510527.2 | XP_008584385.1 | Mammal | *Galeopterus variegatus* | 45.68 | 7.3 | -0.07 | 96.22 | 41.48 | 38 | 38 | 2 | 78 (NVTV), 282 (NWSS) |
| XM_026665132.1 | XP_025231688.1 | Mammal | *Theropithecus gelada* | 45.87 | 7.2 | -0.12 | 102.83 | 40.40 | 42 | 42 | 1 | 78 (NVTV) |
| XM_021395875.1 | XP_024415983.1 | Mammal | *Desmodus rotundus* | 45.65 | 7.2 | -0.15 | 95.24 | 38.68 | 38 | 38 | 0 | 78 (NVTV), 282 (NWSS), 302 (NGTS) |
| XM_036189479.1 | XP_032264231.1 | Mammal | *Phoca vitulina* | 45.75 | 7.1 | -0.10 | 92.56 | 44.66 | 41 | 41 | 3 | 78 (NVTV), 282 (NWSS) |
| XM_009942388.1 | NP_075872.1 | Mammal | *Mus musculus* | 45.25 | 7.0 | -0.11 | 94.72 | 44.25 | 39 | 40 | 0 | No sites |
| XM_029074155.1 | XP_014705074.1 | Mammal | *Equus asinus* | 45.55 | 7.0 | -0.12 | 93.28 | 33.00 | 39 | 40 | 1 | 78 (NVTV), 282 (NWSS) |
| XM_034957613.1 | XP_032752628.1 | Mammal | *Rattus rattus* | 45.67 | 6.9 | -0.11 | 97.94 | 46.77 | 38 | 39 | 3 | 77 (NVTV) |
| XM_001145851.5 | XP_019652071.1 | Mammal | *Ailuropoda melanoleuca* | 45.76 | 6.9 | -0.12 | 91.35 | 46.77 | 41 | 40 | 3 | 78 (NVTV), 282 (NWSS) |
| XM_034439212.1 | XP_008843029.1 | Mammal | *Nannospalax galili* | 45.76 | 6.9 | -0.17 | 90.10 | 50.58 | 40 | 41 | 0 | 282 (NWSS) |
| XM_003894940.4 | XP_012292585.1 | Mammal | *Aotus nancymaae* | 45.83 | 6.9 | -0.18 | 93.53 | 40.92 | 41 | 42 | 1 | 78 (NVTV), 282 (NWSS) |
| XM_015638353.2 | XP_001106694.1 | Mammal | *Macaca mulatta* | 45.82 | 6.9 | -0.13 | 102.11 | 39.87 | 42 | 43 | 1 | 78 (NVTV), 282 (NWSS) |
| XM_014569962.2 | XP_003416197.1 | Mammal | *Loxodonta africana* | 45.38 | 6.8 | -0.11 | 94.56 | 40.57 | 39 | 40 | 1 | 78 (NVTV) |
| XM_028890608.2 | XP_004682395.1 | Mammal | *Condylura cristata* | 45.90 | 6.8 | -0.13 | 94.32 | 37.18 | 40 | 41 | 1 | 78 (NVTV), 283 (NWSS) |
| XM_006994660.2 | XP_034357074.1 | Mammal | *Arvicanthis niloticus* | 45.65 | 6.7 | -0.10 | 95.48 | 47.01 | 38 | 40 | 0 | 77 (NVTV) |
| XM_009509504.1 | XP_028619475.1 | Mammal | *Grammomys surdaster* | 45.52 | 6.7 | -0.03 | 103.09 | 45.57 | 37 | 39 | 1 | 77 (NVTV), 281 (NWSS) |
| XM_031603640.1 | XP_017710441.1 | Mammal | *Rhinopithecus bieti* | 45.86 | 6.7 | -0.14 | 100.60 | 42.04 | 40 | 42 | 1 | 78 (NVTV), 282 (NWSS) |
| XM_032408340.1 | XP_010370405.1 | Mammal | *Rhinopithecus roxellana* | 45.86 | 6.7 | -0.14 | 100.60 | 42.04 | 40 | 42 | 1 | 78 (NVTV), 282 (NWSS) |
| XM_023232085.2 | XP_005344092.1 | Mammal | *Microtus ochrogaster* | 45.36 | 6.5 | -0.08 | 97.97 | 45.60 | 36 | 39 | 0 | 79 (NVTV) |
| XM_036445522.1 | XP_012646746.1 | Mammal | *Microcebus murinus* | 46.01 | 6.5 | -0.16 | 92.31 | 44.64 | 41 | 44 | 1 | 78 (NVTV), 282 (NWSS) |
| XM_027731273.1 | XP_025291926.1 | Mammal | *Canis lupus dingo* | 45.90 | 6.3 | -0.14 | 89.65 | 42.98 | 40 | 43 | 3 | 78 (NVTV), 282 (NWSS) |
| XM_028731314.1 | XP_534309.2 | Mammal | *Canis lupus familiaris* | 45.90 | 6.3 | -0.14 | 89.65 | 42.98 | 40 | 43 | 3 | 78 (NVTV), 282 (NWSS) |
| XM_020781827.1 | XP_025871547.1 | Mammal | *Vulpes vulpes* | 45.96 | 6.3 | -0.13 | 88.92 | 44.32 | 40 | 43 | 3 | 56 (NHSM), 78 (NVTV), 282 (NWSS) |
| XM_015814321.1 | XP_003511717.1 | Mammal | *Cricetulus griseus* | 45.52 | 6.3 | -0.05 | 97.46 | 44.15 | 36 | 40 | 1 | 77 (NVTV), 281 (NWSS) |
| XM_026701625.1 | XP_021516657.1 | Mammal | *Meriones unguiculatus* | 45.47 | 6.3 | -0.03 | 97.19 | 45.24 | 35 | 41 | 0 | 77 (NVTV), 281 (NWSS) |
| XM_005525148.2 | XP_006994722.1 | Mammal | *Peromyscus maniculatus bairdii* | 45.43 | 6.3 | -0.06 | 95.98 | 42.26 | 36 | 41 | 1 | 77 (NVTV), 281 (NWSS) |
| XM_006908226.1 | XP_005077976.1 | Mammal | *Mesocricetus auratus* | 45.28 | 6.2 | -0.09 | 91.83 | 40.16 | 35 | 40 | 3 | 55 (NHSM), 77 (NVTV), 281 (NWSS) |
| XM_009333961.1 | XP_028746441.1 | Mammal | *Peromyscus leucopus* | 45.30 | 6.2 | -0.07 | 94.77 | 39.85 | 34 | 40 | 1 | 77 (NVTV) |
| XM_007439658.3 | XP_020039411.1 | Mammal | *Castor canadensis* | 45.64 | 6.0 | -0.18 | 86.22 | 40.39 | 38 | 43 | 3 | 78 (NVTV), 282 (NWSS) |
| NM_020538.1 | XP_007529626.1 | Mammal | *Erinaceus europaeus* | 45.53 | 6.0 | -0.15 | 92.31 | 38.10 | 41 | 47 | 0 | 78 (NVSV), 282 (NWSS) |
| XM_032896737.1 | XP_036045372.1 | Mammal | *Onychomys torridus* | 45.67 | 6.0 | -0.04 | 97.22 | 40.67 | 35 | 43 | 1 | 78 (NVTV) |
| XM_029616182.1 | XP_015669807.1 | Reptiles | *Protobothrops mucrosquamatus* | 46.08 | 8.5 | -0.03 | 99.18 | 39.79 | 44 | 41 | 0 | 282 (NWSN) |
| XM_017854952.1 | XP_015275105.1 | Reptiles | *Gekko japonicus* | 45.82 | 8.5 | -0.10 | 94.28 | 36.07 | 43 | 40 | 0 | 283 (NWSN), 385 (NLSV) |
| XM_010372103.1 | XP_034295103.1 | Reptiles | *Pantherophis guttatus* | 45.95 | 7.8 | -0.06 | 99.43 | 38.52 | 42 | 41 | 0 | 282 (NWSN) |
| XM_003925024.2 | XP_033006343.1 | Reptiles | *Lacerta agilis* | 45.14 | 7.0 | -0.07 | 92.64 | 37.30 | 38 | 39 | 0 | No sites |
| XM_032263001.1 | XP_034989473.1 | Reptiles | *Zootoca vivipara* | 45.22 | 6.9 | -0.10 | 90.70 | 39.36 | 40 | 41 | 0 | 282 (NWSR) |
| XM_030495876.1 | XP_026520917.1 | Reptiles | *Notechis scutatus* | 45.89 | 6.9 | -0.05 | 102.08 | 34.85 | 42 | 43 | 1 | 85 (NFSH) |
| XM_009676358.1 | XP_028587147.1 | Reptiles | *Podarcis muralis* | 45.22 | 6.9 | -0.04 | 96.24 | 35.56 | 39 | 40 | 0 | no sites |
| XM_030280502.2 | XP_014425448.1 | Reptiles | *Pelodiscus sinensis* | 46.77 | 6.7 | 0.00 | 100.27 | 47.74 | 41 | 43 | 0 | 78 (NMTV), 292 (NWSN) |
| XM_037524712.1 | XP_007439720.1 | Reptiles | *Python bivittatus* | 45.53 | 6.7 | 0.03 | 104.91 | 41.22 | 39 | 41 | 1 | 282 (NWSH) |
| XM_014055348. | XP_026557410.1 | Reptiles | *Pseudonaja textilis* | 45.84 | 6.5 | -0.03 | 101.84 | 35.14 | 41 | 44 | 0 | No sites |
| XM_025375903.1 | XP_013910823.1 | Reptiles | *Thamnophis sirtalis* | 45.74 | 6.4 | -0.13 | 95.73 | 40.87 | 40 | 44 | 0 | 282 (NWSK) |
| XM_034781856.1 | XP_003218232.1 | Reptiles | *Anolis carolinensis* | 45.83 | 6.3 | -0.08 | 96.95 | 43.66 | 41 | 45 | 0 | 282 (NWSE) |
| XM_033204281.1 | XP_034637747.1 | Reptiles | *Trachemys scripta elegans* | 45.34 | 6.0 | 0.06 | 104.74 | 46.28 | 36 | 43 | 0 | 78 (NVTV), 282 (NWSN) |
| XM_032986843.1 | XP_032630208.1 | Reptiles | *Chelonoidis abingdonii* | 45.61 | 5.9 | 0.05 | 103.25 | 47.49 | 36 | 44 | 0 | 78 NVTV |
| XM_026408189.1 | XP_020637486.1 | Reptiles | *Pogona vitticeps* | 45.18 | 5.8 | -0.01 | 103.50 | 33.92 | 36 | 45 | 0 | 127 (NLSR), 282 (NWSN) |
| XM_006202814.3 | XP_006034098.1 | Reptiles | *Alligator sinensis* | 45.62 | 5.6 | -0.14 | 94.86 | 47.33 | 37 | 48 | 1 | 78 (NVTV), 282 (NWSN) |
| XM_026015762.1 | XP_019388142.1 | Reptiles | *Crocodylus porosus* | 45.53 | 5.6 | -0.12 | 92.43 | 48.36 | 36 | 47 | 0 | 78 (NVTV), 282 (NWSN) |
| XM_035133582.1 | XP_019358692.1 | Reptiles | *Gavialis gangeticus* | 45.47 | 5.5 | -0.09 | 94.34 | 47.21 | 34 | 46 | 1 | 78 (NVTV), 282 (NWSN) |
